# Significant alteration of liver metabolites by AAV8.Urocortin 2 gene transfer in mice with insulin resistance

**DOI:** 10.1371/journal.pone.0224428

**Published:** 2019-12-02

**Authors:** Young Chul Kim, Agnieszka D. Truax, Dimosthenis Giamouridis, N. Chin Lai, Tracy Guo, H. Kirk Hammond, Mei Hua Gao

**Affiliations:** 1 Veterans Affairs San Diego Healthcare System, San Diego, California, United States of America; 2 Department of Medicine, University of California San Diego, San Diego, California, United States of America; 3 Metabolon, Inc, Research Triangle Park, Morrisville, North Carolina, United States of America; Max Delbruck Centrum fur Molekulare Medizin Berlin Buch, GERMANY

## Abstract

**Introduction:**

Urocortin 2 (Ucn2) is a 38-amino acid peptide of the corticotropin-releasing factor family. Intravenous (IV) delivery of an adeno-associated virus vector serotype 8 encoding *Ucn2* (AAV8.*Ucn2*) increases insulin sensitivity and glucose disposal in mice with insulin resistance.

**Objective:**

To determine the effects of Ucn2 on liver metabolome.

**Methods:**

Six-week-old C57BL6 mice were divided into normal chow (CHOW)-fed and high fat diet (HFD)-fed groups. The animals received saline, AAV8 encoding no gene (AAV8.Empt) or AAV8.*Ucn2* (2x10^13^ genome copy/kg, IV injection). Livers were isolated from CHOW-fed and HFD-fed mice and analyzed by untargeted metabolomics. Group differences were statistically analyzed.

**Results:**

In CHOW-fed mice, AAV8.*Ucn2* gene transfer (vs. saline) altered the metabolites in glycolysis, pentose phosphate, glycogen synthesis, glycogenolysis, and choline-folate-methionine signaling pathways. In addition, AAV8.*Ucn2* gene transfer increased amino acids and peptides, which were associated with reduced protein synthesis. In insulin resistant (HFD-induced) mice, HFD (vs CHOW) altered 448 (112 increased and 336 decreased) metabolites and AAV8.*Ucn2* altered 239 metabolites (124 increased and 115 reduced) in multiple pathways. There are 61 metabolites in 5 super pathways showed interactions between diet and AAV8.*Ucn2* treatment. Among them, AAV8.*Ucn2* gene transfer reversed HFD effects on 13 metabolites. Finally, plasma Ucn2 effects were determined using a 3-group comparison of HFD-fed mice that received AAV8.*Ucn2*, AAV.Empt or saline, where 18 metabolites that altered by HFD (15 increased and 3 decreased), but restored levels to that seen in CHOW-fed mice by increased plasma Ucn2.

**Conclusions:**

Metabolomics study revealed that AAV8.Ucn2 gene transfer, through increased plasma Ucn2, provided counter-HFD effects in restoring hepatic metabolites to normal levels, which could be the underlying mechanisms for Ucn2 effects on increasing glucose disposal and reducing insulin assistance.

## Introduction

The corticotropin-releasing factor family (CRF) consists of 3 members, Ucn1, Ucn2, and Ucn3. Ucn1 binds to the corticotropin-releasing factor family receptor 1 (CRFR1), but Ucn2 and Ucn3 selectively bind to CRFR2 and promotes stress-coping responses via CRFR2-mediated anxiolytic effects [1, 2]. CRFR1 is mainly expressed in the brain and pituitary gland, whereas CRFR2 in mice is found not only in central nervous system but also in peripheral tissues, such as the heart, liver, pancreas and skeletal muscle [3–5]. Activation of CRFR2 signaling axis by Ucn2 and Ucn3 mediates a broad range of physiological responses include energy balance, cardiac function, and glucose metabolism [6–9]. Deletion of gene for CRFR2 developed impaired glucose tolerance on normal-Chow-fed male, but not female mice [10].

Previous studies demonstrated that adeno-associated viral vector serotype 8 mediated *Ucn2* (AAV8.*Ucn2*) expression increased plasma Ucn2 >15 fold, which persists at least 7 months, increases left ventricular function of normal and failing heart, and reduces the adverse effects of a Western diet on cardiac function in mice [11–13]. AAV8.*Ucn2* gene transfer also increases insulin sensitivity and glucose disposal in insulin resistant mice, effects were abolished in CRFR2 deleted mice [9]. Interestingly, unlike *Ucn2*, AAV8.*Ucn3* gene transfer has no effects on glucose disposal, although it increased cardiac function [14]. In addition to increasing skeletal muscle glucose uptake, Ucn2 gene transfer decreases hepatic glucose production and reduces fatty infiltration of liver in mice rendered insulin resistant by HFD [15]. These data indicate that *Ucn2* gene transfer alters liver metabolism in restoring insulin sensitivity in HFD-fed mice.

To understand how the liver responds to *Ucn2* gene transfer, we used untargeted metabolomics to determine metabolites that are altered in normal and in insulin-resistant mice.

## Materials and methods

### Animal use

**Fig 1:**
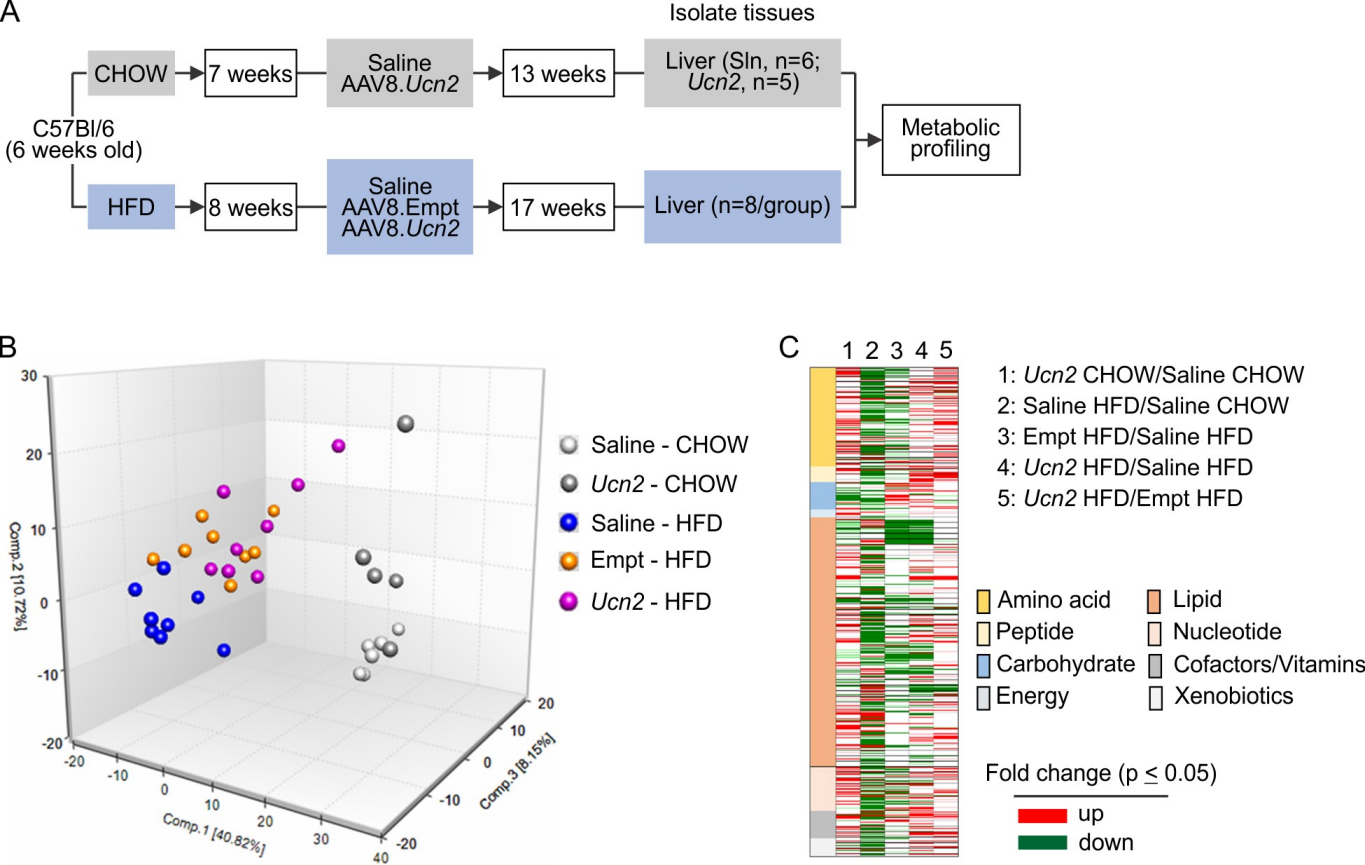
Study design, metabolomics library, principal component and statistical heatmap analysis. **A.** Study design and experimental timelines. **B.** Principal Component Analysis (PCA) showed distinct metabolomic profiles between samples isolated from livers of CHOW-fed and HFD-fed mice. **C.** Statistical heat map of comparisons between groups. A8, AAV8; CHOW, normal Chow; HFD, high fat diet.

Thirty-six C57BL/6 male mice (6 weeks old) were obtained from The Jackson Laboratory. Mice were fed either a cereal-based normal Chow for 7 weeks (CHOW, Harlan Teklad Lab) or High Fat Diet (HFD,60 kcal%; Research Diets, 8 weeks) ad lib and received either saline, AAV8.Empt, or AAV8.*Ucn2* (2x10^13^ gc/kg) via intravenous (iv) injection as indicated in the schematics (**Fig 1A**). Liver tissues were collected 13 weeks (CHOW group) or 17 weeks (HFD group) after gene transfer. All animal procedures were approved by the VA San Diego Health System IACUC and complied with the guidelines.

### AAV8 vector production and gene transfer

AAV8 vector encoding murine *Ucn2* gene driven by a chicken β-actin promoter and control empt (scrambled DNAs) vector were previously described [12]. Viral vector (2x10^13^ gc/kg body weight) in 100 μl of volume or similar volume of saline was delivered via jugular vein under anesthesia.

### Sample preparation and mass spectrometry analysis for global metabolomics

Liver was excised, immediately frozen and stored at -80°C until processed. Sample preparation was carried out as described previously at Metabolon, Inc. [16]. Briefly, samples were homogenized and subjected to methanol extraction. Samples were split into aliquots for analysis by ultrahigh performance liquid chromatography/mass spectrometry (UHPLC/MS). The four aliquots used in the studies are for conditions of 1) acidic positive ion conditions, chromatographically optimized for more hydrophilic compounds; 2) acidic positive ion conditions, chromatographically optimized for more hydrophobic compounds; 3) basic negative ion optimized conditions using a separate dedicated C18 column; 4) negative ionization following elution from a HILIC column and the fifth aliquot was reserved for backup. Metabolites were identified by automated comparison of the ion features in the experimental samples to a reference library of chemical standard entries that included retention time, molecular weight (m/z), preferred adducts, and in-source fragments as well as associated MS Spectra and curated by visual inspection for quality control using software developed at Metabolon. Identification of known chemical entities is based on comparison to metabolomic library entries of purified standards. As a result, Metabolon was able to identify 714 biochemicals representing 9 super pathways and 88 sub pathways (**Table 1**).

**Table 1:** Liver metabolite library.

Super Pathways	Sub Pathways	Identified Metabolites
Amino Acid	15	144
Peptide	3	23
Carbohydrate	8	40
Energy	2	12
Lipid	44	364
Nucleotide	9	65
Cofector & Vitamin	12	40
Xenobiotic	5	26
**Total**	**88**	**714**

A variety of curation procedures were carried out as previously described [17] to ensure that a high-quality data set was made available for statistical analysis and data interpretation.

Statistical tests were performed in ArrayStudio (Omicsoft) to compare data between experimental groups; p < .05 is considered significant. An estimate of the false discovery rate (Q-value) is also calculated to take into account the multiple comparisons that normally occur in metabolomic-based studies, with Q < .05 used as false positive rate when there is one test.

### In vivo protein synthesis monitoring

SUnSet (surface sensing of translation) was performed to monitor protein synthesis *in vivo* as described previously [18]. Briefly, mice that received saline or AAV8.*Ucn2* (2x10^13^gc/kg) were injected with 20 μg/g body weight of puromycin (Sigma) via intraperitoneal injection 7 weeks after gene transfer. Tissues were collected at 30 min after injection and immediately frozen in liquid nitrogen. Tissue homogenates were prepared in lysis buffer (1% SDS, 1% NP-40, 50 mM NaF, 2 mM EDTA, 1 mM Na_3_VO_4_, protease inhibitor cocktail tablet, 100 mM NaCl and 10 mM Tris-HCl, pH 7.5), and 50 μg total proteins were separated via SDS-PAGE. Immunoblotting was performed using anti-puromycin antibody (1:10000, EMD Millipore) and anti-vinculin antibody (1:100000, Sigma).

### Statistical analysis

Principal component analysis (PCA) were performed by Metabolon Inc. Briefly, the first principal component was computed by determining the coefficients of the metabolites that maximizes the variance of the linear combination. The second component finds the coefficients that maximize the variance with the condition that the second component is orthogonal to the first. The third component is orthogonal to the first two components and so on. The total variance was defined as the sum of the variance of the predicted values of each component (the variance is the square of the standard deviation), and for each component, the proportion of the total variance was computed.

Welch’s t-test was used for the two-group comparison, and null hypothesis was rejected when p < .05. Two-Way ANOVA was used to determine the interaction between diet and treatment. One-Way ANOVA was used for the three-group comparison and post hoc comparison was performed using Sidak’s multiple comparison test with correction.

### Selection criteria

Metabolites that were significantly altered by HFD or AAV gene transfer were selected from between group comparisons with value larger or smaller than 1 and p < .05. Up-regulated metabolites were highlighted in red and downregulated in green.

## Results

### Principal component analysis (PCA)

In the present study, 714 metabolites in liver were identified and categorized based on metabolic pathways (**Tables 1 and 2, S1 Table**). In order to allow visual assessment of similarities and differences between samples, PCA analysis were performed, where liver samples showed distinct separations between normal Chow (CHOW) and high fat diet (HFD) groups (**Fig 1B**). Although there are some overlaps within the same diet regimen, each treatment (saline, AAV8.Empt andAAV8.*Ucn2*) showed distinguishable separations indicating strong treatment effect (**Fig 1B**).

**Table 2:** Altered liver metabolites in mice fed with CHOW or HFD.

Super Pathway	AAV8.Ucn2 vs Saline in CHOW		AAV8.Ucn2 vs Saline in HFD		HFD vs CHOW		AAV8.Empt vs Saline in HFD	
	up	down	up	down	up	down	up	down
Amino acids	44	5	20	6	15	80	10	45
peptide	5	0	13	1	1	14	1	4
carbohydrate	5	15	10	1	0	18	15	2
Energy	3	0	1	0	1	2	0	0
lipid	67	25	43	98	77	149	3	91
nucleotide	22	2	18	7	8	36	10	20
cofactor & vitamins	12	1	14	2	9	20	6	1
Xenobiotics	3	3	5	0	1	17	0	1
**Total**	**161**	**51**	**124**	**115**	**112**	**336**	**45**	**164**

### AAV8.*Ucn2* regulates glucose homeostats in mice fed with normal chow

**Fig 2:**
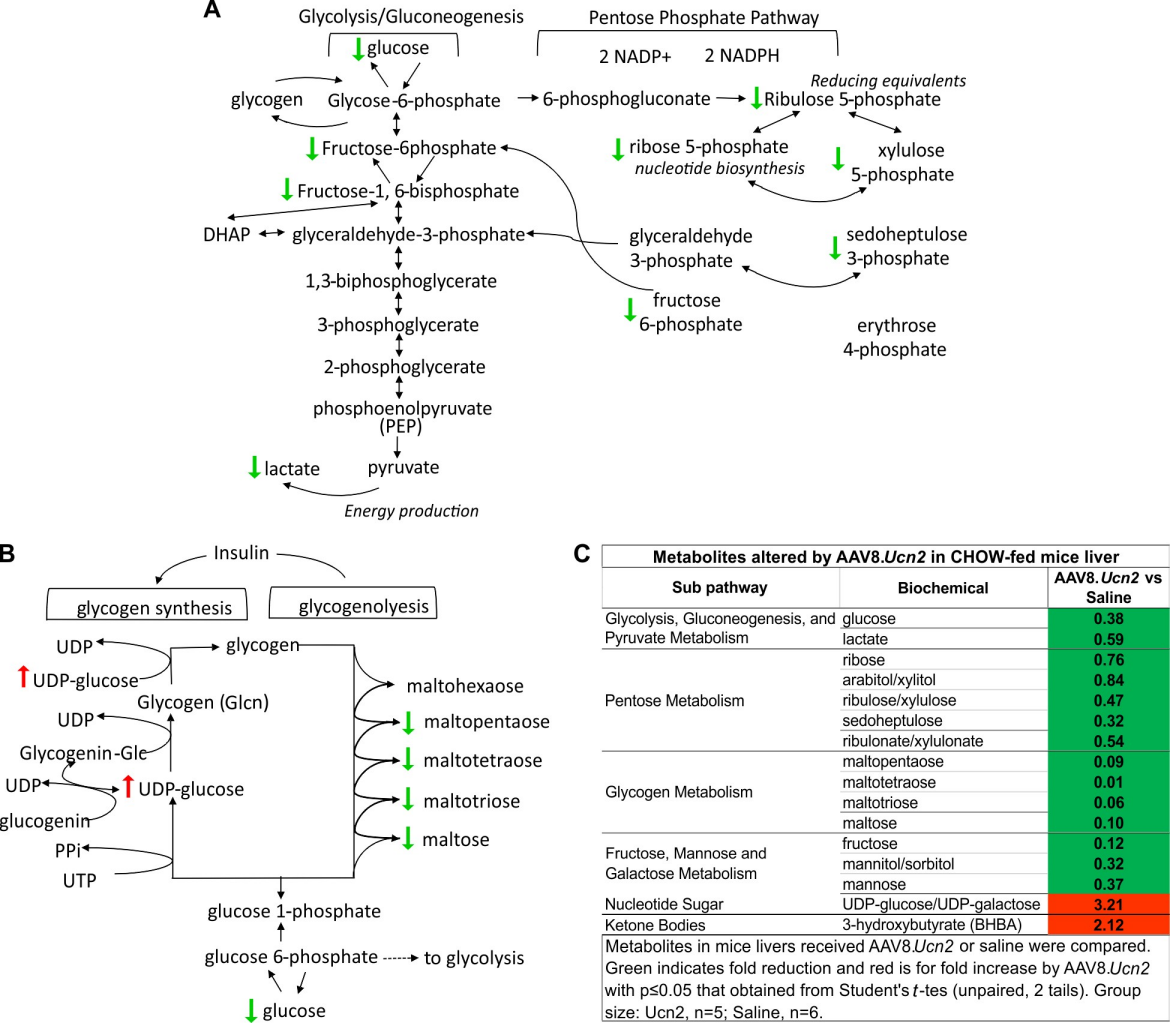
**A. Diagram of glycolysis/gluconeogenesis and pentose phosphate pathways.** Arrows indicate altered metabolites by AAV8.*Ucn2* gene transfer. **B. Diagram of glycogen synthesis and glycogenolysis pathways.** Arrows indicate altered metabolites by AAV8.*Ucn2* gene transfer. **C. Metabolites altered by AAV8.*Ucn2* in CHOW-fed mic liver.** Metabolites in mice livers received AAV8.*Ucn2* or saline were compared. Green indicates fold reduction and red is for fold increase by AAV8.*Ucn2* with p≤0.05 that were obtained from Student's t-tes (unpaired, 2 tails). Group size: Saline, n = 6; *Ucn2*, n = 5.

AAV8.*Ucn2* effects were determined by comparing metabolites altered by AAV8.*Ucn2* vs saline in CHOW-fed mice. As a result, AAV8.*Ucn2* altered 212 metabolites, in which 161 metabolites were upregulated and 51 were downregulated (**Fig 1C, Table 2, S1 and S2 Tables**). We have previously showed that AAV8.*Ucn2* gene transfer increases glucose disposal and decreases fasting glucose without hypoglycemia in normal C57BL/6 mice [14, 15]. Indeed, AAV8.*Ucn2* gene transfer reduced metabolites of glucose, lactate, 6-phosphogluconate (6pgn), sedoheptulose 7-phosphate (Sh7p) and pentose which indicate significant changes in glycolysis and pentose metabolisms (**Fig 2A and 2C)**. AAV8.*Ucn2* gene transfer also decreased polysaccharides such as maltopentaose, maltotetraose, maltotriose whereas it increased UDP-glucose/UDP-galactose in liver (**Fig 2B and 2C**). In addition, AAV8.*Ucn2* also increased ketone body by 2 fold (3-hydroxybutyrate, **Fig 2C**). Increased ketone bodies and decreased polysaccharides (glycogen) imply increased gluconeogenesis in hepatocytes due to low blood glucose level [19]. Therefore, it is plausible that gluconeogenesis in hepatocytes are increased to prevent hypoglycemia in normal mice that received *Ucn2* gene transfer. Taken together, AAV8.*Ucn2* gene transfer regulates glucose homeostats at multiple stages in glucose metabolism pathways.

### AAV8.*Ucn2* gene transfer reduced protein synthesis

**Fig 3:**
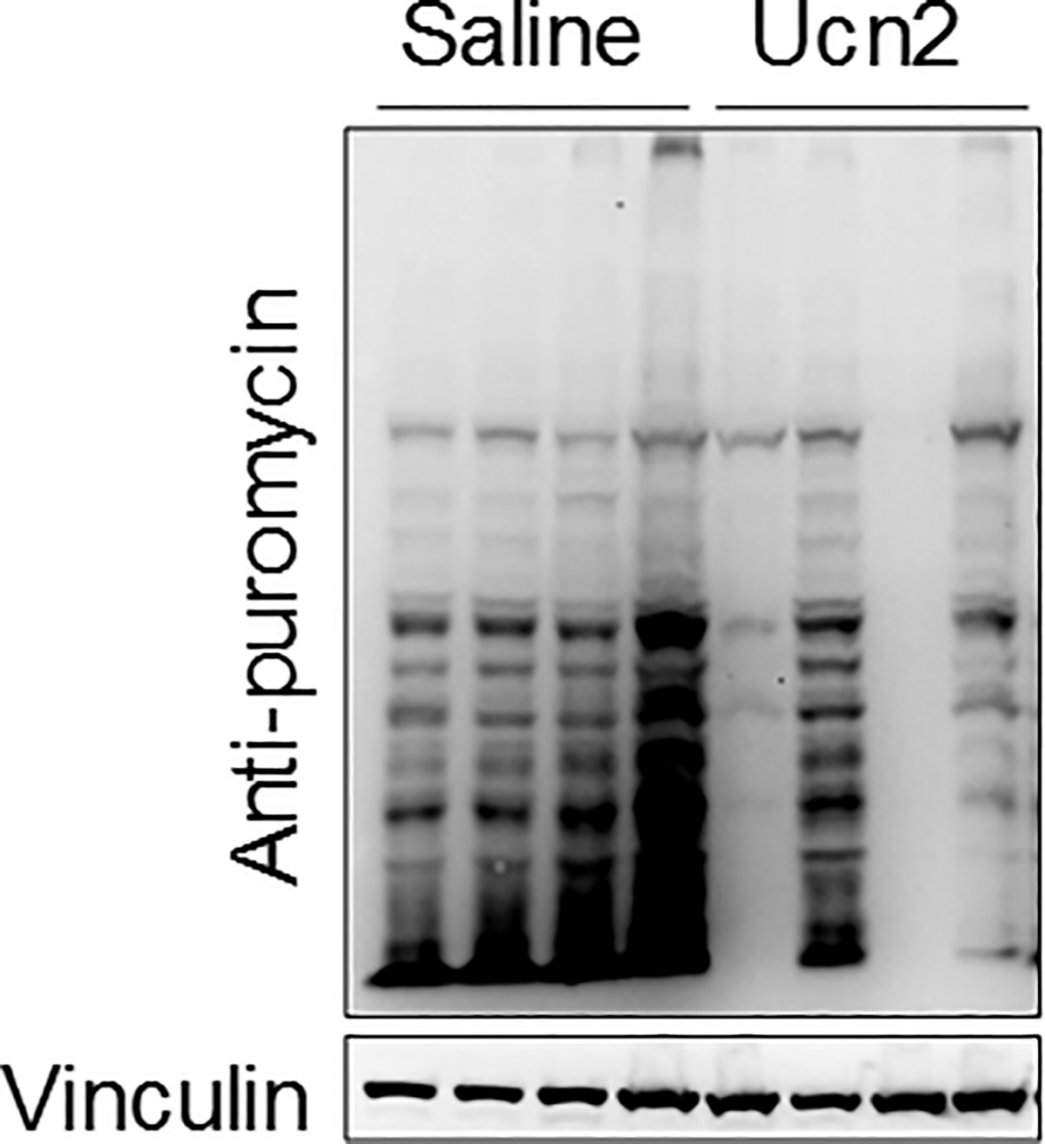
Protein synthesis reduced by AAV8.*Ucn2* gene transfer in CHOW. Immunoblotting of puromycin-incorporated newly synthesized proteins in liver. Liver protein synthesis was decreased in mice that received *Ucn2* gene transfer.

AAV8.*Ucn2* gene transfer increased 44 metabolites within amino acid pathways in CHOW-fed mice when compared to saline injected mice (**Tables 2 and 3, S1 and S2 Tables**). Increased levels of liver amino acids and intermediate metabolites might be resulted from reduced protein synthesis. To test this hypothesis, we examined protein synthesis in livers of normal mice, with and without AAV8.*Ucn2* gene transfer, using SUnSET maltopentaose method [20]. Incorporated puromycin in newly synthesized polypeptides was decreased in samples from mice that received AAV8.*Ucn2* vs mice that received saline (**Fig 3**). These results demonstrated that AAV8.*Ucn2* gene transfer reduces liver protein synthesis, which leads to increased liver metabolites in amino acid pathways.

**Table 3:** Metabolites increased by AAV8.Ucn2 in amino acid metabolisms in SHOW–fed mice. Liver metabolites were compared between AAV8.Ucn2 and saline (n = 5 per group). Red indicates fold increase by AAV8.Ucn2 with p≤.05 and metabolite ratio of <1.00.

Sub Pathway	Biochemical Name	AAV8.*Ucn2* vs Saline	Sub Pathway	Biochemical Name	AAV8.*Ucn2* vs Saline
Glycine, Serine and Threonine Metabolism	glycine	**1.24**	Leucine, Isoleucine and Valine Metabolism	3-methylcrotonylglycine	**2.78**
	N-acetylglycine	**1.78**		isoleucine	**1.35**
	betaine	**2.19**		N-acetylisoleucine	**1.34**
	betaine aldehyde	**2.40**		2-methylbutyrylglycine	**2.33**
	serine	**1.15**		valine	**1.32**
	N-acetylserine	**1.63**		N-acetylvaline	**1.57**
	threonine	**1.24**	Methionine, Cysteine, SAM and Taurine Metabolism	methionine	**1.31**
	N-acetylthreonine	**2.37**		N-acetylmethionine	**1.25**
Alanine and Aspartate Metabolism	aspartate	**1.47**		S-methylcysteine	**1.63**
	N-acetylaspartate (NAA)	**1.71**		hypotaurine	**2.55**
Histidine Metabolism	histidine	**2.08**		taurocyamine	**1.59**
	formiminoglutamate	**5.48**	Urea cycle; Arginine and Proline Metabolism	urea	**1.60**
	anserine	**1.79**		ornithine	**1.19**
Lysine Metabolism	lysine	**1.19**		proline	**1.26**
	N2-acetyllysine	**2.06**		dimethylarginine (SDMA + ADMA)	**1.33**
	N6-acetyllysine	**2.03**		N-acetylcitrulline	**2.52**
	N6,N6,N6-trimethyllysine	**1.50**		N-delta-acetylornithine	**4.51**
Phenylalanine Metabolism	phenylalanine	**1.18**		N-alpha-acetylornithine	**1.98**
Tryptophan Metabolism	C-glycosyltryptophan	**1.49**		N-monomethylarginine	**1.21**
	kynurenine	**3.74**	Guanidino and Acetamido Metabolism	4-guanidinobutanoate	**1.63**
Leucine, Isoleucine and Valine Metabolism	leucine	**1.20**	Glutathione Metabolism	5-oxoproline	**1.28**
	isovalerylglycine	**1.66**		2-hydroxybutyrate/2-hydroxyisobutyrate	**2.10**

### HFD effects on liver metabolites (HFD vs CHOW)

To determine the effects of HFD on mice liver metabolism, we compared metabolites between saline injected HFD-fed and CHOW-fed mice. HFD altered 63% (448 out of 714) of alldetected liver metabolites, in which 112 were upregulated and 336 were downregulated (**Fig 1C, Table 2, S1 and S3 Tables**). Fifty percent of metabolites altered by HFD are within the lipid metabolism pathway (77 upregulated and 149 downregulated, **Table 2**). HFD results in significant increase in long chain fatty acids (8/15), polyunsaturated fatty acids (6/18), acyl carnitine (6/15), diacylglycerols (17/39), ceramide (10/13) and sphingosines (3/3), metabolites most likely associated with insulin resistance and fatty liver [21]. On the other hand, HFD reduced 47 out of 56 metabolites within phospholipid metabolism pathways including phosphatidylcholine (PC), phosphatidylethanolamine (PE), phosphatidylserine (PS), phosphatidylglycerol (PG), phosphatidylinositol (PI), metabolites required for normal cell metabolisms [22, 23]. HFD also resulted in significant reduction of lysophospholipids (12/26) and plasmalogens (5/8) (**S1 and S3 Tables**).

HFD also down regulated 36 metabolites within nucleotide metabolism and 80 amino acids (**Table 2, S1 and S3 Tables**), which could be linked to increased DNA and protein synthesis, effects that opposite to what we have seen from AAV8.*Ucn2* gene transfer (**Table 2 and S2 Table**).

### Interaction between diets and AAV8.*Ucn2* gene transfer

The effects of AAV8.*Ucn2* in HFD vs normal chow were analyzed using two-way ANOVA on four groups of samples (Chow-saline, HFD-saline, Chow-Ucn2, and HFD-Ucn2). Sixty-one metabolites showed interactions between diet and treatment. Among these 61 metabolite, 13 of them in 4 super pathways showed that treatment effects, meaning that HFD changed them, but AAV8.*Ucn2* restored them to the normal levels seen in Chow-fed mice. (Table 4 and S4 Table). Partially, HFD increased levels of 3 diacylglycerols in lipid super pathway, but AAV8.*Ucn2* reduced them. These findings suggest that AAV8.*Ucn2* gene transfer increases insulin sensitivity by reducing liver diacylglycerols.

**Table 4:** Interaction between diet and treatment. Liver metabolites from 4 groups (CHOW-Saline, HFD-Saline, CHOW-Ucn2, and HFD-Ucn2) of mice were analyzed using 2-Way ANOVA. Sixth-one out of 714 metabolites had significant interaction between diet and AAV8.Ucn2 treatment. The rows containing bold and italic numbers showed that AAV8.Ucn2 reversed HFD effects. CHOW-Saline, n = 6; CHOW-Ucn2, n = 5; HFD-Saline and HFD-Ucn2, n = 8/group.

	Metabolites	Mean				*p*-value		
		Chow-Saline	HFD-Saline	Chow-*Ucn2*	HFD-*Ucn2*	Treatment	Diet	Interactions
1	N-acetylserine	1.5321	1.0792	2.5041	1.0167	0.0049	<0.0001	0.0007
2	threonine	1.6180	1.0125	2.0012	1.0079	0.0189	<0.0001	0.0115
3	N-acetylthreonine	1.5807	0.9403	3.7505	1.0949	<0.0001	<0.0001	0.0002
4	aspartate	1.6503	1.0055	2.4269	1.0707	0.0014	<0.0001	0.0170
5	histidine	1.3640	1.0165	2.8428	1.0668	<0.0001	<0.0001	<0.0001
6	formiminoglutamate	4.3343	1.0623	23.7655	0.8382	0.0208	<0.0001	0.0020
7	N6-acetyllysine	2.0371	0.9660	4.1365	1.1449	0.0044	<0.0001	0.0161
8	N6,N6,N6-trimethyllysine	1.4315	1.0021	2.1485	1.0281	0.0108	<0.0001	0.0167
9	phenylalanine	1.5064	1.0180	1.7716	1.0433	0.0022	<0.0001	0.0191
10	kynurenine	1.9366	0.8673	7.2380	1.1672	0.0001	<0.0001	0.0044
11	leucine	1.4775	1.0288	1.7659	1.0299	0.0222	<0.0001	0.0165
12	isoleucine	1.3664	1.0416	1.8392	1.0274	0.0009	<0.0001	0.0004
13	2-methylbutyrylglycine	1.5394	1.0372	3.5843	1.2489	0.0064	<0.0001	0.0303
14	valine	1.4931	1.0175	1.9729	1.0276	0.0014	<0.0001	0.0018
15	N-acetylvaline	1.6569	0.9696	2.5977	1.0075	0.0052	<0.0001	0.0244
16	isobutyrylcarnitine (C4)	***2*.*9096***	***0*.*5151***	***3*.*2379***	***0*.*9041***	***0*.*0072***	***<0*.*0001***	***0*.*0430***
17	methionine	1.5555	1.0051	2.0435	1.0741	0.0004	<0.0001	0.0178
18	S-adenosylhomocysteine (SAH)	***1*.*4427***	***0*.*4942***	***1*.*6648***	***0*.*9419***	***0*.*0002***	***<0*.*0001***	***0*.*0117***
19	hypotaurine	1.5448	0.7695	3.9403	0.9219	0.0026	<0.0001	0.0140
20	taurine	0.9698	1.0192	0.5331	1.0292	0.0001	<0.0001	0.0001
21	ornithine	1.2841	1.0516	1.5298	0.9967	0.0461	<0.0001	0.0006
22	homocitrulline	***1*.*9965***	***0*.*2370***	***1*.*7740***	***0*.*9266***	***0*.*0042***	***<0*.*0001***	***0*.*0016***
23	proline	1.4484	1.0207	1.8256	1.0068	0.0381	<0.0001	0.0165
24	N-delta-acetylornithine	0.8445	0.6081	3.8076	0.6667	<0.0001	<0.0001	0.0000
25	putrescine	***0*.*1603***	***2*.*3322***	***0*.*1454***	***1*.*0941***	***0*.*0023***	***<0*.*0001***	***0*.*0092***
26	5-oxoproline	1.0353	0.9864	1.3281	1.0322	0.0052	0.0036	0.0419
27	2-hydroxybutyrate/2-hydroxyisobutyrate	0.9883	1.0638	2.0779	1.1732	0.0008	0.0320	0.0040
28	glucose	1.2548	0.8923	0.4819	1.0034	0.0003	0.0276	<0.0001
29	lactate	1.2071	1.1515	0.7062	1.0778	0.0005	0.0132	0.0040
30	ribitol	***0*.*9097***	***0*.*4767***	***1*.*0918***	***0*.*9564***	***0*.*0002***	***0*.*0004***	***0*.*0210***
31	maltotetraose	***0*.*9153***	***0*.*5984***	***0*.*0109***	***1*.*1349***	***0*.*0010***	***0*.*0018***	***0*.*0012***
32	maltotriose	***0*.*9439***	***0*.*6357***	***0*.*0597***	***0*.*9052***	***0*.*0004***	***0*.*0028***	***0*.*0003***
33	maltose	1.4385	0.9168	0.1446	0.9500	<0.0001	0.0003	<0.0001
34	N-acetylglucosamine/N-acetylgalactosamine	1.6037	1.1199	2.9727	1.0553	0.0200	<0.0001	0.0024
35	N6-carboxymethyllysine	0.9251	0.1324	1.2886	0.1324	0.0114	<0.0001	0.0114
36	5-dodecenoate (12:1n7)	1.8617	1.0700	3.0506	0.9741	0.0332	<0.0001	0.0041
37	hexadecanedioate (C16-DC)	1.1552	0.9881	2.3194	1.0739	0.0043	0.0013	0.0322
38	valerylglycine	***1*.*6318***	***1*.*0654***	***4*.*2588***	***1*.*5946***	***0*.*0023***	***0*.*0012***	***0*.*0391***
39	palmitoylcholine	0.8010	0.7888	3.6191	1.2326	0.0001	0.0221	0.0348
40	arachidonoylcholine	0.4098	0.3820	3.5247	0.6303	0.0007	0.0175	0.0288
41	1,2-dipalmitoyl-GPC (16:0/16:0)	1.2275	1.0870	1.5420	1.0206	0.0090	<0.0001	0.0000
42	1-oleoyl-2-arachidonoyl-GPI (18:1/20:4)*	1.4205	0.9821	1.8321	1.0031	0.0135	<0.0001	0.0194
43	1-stearoylglycerol (18:0)	***0*.*6034***	***1*.*8467***	***0*.*5819***	***1*.*1951***	***0*.*0063***	***<0*.*0001***	***0*.*0192***
44	diacylglycerol (12:0/18:1, 14:0/16:1, 16:0/14:1) [1]*	***0*.*2473***	***1*.*9899***	***0*.*1775***	***0*.*6464***	***0*.*0107***	***<0*.*0001***	***0*.*0363***
45	palmitoyl-palmitoyl-glycerol (16:0/16:0) [1]*	***0*.*5136***	***1*.*5408***	***0*.*5020***	***1*.*0977***	***0*.*0411***	***<0*.*0001***	***0*.*0307***
46	oleoyl-arachidonoyl-glycerol (18:1/20:4) [1]*	***0*.*1894***	***1*.*3737***	***0*.*6674***	***1*.*0337***	***0*.*0018***	***<0*.*0001***	***<0*.*0001***
47	sphinganine	0.5365	1.2512	1.1221	1.1474	0.0021	<0.0001	0.0002
48	ceramide (d18:2/24:1, d18:1/24:2)*	1.3395	0.9986	1.7283	1.0045	0.0269	<0.0001	0.0350
49	glycosyl-N-palmitoyl-sphingosine (d18:1/16:0)	1.0640	1.0310	1.5994	0.9748	0.0273	0.0015	0.0052
50	glycosyl ceramide (d18:2/24:1, d18:1/24:2)*	1.6794	1.0506	3.0063	1.0466	0.0017	<0.0001	0.0021
51	beta-sitosterol	0.7925	0.3923	1.1884	0.3923	0.0080	<0.0001	0.0080
52	xanthine	1.0628	0.9850	1.2964	1.0200	0.0002	<0.0001	0.0038
53	uric acid ribonucleoside*	0.3748	1.4953	1.6306	2.0843	0.0003	<0.0001	0.0031
54	N1-methyladenosine	1.0651	0.9312	2.0178	1.0551	0.0122	0.0036	0.0487
55	pseudouridine	1.3085	1.0482	2.7075	0.9582	0.0140	<0.0001	0.0017
56	3-ureidopropionate	***0*.*0120***	***4*.*2368***	***0*.*0115***	***0*.*7167***	***0*.*0012***	***<0*.*0001***	***0*.*0008***
57	(3'-5')-uridylyluridine	***1*.*9816***	***0*.*3975***	***1*.*3716***	***1*.*1392***	***0*.*0258***	***<0*.*0001***	***0*.*0001***
58	cinnamoylglycine	0.7188	0.2628	1.1825	0.2628	0.0244	<0.0001	0.0244
59	enterolactone	3.9272	0.2281	1.0208	0.2281	0.0111	<0.0001	0.0111
60	methyl glucopyranoside (alpha + beta)	1.9493	0.8953	0.7890	1.1113	0.0002	0.0334	<0.0001
61	3-hydroxypyridine sulfate	1.3336	0.2168	1.8147	0.2168	0.0163	<0.0001	0.0163

### Effects of AAV8.Empt vector on liver metabolites in insulin resistant mice

The effects from AAV8.Empt vector gene transfer were determined by comparison of metabolites from HFD-fed mice that received AAV8.Empt vs those that received saline. AAV8.Empt vector altered 209 metabolites that were widely distributed within 6 super pathways including amino acid, peptide, carbohydrate, lipid metabolites, nucleotides, and cofactors/vitamins (**Fig 1C, Table 2, S1 and S5 Tables**). AAV8.Empt gene transfer did not alter glucose disposal and insulin resistance [15]. Therefore, the vector effects, along with the effects from saline injection, served as controls in determination of the effects solely from *Ucn2* gene expression.

### Effects of AAV8.*Ucn2* gene transfer on live in insulin resistant mice

The effects of AAV8.*Ucn2* gene transfer, which include the effects from AAV vector and *Ucn2* expression, were determined by comparing metabolites altered by AAV8.*Ucn2* vs saline in HFD-fed mice liver. AAV8.*Ucn2* gene transfer altered 239 metabolites in which 124 were upregulated and 115 were downregulated (**Fig 1C, Table 2, S1 and S6 Tables**).

### Different effects of AAV8.*Ucn2* vs AAV8.Empt on liver in insulin resistant mice

Next, we determined metabolites that were differently altered by gene transfer of AAV8.*Ucn2* vs AAV8.Empt. Metabolites in multiple pathways were differently changed by AAV8.*Ucn2* (**Table 2 and S7 Tables**). For example, **a)** within amino acids, AAV8.*Ucn2* increased 20, but AAV8.Empt decreased 45 detected biochemicals (**Table 2 and S7-1 Table**). **b)** Within peptide metabolites, AAV8.*Ucn2* increased 13, but AAV8.Empt decreased 4 biochemicals (**Table 2 and S7-2 Table**). **c)** Within nucleotide super pathways, AAV8.*Ucn2* increased 18 and decreased 7 metabolites while AAV8.Empt increased 10 but decreased 20 metabolites (**Table 2 and S7-3 Table**). **d)** In cofactor/vitamins super pathways, AAV8.*Ucn2* increased 14, but AAV8.Empt increased 6 metabolites (**Table 2 and S7-4 Table**). **e)** In fatty acid metabolism sub pathways, AAV8.*Ucn2* increased 11, but AAV8.Empt did not alter any metabolites (**S7-5 Table**). **f)** In diacylglycerols sub pathways, AAV8.*Ucn2* reduced 17, but AAV8.Empt reduced 5 metabolites (**S7-6 Table**). **g)** In phospholipid sub pathways, AAV8.*Ucn2* increased 15, but AAV8.Empt increased 1 metabolite (**S7-7 Table**). Finally, AAV8.*Ucn2* increased folate metabolism [5-methyltetrahydrofolate (5MeTHF)] by 1.9-fold, but no effect from AAV8.Empt (**S6 Table**). We speculate that these different effects of AAV8.*Ucn2* play important roles in increased glucose disposal and insulin sensitivity seen in HFD mice that received AAV8.*Ucn2*.

### Effects of increased plasma Ucn2 on liver in insulin resistant mice

**Fig 4:**
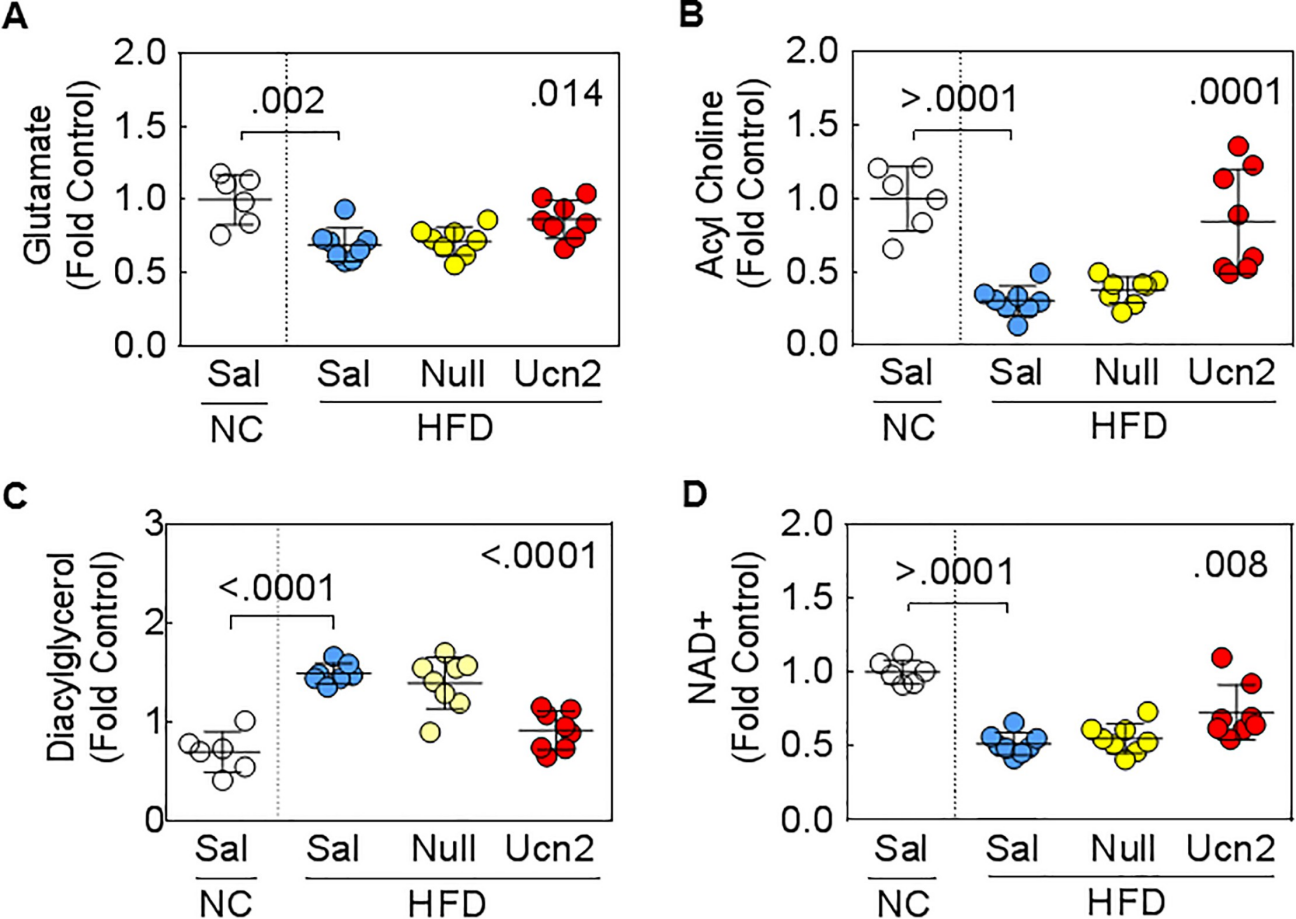
Examples of metabolites restored by *Ucn2* expression in mice fed with HFD. **A.** Glutamate was reduced by HFD and restored close to normal by Ucn2. **B.** Choline was reduced by HFD and increased close to normal by Ucn2. **C.** Diacylglycerol was increased by HFD but reduced by Ucn2. **D.** Nicotinamide adenine dinucleotide (NAD+) was reduced by HFD but increased by *Ucn2* expression. SID: ScaledImpData, where each biochemical in original scale is rescaled to set the median equal to 1. Sal, saline; Empt, AAV8.Empt; Ucn2, AAV8.*Ucn2*. Data are mean+SEM; p values on left side in the graph were from Student’s *t*-test and on the right were from one-way ANOVA. Group size: CHOW-saline, n = 6; HFD, n = 8 per group.

Previous studies showed that AAV8.*Ucn2* gene transfer increased blood circulating Ucn2 peptide >15-fold [11]. To determine the effects of plasma Ucn2, metabolites from HFD-fed mice received AAV8.*Ucn2* were compared against that received saline and AAV8.Empt. Metabolite levels in saline injected CHOW-fed mice were used as control. Eighteen metabolites were identified using one-way ANOVA. Importantly, plasma Ucn2 restored the levels of these hepatic metabolites to that seen in CHOW-fed mice (**Table 5**). Specifically, **a)** Ucn2 restored 4 amino acids and 2 dipeptides towards normal. For example, HFD downregulated levels of glutamate by 47%, but Ucn2 brought it back to normal levels (CHOW-saline, 1.27±0.09; HFD-saline, 0.88 ±0.05; HFD-Ucn2, 1.1±0.06. p = .01. **Table 5, Fig 4A**). **b)** Ucn2 restored metabolites in fatty acid metabolisms. For example, linoleoylcholine was reduced 3-fold by HFD, but Ucn2 restored it towards normal (CHOW-saline, 1.52±0.14; HFD-saline 0.46 ±0.05; HFD-Ucn2, 1.3±0.2, p = .0001. **Table 5, Fig 4B**). **c)** HFD increased 3 metabolites in diacylglycerols family, but Ucn2 reduced them towards normal. For example, HFD increased diacylglycerols by 2.3-fold, but Ucn2 reduced it towards normal (CHOW-saline, 0.7±0.08; HFD-saline, 1.6 ±0.3; HFD-Ucn2, 0.9±0.16, p < .0001. **Table 5, Fig 4C**). **d)** In nucleotide metabolisms, Ucn2 expression normalized five metabolites that were reduced by HFD (**Table 5**). **e)** HFD decreased the level of nicotinamide adenine dinucleotide NAD+, a common phenomenon in HFD fed rodents [24], but Ucn2 increased it towards normal (CHOW-saline, 1.4±0.05; HFD-saline 0.73 ±0.05; HFD-Ucn2, 1.02, **Table 5 and Fig 4D**).

**Table 5:** Metabolites restored by Ucn2 expression in HFD-fed mice. Identified metabolites are presented using scaled imput data (*SID) where each biochemical in original scale is rescaled to set the median to 1. Metabolites in saline-injected Chow-fed mice were used as controls. Green indicates downregulation and red for upregulation when compared with that in Chow-fed saline controls. The colors in HFD-fed Ucn2 column indicate restored metabolites when compared with that in HFD saline group. Data from mean ± SEM. Group size: Chow-fed, n = 6; HFD-fed, n = 8 in each group. P values are from 1-way ANOVA analysis of 3 HFD-fed groups.

	Sub Pathway	Metabolites	Raw data (*SID)				*p value*
			CHOW—fed	HFD—fed			
			Saline (6)	Saline (8)	Empt (8)	*Ucn2* (8)	*1-w ANOVA*
1	Glutamate Metabolism	gamma-carboxyglutamate	1.27±0.09	0.88±0.05	0.91±0.04	1.10±0.06	*0*.*0100*
2		glutamate, gamma-methyl ester	1.52±0.17	0.81±0.08	0.81±0.11	1.21±0.11	*0*.*0118*
3	Histidine Metabolism	1-methylhistidine	1.48±0.15	0.98±0.11	0.88±0.07	1.34±0.16	*0*.*0280*
4	Tryptophan Metabolism	C-glycosyltryptophan	1.50±0.11	0.90±0.06	0.97±0.03	1.24±0.12	*0*.*0176*
5	Dipeptide	glycylisoleucine	1.97±0.17	1.08±0.14	0.88±0.11	1.69±0.27	*0*.*0137*
6		isoleucylglycine	1.58±0.18	0.86±0.11	0.95±0.14	1.54±0.23	*0*.*0214*
7	Fatty Acid, Dicarboxylate	3-methylglutarate/2-methylglutarate	1.42±0.12	0.86±0.13	0.77±0.09	1.35±0.10	*0*.*0019*
8	Fatty Acid Metabolism (Acyl Choline)	linoleoylcholine	1.52±0.14	0.46±0.05	0.57±0.05	1.29±0.19	*0*.*0001*
9	Fatty Acid, Dihydroxy	12,13-DiHOME	1.48±0.16	0.91±0.07	0.75±0.05	1.25±0.14	*0*.*0042*
10	Diacylglycerol	diacylglycerol	0.69±0.08	1.58±0.27	1.39±0.22	0.91±0.16	*<0*,*0001*
11		palmitoleoyl-palmitoleoyl-glycerol	0.86±0.08	1.59±0.10	1.33±0.09	0.91±0.06	*<0*,*0001*
12		palmitoleoyl-oleoyl-glycerol	0.81±0.08	1.42±0.07	1.23±0.09	0.91±0.06	*0*.*0013*
13	Purine Metabolism, (Hypo)Xanthine/Inosine	xanthine	1.06±0.08	0.99±0.07	0.94±0.10	1.02±0.08	*0*.*0395*
14		xanthosine	1.43±0.02	0.99±0.02	0.60±0.01	1.16±0.03	*0*.*0142*
15	Purine Metabolism, Adenine containing	N6-carbamoylthreonyladenosine	1.49±0.19	0.87±0.10	0.75±0.06	1.15±0.19	*0*.*0310*
16	Pyrimidine Metabolism, Uracil containing	uridine-2',3'-cyclic monophosphate	1.66±0.10	0.89±0.08	0.70±0.05	1.38±0.15	*0*.*0188*
17		2'-O-methyluridine	0.99±0.21	0.62±0.14	0.75±0.11	1.01±0.22	*0*.*0066*
18	Nicotinate and Nicotinamide Metabolism	nicotinamide adenine dinucleotide (NAD+)	1.40±0.05	0.72±0.05	0.77±0.05	1.02±0.12	*<0*,*0001*

## Discussion

AAV8.*Ucn2* gene transfer was associated with increased insulin sensitivity, reduced hyperglycemia and fatty liver in mice that developed insulin resistance [15]. However, the liver metabolic changes in mice that received AAV8.*Ucn2* is unknown. We performed untargeted metabolomic study to determine the effects of AAV8.*Ucn2* in normal chow-fed and HFD-fed mice and identified 61 metabolites that had significant interaction between diet and AAV8.*Ucn2* treatment (**Table 4 and S4 Table**). We also determined the effects from increased *Ucn2* expression (blood circulating Ucn2) on liver metabolisms in mice rendered insulin resistant by HFD and identified 18 metabolites that are altered by HFD, but restored by plasma Ucn2 to the levels seen in CHOW-fed mice livers (**Fig 4 and Table 5**).

### Regulation of glucose homeostasis

AAV8.*Ucn2* gene transfer in CHOW-fed mice was associated with increased glycolysis, pentose metabolisms and glycogenolysis (**Fig 2; Tables 2 and 3**), which are the underlying mechanisms for reduced blood glucose. AAV8.*Ucn2* gene transfer reduced blood glucose level by 24%, but it did not induce hypoglycemia [15], which suggests that AAV8.*Ucn2* was able to regulate glucose homeostasis. Indeed, AAV8.*Ucn2* gene transfer resulted in increased levels of ketone body (3-hydroxybutyrate) and UDP-glucose/UDP-galactose (**Fig 2**). Increasing ketone body and decreasing mono- and oligo-saccharides imply increased gluconeogenesis in hepatocytes due to low blood glucose level [19]. Therefore, it is plausible that gluconeogenesis in hepatocytes are increased to prevent hypoglycemia in normal mice that received AAV8.*Ucn2*. The UDP-glucose/UDP-galactose serves as building blocks for glycogen synthesis. Therefore, the observed alterations suggest increased glycogen synthesis when blood glucose level is high or stalled glycogen synthesis when glucose become low. Unexpected, reduced glucose and polysaccharides or increased ketone bodies and UDP-glucose/UDP-galactose, were not detected in HFD-fed mice that received AAV8.*Ucn2* (**S1 and S3 Tables**) despite the fact that AAV8.*Ucn2* increased glucose disposal as seen in HFD-fed mice [15].

### Amino acid and peptide metabolisms

HFD reduced 80 metabolites within amino acids and peptide metabolism (**Table 2 and S3 Table**), which could be associated with increased protein synthesis. In contrast, AAV8.*Ucn2* gene transfer increased these metabolites in CHOW-fed (**Table 2 and S2 Table**) and HFD-fed mice (**Table 2, S6, S7-1 and S7-2 Tables**), which could be associated with reduced protein synthesis seen in CHOW-fed mice livers (**Fig 3**). Reduced protein synthesis correlated well with reduced body weight and increased insulin sensitivity in HFD-fed mice received AAV8.*Ucn2* gene transfer [15]. The molecular mechanism of reduced protein synthesis by AAV8.*Ucn2* is not fully understood. Since mTORC1 (mechanistic target of rapamycin complex 1) signaling negatively regulates protein synthesis via activation of 5’ adenosine monophosphate-activated protein kinase (AMPK) and Ucn2 activates AMPK [15, 25, 26], it is likely that UCn2 reduces protein synthesis through activation of AMPK/mTORC signaling pathways.

Plasma Ucn2 selectively restored 4 amino acids (gamma-carboxyglutamate, glutamate, gamma-methyl ester, 1-methylhistidine, C-glycosyltryptophan) and 2 dipeptides (glycylisoleucine and isoleucylglycine) to normal levels seen CHOW-fed mice (**Table 5**), which implies that these molecules play crucial roles in regulating glucose metabolisms. For example, glutamate metabolism plays essential role in promoting and maintaining function of various organs and cells. It serves as an important fuel for cells in the tricarboxylic acid (TCA) cycle [27]. Glutamate, besides providing fuel for cells, also stimulates insulin secretion [28]. Our results suggest that Ucn2 may be able to redirect amino acids from protein synthesis to energy production or insulin release.

### Lipid metabolisms

HFD increased levels of diacylglycerols, which are associated with increased insulin resistance [21]. AAV8.*Ucn2* gene transfer and plasma Ucn2 reduced diacylglycerols, which may contribute to increased insulin sensitivity and reduced fatty liver in HFD mice as reported in a previous study [15].

Plasma Ucn2 increased choline-folate (cofactor) metabolite, (i.e. betaine aldehyde and 5-methyltetrahydrofolate, **Table 5, S2 and S6 Tables**). Choline is an essential nutrient and facilitates fatty acid β-oxidation and liver function [29]. Folate mediates one-carbon transfer reaction that is important for amino acid and nucleotide biosynthesis and methylation reactions [30]. Increases in these pathways suggest that Ucn2 regulates hepatic fatty acid metabolism by facilitating choline uptake and amino acid and nucleotide production [31].

AAV8.*Ucn2* gene transfer also altered phospholipid metabolites in HFD-fed mice (**S7-7 Table).** Phospholipids are the main components of cell membrane and a second messenger for cellular signaling [22, 23]. They play important roles in fat absorption, lipoproteins transportation, and transduction of biological signals across membrane in association with the calcium flux [32]. They are also critical molecules in modulating ER stress, lipolysis, FA oxidation, energy expenditure, and the pathogenesis of obesity [33]. Therefore, an increase in phospholipids associated with Ucn2 gene transfer may have beneficial effects on liver. Taken together, reduce diacylglycerols and increased acyl carnitines, choline, and phospholipids are potentially important mechanisms for increased insulin sensitivity and reduced fatty liver in mice that received *Ucn2* gene transfer [15].

### Cofactors/Vitamins metabolisms

HFD-fed mice showed decreased hepatic nicotinamide adenine dinucleotide (NAD+) as previously reported [24]. It was noteworthy that plasma Ucn2 restored hepatic NAD+ to levels similar to those found in CHOW-fed mice (**Fig 4 and Table 5**). Metabolites in nicotinate and nicotinamide metabolism are essential for organisms as the precursors for the generation of coenzymes, NAD^+^ and NADP^+^, which are required for redox reactions and electron transport [34]. These coenzymes are crucial for many metabolic pathways including glycolysis, TCA cycle, pentose phosphate pathway, fatty acid biosynthesis and metabolisms. NAD+ serves as a substrate for sirtuins to regulate activity of signaling proteins by deacetylation [35]. Increased NAD+ levels are linked with improved mitochondrial function, liver regeneration and potential treatment of nonalcoholic fatty liver disease [36]. Therefore, restoration of NAD+ towards normal by Ucn2 may protect animal against dietary-related metabolic complications.

## Limitations

In our untargeted metabolomic assay, we used an extraction method targeted towards broad coverage rather than tailored towards a specific metabolism class. As a result, compounds that are in low abundance (or that are not extracted efficiently with the methanol-based method) will not be represented in the dataset. This is not necessarily a limitation, as changes in related biochemicals (precursors, products and co-factors) that are measured can imply changes are present in missing compounds. However, global analysis we are, of necessity, looking at a single point in time. More detailed flux studies can be used to confirm biochemical pathway changes, but are often not necessary when protein/transcript data can reveal changes observed. The time difference after gene delivery between CHOW and HFD groups introduced a potential confounding variable. However, our key findings were generated from AAV8.*Ucn2* against AAV8.Empt and saline that were all conducted in the HFD group (**Fig 4 and Table 4**). Finally, we studied only liver. We elected to do this to curtail the otherwise large data sets and the challenge of complex analysis. Metabolomic studies on skeletal muscle, pancreas, and plasma will be performed and analyzed in subsequent studies.

## Conclusions

*Ucn2* gene transfer altered metabolites in liver that are associated with glucose metabolism and insulin resistance in HFD mice—restoring key metabolites to normal levels. These findings provide potential mechanisms by which *Ucn2* gene transfer increases total body glucose disposal in HFD-associated insulin resistance.

## Supporting information

S1 Raw Image (PDF)

S1 Table Summary of effects on 714 liver metabolites. (PDF)

S2 Table AAV8.*UCn2* altered metabolites in CHOW-fed mice. (PDF)

S3 Table HFD altered metabolites in liver (HFD-saline vs CHOW-saline). (PDF)

S4 Table Effects of diet and AAV8.*Ucn2* on Chow-fed and HFD-fed mice liver metabolites. (PDF)

S5 Table AAV8.Empt altered metabolites in HFD-fed mice liver. (PDF)

S6 Table AAV8.*Ucn2* altered metabolites in HFD-fed mice liver. (PDF)

S7 Table Metabolites altered differently by AAV8.*Ucn2* vs AAV8.Empt in HFD-fed mice liver. (PDF)

## References

[1] Reyes TM, Lewis K, Perrin MH, Kunitake KS, Vaughan J, Arias CA, et al Urocortin II: a member of the corticotropin-releasing factor (CRF) neuropeptide family that is selectively bound by type 2 CRF receptors. Proc Natl Acad Sci U S A. 2001;98(5):2843–8. 10.1073/pnas.051626398 11226328 PMC30227

[2] Hsu SY, Hsueh AJ. Human stresscopin and stresscopin-related peptide are selective ligands for the type 2 corticotropin-releasing hormone receptor. Nat Med. 2001;7(5):605–11. 10.1038/87936 . 11329063

[3] Kishimoto T, Pearse RV 2nd, Lin CR, Rosenfeld MG. A sauvagine/corticotropin-releasing factor receptor expressed in heart and skeletal muscle. Proc Natl Acad Sci U S A. 1995;92(4):1108–12. 10.1073/pnas.92.4.1108 7755719 PMC42647

[4] Stenzel P, Kesterson R, Yeung W, Cone RD, Rittenberg MB, Stenzel-Poore MP. Identification of a novel murine receptor for corticotropin-releasing hormone expressed in the heart. Mol Endocrinol. 1995;9(5):637–45. 10.1210/mend.9.5.7565810 . 7565810

[5] Chen A, Perrin M, Brar B, Li C, Jamieson P, Digruccio M, et al Mouse corticotropin-releasing factor receptor type 2alpha gene: isolation, distribution, pharmacological characterization and regulation by stress and glucocorticoids. Mol Endocrinol. 2005;19(2):441–58. 10.1210/me.2004-0300 . 15514029

[6] Chen A, Blount A, Vaughan J, Brar B, Vale W. Urocortin II gene is highly expressed in mouse skin and skeletal muscle tissues: localization, basal expression in corticotropin-releasing factor receptor (CRFR) 1- and CRFR2-null mice, and regulation by glucocorticoids. Endocrinology. 2004;145(5):2445–57. 10.1210/en.2003-1570 . 14736736

[7] Perrin M, Donaldson C, Chen R, Blount A, Berggren T, Bilezikjian L, et al Identification of a second corticotropin-releasing factor receptor gene and characterization of a cDNA expressed in heart. Proc Natl Acad Sci U S A. 1995;92(7):2969–73. 10.1073/pnas.92.7.2969 7708757 PMC42340

[8] Richard D, Lin Q, Timofeeva E. The corticotropin-releasing factor family of peptides and CRF receptors: their roles in the regulation of energy balance. Eur J Pharmacol. 2002;440(2–3):189–97. 10.1016/s0014-2999(02)01428-0 . 12007535

[9] Hillhouse EW, Grammatopoulos DK. The molecular mechanisms underlying the regulation of the biological activity of corticotropin-releasing hormone receptors: implications for physiology and pathophysiology. Endocr Rev. 2006;27(3):260–86. 10.1210/er.2005-0034 . 16484629

[10] Paruthiyil S, Hagiwara SI, Kundassery K, Bhargava A. Sexually dimorphic metabolic responses mediated by CRF2 receptor during nutritional stress in mice. Biol Sex Differ. 2018;9(1):49 10.1186/s13293-018-0208-4 30400826 PMC6218963

[11] Gao MH, Lai NC, Miyanohara A, Schilling JM, Suarez J, Tang T, et al Intravenous adeno-associated virus serotype 8 encoding urocortin-2 provides sustained augmentation of left ventricular function in mice. Hum Gene Ther. 2013;24(9):777–85. 10.1089/hum.2013.088 23931341 PMC3768340

[12] Lai NC, Gao MH, Giamouridis D, Suarez J, Miyanohara A, Parikh J, et al Intravenous AAV8 Encoding Urocortin-2 Increases Function of the Failing Heart in Mice. Hum Gene Ther. 2015;26(6):347–56. 10.1089/hum.2014.157 25760560 PMC4492611

[13] Kim YC, Giamouridis D, Lai NC, Guo T, Xia B, Fu Z, et al Urocortin 2 Gene Transfer Reduces the Adverse Effects of a Western Diet on Cardiac Function in Mice. Hum Gene Ther. 2019;30(6):693–701. 10.1089/hum.2018.150 30648430 PMC6589493

[14] Giamouridis D, Gao MH, Lai NC, Tan Z, Kim YC, Guo T, et al Effects of Urocortin 2 Versus Urocortin 3 Gene Transfer on Left Ventricular Function and Glucose Disposal. JACC Basic Transl Sci. 2018;3(2):249–64. 10.1016/j.jacbts.2017.12.004 30062211 PMC6059348

[15] Gao MH, Giamouridis D, Lai NC, Walenta E, Paschoal VA, Kim YC, et al One-time injection of AAV8 encoding urocortin 2 provides long-term resolution of insulin resistance. JCI Insight. 2016;1(15):e88322 10.1172/jci.insight.88322 27699250 PMC5033760

[16] Evans AM, DeHaven CD, Barrett T, Mitchell M, Milgram E. Integrated, nontargeted ultrahigh performance liquid chromatography/electrospray ionization tandem mass spectrometry platform for the identification and relative quantification of the small-molecule complement of biological systems. Anal Chem. 2009;81(16):6656–67. 10.1021/ac901536h . 19624122

[17] Dehaven CD, Evans AM, Dai H, Lawton KA. Organization of GC/MS and LC/MS metabolomics data into chemical libraries. J Cheminform. 2010;2(1):9 10.1186/1758-2946-2-9 20955607 PMC2984397

[18] Goodman CA, Mabrey DM, Frey JW, Miu MH, Schmidt EK, Pierre P, et al Novel insights into the regulation of skeletal muscle protein synthesis as revealed by a new nonradioactive in vivo technique. FASEB J. 2011;25(3):1028–39. 10.1096/fj.10-168799 21148113 PMC3042844

[19] Laffel L. Ketone bodies: a review of physiology, pathophysiology and application of monitoring to diabetes. Diabetes Metab Res Rev. 1999;15(6):412–26. 10.1002/(sici)1520-7560(199911/12)15:6<412::aid-dmrr72>3.0.co;2-8 . 10634967

[20] Schmidt EK, Clavarino G, Ceppi M, Pierre P. SUnSET, a nonradioactive method to monitor protein synthesis. Nat Methods. 2009;6(4):275–7. 10.1038/nmeth.1314 . 19305406

[21] Zhang C, Klett EL, Coleman RA. Lipid signals and insulin resistance. Clin Lipidol. 2013;8(6):659–67. 10.2217/clp.13.67 24533033 PMC3921899

[22] Vance JE, Vance DE. Phospholipid biosynthesis in mammalian cells. Biochem Cell Biol. 2004;82(1):113–28. 10.1139/o03-073 . 15052332

[23] Fagone P, Jackowski S. Membrane phospholipid synthesis and endoplasmic reticulum function. J Lipid Res. 2009;50 Suppl:S311–6. 10.1194/jlr.R800049-JLR200 18952570 PMC2674712

[24] Stein LR, Imai S. The dynamic regulation of NAD metabolism in mitochondria. Trends Endocrinol Metab. 2012;23(9):420–8. 10.1016/j.tem.2012.06.005 22819213 PMC3683958

[25] Chen S, Wang Z, Xu B, Mi X, Sun W, Quan N, et al The Modulation of Cardiac Contractile Function by the Pharmacological and Toxicological Effects of Urocortin2. Toxicol Sci. 2015;148(2):581–93. 10.1093/toxsci/kfv202 26342213 PMC5009442

[26] Inoki K, Zhu T, Guan KL. TSC2 mediates cellular energy response to control cell growth and survival. Cell. 2003;115(5):577–90. 10.1016/s0092-8674(03)00929-2 . 14651849

[27] Yang C, Ko B, Hensley CT, Jiang L, Wasti AT, Kim J, et al Glutamine oxidation maintains the TCA cycle and cell survival during impaired mitochondrial pyruvate transport. Mol Cell. 2014;56(3):414–24. 10.1016/j.molcel.2014.09.025 25458842 PMC4268166

[28] Brennan L, Corless M, Hewage C, Malthouse JP, McClenaghan NH, Flatt PR, et al 13C NMR analysis reveals a link between L-glutamine metabolism, D-glucose metabolism and gamma-glutamyl cycle activity in a clonal pancreatic beta-cell line. Diabetologia. 2003;46(11):1512–21. 10.1007/s00125-003-1184-7 . 12955201

[29] Schugar RC, Huang X, Moll AR, Brunt EM, Crawford PA. Role of choline deficiency in the Fatty liver phenotype of mice fed a low protein, very low carbohydrate ketogenic diet. PLoS One. 2013;8(8):e74806 10.1371/journal.pone.0074806 24009777 PMC3756977

[30] Tibbetts AS, Appling DR. Compartmentalization of Mammalian folate-mediated one-carbon metabolism. Annu Rev Nutr. 2010;30:57–81. 10.1146/annurev.nutr.012809.104810 . 20645850

[31] Corbin KD, Zeisel SH. Choline metabolism provides novel insights into nonalcoholic fatty liver disease and its progression. Curr Opin Gastroenterol. 2012;28(2):159–65. 10.1097/MOG.0b013e32834e7b4b 22134222 PMC3601486

[32] Spector AA, Yorek MA. Membrane lipid composition and cellular function. J Lipid Res. 1985;26(9):1015–35. . 3906008

[33] Hapala I, Marza E, Ferreira T. Is fat so bad? Modulation of endoplasmic reticulum stress by lipid droplet formation. Biol Cell. 2011;103(6):271–85. 10.1042/BC20100144 . 21729000

[34] Ying W. NAD+/NADH and NADP+/NADPH in cellular functions and cell death: regulation and biological consequences. Antioxid Redox Signal. 2008;10(2):179–206. 10.1089/ars.2007.1672 . 18020963

[35] Canto C, Menzies KJ, Auwerx J. NAD(+) Metabolism and the Control of Energy Homeostasis: A Balancing Act between Mitochondria and the Nucleus. Cell Metab. 2015;22(1):31–53. 10.1016/j.cmet.2015.05.023 26118927 PMC4487780

[36] Elhassan YS, Philp AA, Lavery GG. Targeting NAD+ in Metabolic Disease: New Insights Into an Old Molecule. J Endocr Soc. 2017;1(7):816–35. 10.1210/js.2017-00092 29264533 PMC5686634

