# Supplementary material for: Significant alteration of liver metabolites by AAV8.Urocortin 2 gene transfer in mice with insulin resistance
Source: PLoS One. 2019 Dec 2;14(12):e0224428. doi: 10.1371/journal.pone.0224428 (PMC6886859; doi:10.1371/journal.pone.0224428)
Supplement: S1 Raw Image — (PDF) [file pone.0224428.s001.pdf]

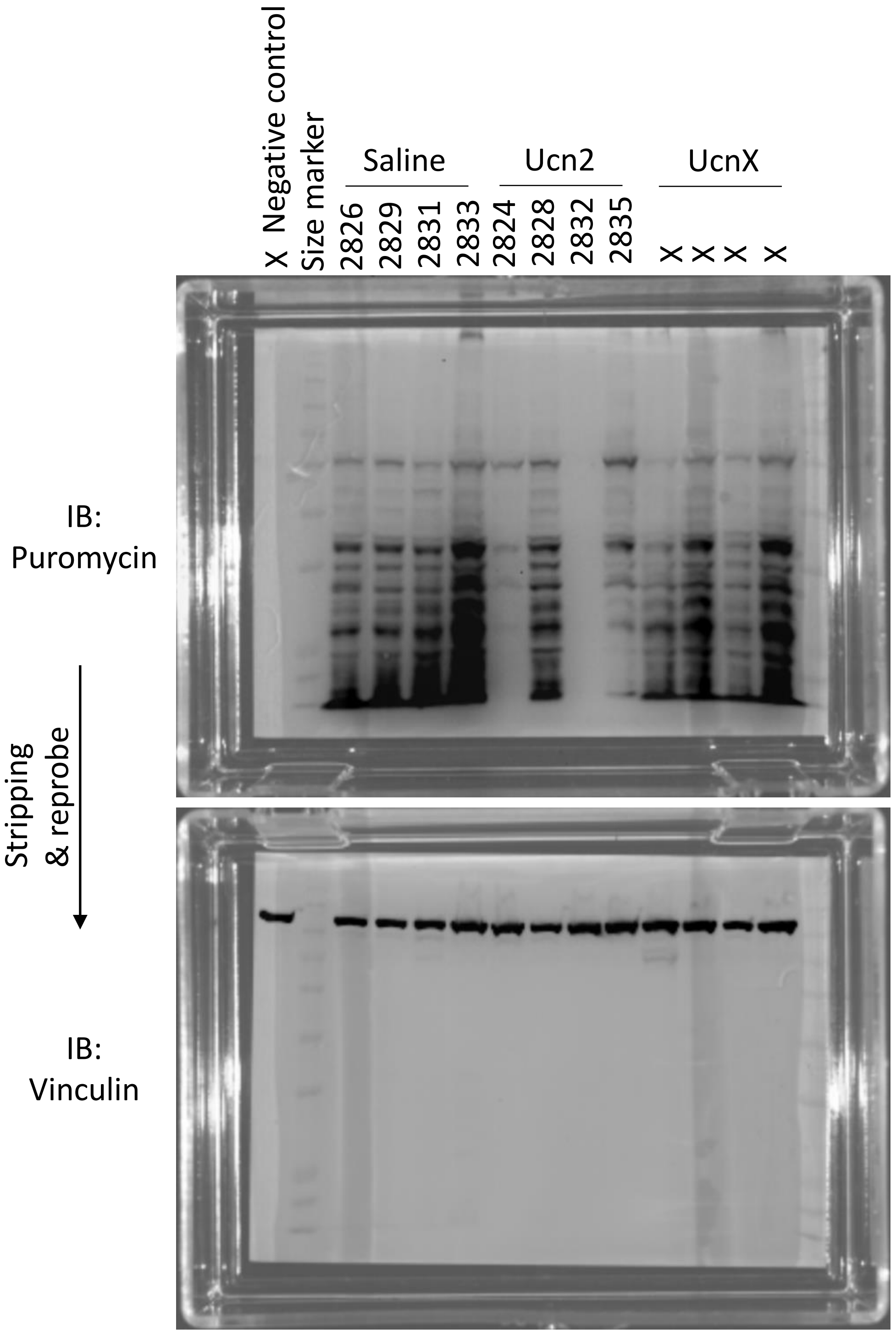

Original images of Figure 3 Immunoblot.
Chemiluminescent images were captured using Chemidoc Gel imaging system (Biorad).
