# Supplementary material for: Significant alteration of liver metabolites by AAV8.Urocortin 2 gene transfer in mice with insulin resistance
Source: PLoS One. 2019 Dec 2;14(12):e0224428. doi: 10.1371/journal.pone.0224428 (PMC6886859; doi:10.1371/journal.pone.0224428)
Supplement: S1 Table — (PDF) [file pone.0224428.s002.pdf]

Supplementary Table 1. Summary of effects on 714 liver metabolites

				CHOW	2 Diets	HF
				AAV8.*Ucn2*	HFD-Saline	AAV8.Empt
Number	Super Pathway	Sub Pathway	Biochemical Name	vs Saline	vs CHOW-Saline	vs Saline
1		Glycine, Serine and Threonine Metabolism	glycine	1.24	0.63	0.92
2			N-acetylglycine	1.78	0.38	0.72
3			sarcosine	1.00	0.99	0.55
4			dimethylglycine	1.12	1.27	0.71
5			betaine	2.19	1.64	0.66
6			betaine aldehyde	2.40	0.25	1.20
7			serine	1.15	0.61	0.84
8			N-acetylserine	1.63	0.70	0.83
9			threonine	1.24	0.63	0.81
10			N-acetylthreonine	2.37	0.59	0.91
11		Alanine and Aspartate Metabolism	alanine	1.02	0.73	0.92
12			N-acetylalanine	1.20	0.71	0.78
13			aspartate	1.47	0.61	0.83
14			N-acetylaspartate (NAA)	1.71	0.43	0.97
15			asparagine	0.99	0.81	0.83
16			N-acetylasparagine	1.12	0.56	0.85
17		Glutamate Metabolism	glutamate	1.28	0.46	0.92
18			glutamine	0.75	1.84	1.12
19			alpha-ketoglutaramate*	0.99	0.81	0.90
20			N-acetylglutamate	1.37	0.73	1.18
21			N-acetylglutamine	0.77	0.70	1.09
22			gamma-carboxyglutamate	1.30	0.69	1.03
23			glutamate, gamma-methyl ester	1.01	0.53	1.00
24			pyroglutamine*	0.96	0.48	0.88
25			carboxyethyl-GABA	0.62	0.49	1.25
26			N-methyl-GABA	1.05	0.57	0.66
27			S-1-pyrroline-5-carboxylate	1.07	0.53	1.96
28		Histidine Metabolism	histidine	2.08	0.75	0.90
29			1-methylhistidine	1.23	0.66	0.90
30			3-methylhistidine	1.49	0.52	0.57
31			N-acetylhistidine	1.10	0.48	1.11
32			N-acetyl-3-methylhistidine*	1.32	0.30	0.69
33			N-acetyl-1-methylhistidine*	1.38	0.24	0.72
34			imidazole propionate	0.27	0.42	2.48
35			formiminoglutamate	5.48	0.25	0.32
36			imidazole lactate	1.21	1.20	1.42
37			anserine	1.79	0.54	0.71
38			1-methylhistamine	0.97	0.25	1.68
39			1-methyl-4-imidazoleacetate	1.11	0.73	1.03
40			1-ribosyl-imidazoleacetate*	1.19	0.11	2.83
41			4-imidazoleacetate	0.94	0.34	1.04
42			lysine	1.19	0.72	0.84
43			N2-acetyllysine	2.06	0.35	0.65
44			N6-acetyllysine	2.03	0.47	0.62
45			N6,N6,N6-trimethyllysine	1.50	0.70	0.78

46	Amino Acid	Lysine Metabolism	5-(galactosylhydroxy)-L-lysine	1.17	**1.59**	**0.80**
47			saccharopine	**1.55**	**1.82**	1.24
48			2-aminoadipate	2.10	1.14	1.06
49			glutarylcarnitine (C5-DC)	**1.36**	**0.82**	1.14
50			pipecolate	1.24	**0.62**	0.90
51			5-aminovalerate	0.64	**0.52**	**1.89**
52			N-trimethyl 5-aminovalerate	4.29	**0.60**	**0.58**
53		Phenylalanine Metabolism	phenylalanine	**1.18**	**0.68**	**0.81**
54			phenylpyruvate	1.16	**0.69**	**0.53**
55			phenyllactate (PLA)	0.96	1.26	1.34
56		Tyrosine Metabolism	tyrosine	1.06	**0.69**	**0.89**
57			4-hydroxyphenylpyruvate	1.44	1.26	1.04
58			3-(4-hydroxyphenyl)lactate	1.14	1.03	**0.78**
59			phenol sulfate	0.87	**0.27**	0.82
60			O-methyltyrosine	1.15	**0.23**	0.94
61			p-cresol glucuronide*	**2.25**	0.89	1.60
62			N-formylphenylalanine	**1.62**	**0.46**	**0.71**
63		Tryptophan Metabolism	tryptophan	**1.16**	**0.73**	**0.85**
64			C-glycosyltryptophan	**1.49**	**0.60**	1.08
65			kynurenine	**3.74**	**0.45**	**0.73**
66			kynurenate	4.74	**0.39**	1.06
67			xanthurenate	0.83	**0.39**	1.02
68			picolinate	1.67	1.15	0.79
69			serotonin	0.80	0.92	0.84
70			5-hydroxyindoleacetate	1.05	**0.31**	0.94
71			indolelactate	**1.46**	**0.26**	1.21
72			indole-3-carboxylate	1.21	**0.56**	0.89
73			indoleacetylglycine	1.19	**0.09**	1.02
74			3-indoxyl sulfate	1.04	**0.35**	1.02
75		Leucine, Isoleucine and Valine Metabolism	leucine	**1.20**	**0.70**	**0.79**
76			N-acetylleucine	1.07	0.86	0.99
77			4-methyl-2-oxopentanoate	0.89	**4.11**	**0.58**
78			isovalerylglycine	**1.66**	**0.26**	**0.74**
79			3-methylcrotonylglycine	**2.78**	**0.18**	0.68
80			beta-hydroxyisovalerate	**1.80**	1.09	**0.75**
81			isoleucine	**1.35**	**0.76**	**0.80**
82			N-acetylisoleucine	**1.34**	0.87	**0.78**
83			alpha-hydroxyisovalerate	1.08	**1.22**	1.01
84			2-methylbutyrylcarnitine (C5)	2.22	0.75	0.87
85			2-methylbutyrylglycine	**2.33**	0.67	**0.69**
86			tiglylcarnitine (C5:1-DC)	**2.29**	0.73	0.81
87			3-hydroxy-2-ethylpropionate	0.80	1.18	0.77
88			ethylmalonate	**0.61**	1.00	0.89
89			methylsuccinate	1.11	**0.71**	0.90
90			valine	**1.32**	**0.68**	**0.75**
91			N-acetylvaline	**1.57**	**0.59**	0.85
92			3-methyl-2-oxobutyrate	0.96	**6.10**	**0.62**
93			2-hydroxy-3-methylvalerate	1.07	0.82	**1.64**
94			isobutyrylcarnitine (C4)	1.11	**0.18**	1.35

95		3-hydroxyisobutyrate	1.13	**0.64**	0.87
96	Methionine, Cysteine, SAM and Taurine Metabolism	methionine	**1.31**	**0.65**	**0.80**
97		N-acetylmethionine	**1.25**	**0.84**	0.95
98		N-formylmethionine	**1.25**	**0.44**	0.87
99		S-methylmethionine	**1.69**	**0.14**	0.29
100		methionine sulfoxide	0.91	**0.81**	**0.67**
101		N-acetylmethionine sulfoxide	1.15	1.31	**0.51**
102		S-adenosylmethionine (SAM)	0.27	**3.59**	1.13
103		S-adenosylhomocysteine (SAH)	1.15	**0.34**	**1.46**
104		cystathionine	0.99	1.17	0.97
105		alpha-ketobutyrate	0.84	**1.89**	0.71
106		cysteine	0.61	0.96	2.14
107		S-methylcysteine	**1.63**	**1.35**	**0.72**
108		cystine	**1.62**	1.26	**0.33**
109		hypotaurine	**2.55**	**0.50**	1.00
110		taurine	**0.55**	1.05	0.95
111		N-acetyltaurine	0.91	0.99	**0.70**
112		taurocyamine	**1.59**	1.10	0.92
113	Urea cycle; Arginine and Proline Metabolism	arginine	1.12	**1.38**	**0.73**
114		urea	**1.60**	**0.67**	**0.73**
115		ornithine	**1.19**	**0.82**	**0.81**
116		2-oxoarginine*	0.95	**0.72**	0.96
117		citrulline	**1.44**	**0.64**	0.87
118		homocitrulline	0.89	**0.12**	**2.62**
119		proline	**1.26**	**0.70**	**0.82**
120		dimethylarginine (SDMA + ADMA)	**1.33**	**0.54**	**0.79**
121		N-acetylarginine	1.10	**0.39**	0.95
122		N-acetylcitrulline	**2.52**	**0.22**	0.95
123		N-delta-acetylornithine	**4.51**	0.72	0.90
124		N-alpha-acetylornithine	**1.98**	1.28	**0.51**
125		trans-4-hydroxyproline	0.85	**0.50**	0.90
126		N-monomethylarginine	**1.21**	**0.56**	**0.84**
127		argininate*	1.20	**1.50**	1.20
128	Creatine Metabolism	guanidinoacetate	1.15	**0.53**	**0.84**
129		creatine	0.89	0.76	0.89
130		creatinine	0.95	0.68	0.85
131	Polyamine Metabolism	putrescine	0.91	**14.55**	**0.51**
132		spermidine	**0.72**	**1.31**	**0.76**
133		5-methylthioadenosine (MTA)	0.92	**2.29**	1.14
134		(N(1) + N(8))-acetylspermidine	**0.68**	**1.64**	**0.74**
135	Guanidino and Acetamido Metabolism	4-guanidinobutanoate	**1.63**	**0.14**	1.18
136		guanidinosuccinate	0.75	0.92	1.30
137	Glutathione Metabolism	glutathione, reduced (GSH)	0.18	0.42	**6.74**
138		glutathione, oxidized (GSSG)	1.09	**0.68**	**1.24**
139		cysteine-glutathione disulfide	1.21	1.06	**0.63**
140		S-methylglutathione	1.22	**1.83**	1.07
141		5-oxoproline	**1.28**	0.95	1.03
142		2-hydroxybutyrate/2-hydroxyisobutyrate	**2.10**	1.08	**0.84**
143		ophthalmate	0.85	0.57	0.82

144			4-hydroxy-nonenal-glutathione	0.51	0.38	2.33
145	Peptide	Gamma-glutamyl Amino Acid	gamma-glutamylglutamate	1.16	0.37	1.06
146			gamma-glutamylglutamine	0.86	0.89	1.07
147			gamma-glutamylglycine	0.88	0.14	0.95
148			gamma-glutamylisoleucine*	1.69	0.57	0.92
149			gamma-glutamylleucine	1.54	0.51	1.23
150			gamma-glutamyl-epsilon-lysine	1.69	2.71	0.76
151			gamma-glutamylphenylalanine	1.59	0.46	1.12
152			gamma-glutamylthreonine	0.71	0.17	1.53
153			gamma-glutamylvaline	1.16	0.49	0.72
154		Dipeptide	glycylisoleucine	1.47	0.55	0.82
155			glycylleucine	1.13	0.55	0.67
156			glycylvaline	1.56	0.57	0.62
157			isoleucylglycine	1.01	0.55	1.10
158			leucylglycine	1.14	0.53	1.19
159			phenylalanylalanine	0.88	0.66	1.01
160			phenylalanylglycine	0.83	1.26	0.75
161			prolylglycine	1.21	0.41	0.63
162			threonylphenylalanine	0.97	0.66	1.33
163			tyrosylglycine	1.17	1.15	0.58
164			valylglutamine	1.08	0.58	1.26
165			valylglycine	1.08	0.73	1.02
166			valylleucine	0.88	0.56	1.77
167		Acetylated Peptides	phenylacetylglycine	1.06	0.32	1.05
168	Carbohydrate	Glycolysis, Gluconeogenesis, and Pyruvate Metabolism	1,5-anhydroglucitol (1,5-AG)	1.52	0.29	1.16
169			glucose	0.38	0.71	1.17
170			glucose 6-phosphate	1.12	0.56	1.38
171			2,3-diphosphoglycerate	0.71	0.71	1.09
172			dihydroxyacetone phosphate (DHAP)	0.70	0.64	0.72
173			3-phosphoglycerate	1.04	1.40	1.27
174			phosphoenolpyruvate (PEP)	1.17	0.95	1.31
175			pyruvate	1.06	1.61	1.07
176			lactate	0.59	0.95	0.91
177			glycerate	0.53	0.72	0.94
178		Pentose Phosphate Pathway	6-phosphogluconate	0.81	0.45	1.45
179			sedoheptulose-7-phosphate	0.81	0.57	1.70
180		Pentose Metabolism	ribose	0.76	0.69	1.00
181			ribitol	1.20	0.52	1.42
182			ribonate	0.72	1.16	1.04
183			arabitol/xylitol	0.84	0.99	0.96
184			ribulose/xylulose	0.47	0.76	0.87
185			arabonate/xylonate	0.88	0.80	1.05
186			sedoheptulose	0.32	0.37	1.83
187			ribulonate/xylulonate*	0.54	0.56	1.22
188		Glycogen Metabolism	maltopentaose	0.09	0.77	4.35
189			maltotetraose	0.01	0.65	2.92
190			maltotriose	0.06	0.67	2.04
191			maltose	0.10	0.64	1.10
192			fructose	0.12	0.33	1.67

193		Fructose, Mannose and Galactose Metabolism	mannitol/sorbitol	0.32	0.56	1.94
194			mannose	0.37	0.51	1.42
195			galactose 1-phosphate	0.94	0.58	1.03
196			2-ketogulonate	0.94	0.50	1.03
197			galactonate	1.00	0.31	1.34
198		Nucleotide Sugar	UDP-glucose/UDP-galactose	3.21	1.29	1.42
199			UDP-N-acetylglucosamine/galactosamine	0.58	0.62	1.47
200		Aminosugar Metabolism	glucuronate	0.67	0.68	0.97
201			N-acetylglucosamine 6-phosphate	0.62	0.95	1.35
202			N-acetyl-glucosamine 1-phosphate	0.51	0.40	1.29
203			N-acetylneuraminate	1.45	0.73	0.87
204			N-acetylglucosaminylasparagine	1.26	1.14	1.18
205			erythronate*	0.89	1.05	0.91
206			N-acetylglucosamine/N-acetylgalactosamine	1.85	0.70	0.55
207		Advanced Glycation End-product	N6-carboxymethyllysine	1.39	0.14	1.00
208	Energy	TCA Cycle	citrate	1.15	0.54	1.41
209			aconitate [cis or trans]	1.25	0.75	0.98
210			alpha-ketoglutarate	1.24	1.02	1.05
211			succinylcarnitine (C4-DC)	0.68	1.56	1.13
212			succinate	1.04	1.58	1.02
213			fumarate	1.31	0.94	1.00
214			malate	1.20	1.06	1.04
215			itaconate	1.70	1.73	0.55
216			tricarballylate	0.88	0.86	0.86
217			2-methylcitrate/homocitrate	1.24	0.66	0.87
218		Oxidative Phosphorylation	acetylphosphate	1.21	0.76	1.17
219			phosphate	1.05	0.80	1.02
220		Fatty Acid Synthesis	malonylcarnitine	1.43	0.46	1.01
221			malonate	1.08	1.05	1.04
222		Medium Chain Fatty Acid	5-dodecenoate (12:1n7)	1.64	0.57	0.87
223		Long Chain Fatty Acid	myristate (14:0)	0.44	2.62	0.40
224			myristoleate (14:1n5)	1.06	1.71	0.59
225			pentadecanoate (15:0)	0.63	1.05	0.64
226			palmitate (16:0)	0.62	1.31	0.64
227			palmitoleate (16:1n7)	0.42	2.01	0.45
228			margarate (17:0)	0.54	1.43	0.39
229			10-heptadecenoate (17:1n7)	0.45	2.30	0.37
230			stearate (18:0)	0.60	1.15	0.55
231			oleate/vaccenate (18:1)	0.55	1.69	0.58
232			nonadecanoate (19:0)	0.58	0.61	0.44
233			10-nonadecenoate (19:1n9)	0.52	1.84	0.36
234			arachidate (20:0)	0.36	0.85	0.49
235			eicosenoate (20:1)	0.40	2.17	0.39
236			behenate (22:0)*	0.35	0.60	0.32
237			erucate (22:1n9)	0.36	0.77	0.41
238			heneicosapentaenoate (21:5n3)	0.40	0.83	0.44
239			hexadecadienoate (16:2n6)	0.78	0.86	0.52
240			stearidonate (18:4n3)	0.62	0.80	0.52

241	Polyunsaturated Fatty Acid (n3 and n6)	eicosapentaenoate (EPA; 20:5n3)	0.47	0.62	0.38
242		docosapentaenoate (n3 DPA; 22:5n3)	0.55	1.10	0.35
243		docosahexaenoate (DHA; 22:6n3)	0.67	1.51	0.51
244		docosatrienoate (22:3n3)	0.52	1.21	0.48
245		nisinate (24:6n3)	0.62	1.32	0.44
246		linoleate (18:2n6)	0.65	0.95	0.56
247		linolenate [alpha or gamma; (18:3n3 or 6)]	0.56	0.79	0.40
248		dihomo-linolenate (20:3n3 or n6)	0.59	1.27	0.50
249		arachidonate (20:4n6)	0.65	1.12	0.64
250		adrenate (22:4n6)	0.64	1.79	0.42
251		docosapentaenoate (n6 DPA; 22:5n6)	0.85	1.89	0.53
252		docosadienoate (22:2n6)	0.48	1.40	0.36
253		dihomo-linoleate (20:2n6)	0.52	1.98	0.34
254		mead acid (20:3n9)	0.70	2.99	0.51
255		docosatrienoate (22:3n6)*	0.50	4.64	0.42
256	Fatty Acid, Branched	15-methylpalmitate (i17:0)	0.64	1.23	0.45
257		17-methylstearate (i19:0)	0.55	0.85	0.36
258	Fatty Acid, Dicarboxylate	glutarate (C5-DC)	1.17	1.22	1.08
259		3-methylglutarate/2-methylglutarate	1.21	0.61	0.90
260		2-hydroxyglutarate	0.83	1.35	1.06
261		adipate (C6-DC)	1.52	0.78	0.81
262		2-hydroxyadipate	1.44	0.57	0.77
263		3-hydroxyadipate*	1.40	0.46	0.71
264		3-methyladipate	1.86	0.42	1.00
265		pimelate (C7-DC)	1.19	0.69	1.16
266		suberate (C8-DC)	0.76	0.89	1.17
267		azelate (C9-DC)	1.04	0.66	0.97
268		sebacate (C10-DC)	1.39	0.81	0.97
269		dodecadienoate (12:2)*	0.69	0.71	1.24
270		dodecanedioate (C12-DC)	1.27	0.69	0.93
271		tetradecanedioate (C14-DC)	1.61	1.20	0.97
272		hexadecanedioate (C16-DC)	2.01	0.86	1.02
273		hexadecenedioate (C16:1-DC)*	1.43	1.88	0.90
274		octadecanedioate (C18-DC)	0.84	0.38	0.90
275	Fatty Acid, Amino	2-aminooctanoate	1.06	0.50	0.81
276	Fatty Acid Metabolism (also BCAA Metabolism)	butyrylglycine	1.38	0.47	0.93
277		propionylcarnitine (C3)	1.69	0.54	1.05
278		propionylglycine	4.60	0.23	0.64
279		methylmalonate (MMA)	0.83	1.40	0.75
280	Fatty Acid Metabolism(Acyl Glycine)	isocaproylglycine	1.15	0.20	0.93
281		valerylglycine	2.61	0.65	0.83
282		hexanoylglycine	1.16	0.17	1.22
283		3,4-methylene heptanoylglycine	1.09	0.37	1.10
284		N-octanoylglycine	1.20	0.04	1.47
285		N-palmitoylglycine	0.71	0.33	0.46
286		N-linoleoylglycine	1.10	0.25	1.00
287		acetylcarnitine (C2)	1.82	1.22	0.72
288		3-hydroxybutyrylcarnitine (1)	0.94	1.32	0.94
289		3-hydroxybutyrylcarnitine (2)	0.90	1.82	1.22

290	Fatty Acid Metabolism(Acyl Carnitine)	palmitoylcarnitine (C16)	**2.06**	**2.33**	1.02
291		stearoylcarnitine (C18)	**1.78**	1.37	1.06
292		linoleoylcarnitine (C18:2)*	**1.79**	**0.61**	1.10
293		oleoylcarnitine (C18:1)	**1.57**	**2.76**	0.95
294		adipoylcarnitine (C6-DC)	1.09	**0.59**	0.92
295		pimeloylcarnitine/3-methyladipoylcarnitine (C7-DC)	1.23	**0.27**	1.35
296		arachidoylcarnitine (C20)*	1.25	**1.39**	1.05
297		arachidonoylcarnitine (C20:4)	1.68	0.88	1.15
298		behenoylcarnitine (C22)*	0.61	0.63	1.00
299		eicosenoylcarnitine (C20:1)*	1.64	**2.82**	1.15
300		erucoylcarnitine (C22:1)*	0.61	**1.84**	0.73
301		lignoceroylcarnitine (C24)*	1.23	0.83	0.82
302	Carnitine Metabolism	deoxycarnitine	0.80	**0.32**	1.05
303		carnitine	1.08	1.14	1.00
304	Ketone Bodies	3-hydroxybutyrate (BHBA)	**2.12**	**1.29**	**0.76**
305	Fatty Acid Metabolism (Acyl Choline)	palmitoylcholine	**4.52**	0.98	1.01
306		oleoylcholine	**2.73**	1.36	1.19
307		palmitoloelycholine	**2.85**	**0.57**	0.91
308		linoleoylcholine*	**4.40**	**0.30**	1.24
309		stearoylcholine*	**3.92**	0.98	1.04
310		arachidonoylcholine	**8.60**	0.93	0.98
311	Fatty Acid, Monohydroxy	2-hydroxyheptanoate*	0.52	0.83	1.53
312		2-hydroxypalmitate	**0.63**	**0.83**	0.98
313		2-hydroxystearate	0.84	0.80	1.68
314		3-hydroxyhexanoate	0.78	0.84	1.10
315		3-hydroxyoctanoate	0.75	0.89	1.02
316		3-hydroxydecanoate	1.63	1.03	0.91
317		3-hydroxyoleate*	**0.58**	**1.35**	**0.57**
318		8-hydroxyoctanoate	1.10	0.96	0.94
319		16-hydroxypalmitate	1.66	0.75	1.10
320		4-HDoHE	**0.36**	**0.33**	0.76
321		13-HODE + 9-HODE	0.73	**0.32**	0.91
322		14-HDoHE/17-HDoHE	0.85	0.75	1.37
323	Fatty Acid, Dihydroxy	12,13-DiHOME	0.95	**0.61**	0.83
324		9,10-DiHOME	0.75	**0.42**	0.89
325		19,20-DiHDPA	1.26	**1.95**	1.04
326		5,6-DiHETrE	0.62	**0.52**	1.08
327		14,15-DiHETrE	1.14	**0.62**	1.12
328	Fatty Acid, Oxidized	4-hydroxy-2-nonenal	0.76	**0.28**	1.40
329	Eicosanoid	prostaglandin F2alpha	1.24	**0.66**	1.42
330		5-HETE	**0.31**	**0.25**	1.29
331		12-HETE	**0.69**	**0.79**	**1.80**
332		15-HETE	0.65	**0.35**	1.18
333		12-HHTrE	1.40	0.79	**0.69**
334		oleoyl ethanolamide	1.14	0.97	**0.75**
335		palmitoyl ethanolamide	1.17	1.00	**0.82**
336		stearoyl ethanolamide	**1.26**	0.90	0.92
337		N-arachidonoyltaurine	**0.40**	**0.63**	**0.46**
338		N-oleoyltaurine	**0.24**	1.48	**0.28**

339	Endocannabinoid	N-stearoyltaurine	0.48	1.56	0.32
340		N-palmitoyltaurine	0.16	1.12	0.19
341		N-palmitoleoyltaurine*	0.19	0.79	0.42
342		N-linoleoyltaurine*	0.32	0.35	0.41
343		N-linolenoyltaurine*	0.34	0.15	0.54
344		linoleoyl ethanolamide	0.61	0.35	0.39
345	Inositol Metabolism	myo-inositol	1.32	0.62	0.93
346		inositol 1-phosphate (I1P)	2.15	1.37	0.69
347	Phospholipid Metabolism	choline	1.14	0.94	0.98
348		choline phosphate	1.18	1.39	1.30
349		cytidine 5'-diphosphocholine	0.74	0.67	1.22
350		glycerophosphorylcholine (GPC)	0.94	2.73	0.84
351		phosphoethanolamine	1.28	1.29	0.75
352		cytidine-5'-diphosphoethanolamine	0.53	0.44	0.93
353		glycerophosphoethanolamine	0.94	1.61	0.77
354		glycerophosphoserine*	1.19	1.14	0.84
355		glycerophosphoinositol*	1.41	0.86	0.90
356		trimethylamine N-oxide	0.78	1.03	1.23
357	Phosphatidylcholine (PC)	1-myristoyl-2-palmitoyl-GPC (14:0/16:0)	1.05	0.81	0.99
358		1-myristoyl-2-arachidonoyl-GPC (14:0/20:4)*	0.98	0.70	1.02
359		1,2-dipalmitoyl-GPC (16:0/16:0)	1.26	0.89	0.91
360		1-palmitoyl-2-palmitoleoyl-GPC (16:0/16:1)*	0.88	0.78	0.94
361		1-palmitoyl-2-stearoyl-GPC (16:0/18:0)	1.25	1.12	1.01
362		1-palmitoyl-2-oleoyl-GPC (16:0/18:1)	1.08	1.08	0.95
363		1-palmitoyl-2-linoleoyl-GPC (16:0/18:2)	1.12	0.62	1.05
364		1-palmitoyl-2-gamma-linolenoyl-GPC (16:0/18:3n6)*	0.93	0.58	1.10
365		1-palmitoyl-2-dihomo-linolenoyl-GPC (16:0/20:3n3 or 6)*	0.95	1.15	1.00
366		1-palmitoyl-2-arachidonoyl-GPC (16:0/20:4n6)	1.02	0.94	1.05
367		1-palmitoyl-2-docosahexaenoyl-GPC (16:0/22:6)	1.09	0.82	1.03
368		1-palmitoleoyl-2-linoleoyl-GPC (16:1/18:2)*	0.97	0.32	0.95
369		1-stearoyl-2-oleoyl-GPC (18:0/18:1)	0.98	1.42	0.98
370		1-stearoyl-2-linoleoyl-GPC (18:0/18:2)*	1.02	0.83	1.04
371		1-stearoyl-2-arachidonoyl-GPC (18:0/20:4)	0.95	1.12	1.04
372		1-stearoyl-2-docosahexaenoyl-GPC (18:0/22:6)	0.99	0.91	1.07
373		1-oleoyl-2-linoleoyl-GPC (18:1/18:2)*	0.99	0.69	0.90
374		1-oleoyl-2-docosahexaenoyl-GPC (18:1/22:6)*	1.09	0.74	0.91
375		1,2-dilinoleoyl-GPC (18:2/18:2)	1.29	0.28	1.13
376		1-linoleoyl-2-linolenoyl-GPC (18:2/18:3)*	1.56	0.06	0.92
377		1-linoleoyl-2-arachidonoyl-GPC (18:2/20:4n6)*	1.12	0.51	1.13
378		1,2-dipalmitoyl-GPE (16:0/16:0)*	1.31	0.88	0.80
379		1-palmitoyl-2-stearoyl-GPE (16:0/18:0)*	1.43	1.01	0.77
380		1-palmitoyl-2-oleoyl-GPE (16:0/18:1)	1.19	0.75	0.82
381		1-palmitoyl-2-linoleoyl-GPE (16:0/18:2)	1.15	0.21	0.84
382		1-palmitoyl-2-arachidonoyl-GPE (16:0/20:4)*	1.06	0.63	0.94
383		1-palmitoyl-2-docosahexaenoyl-GPE (16:0/22:6)*	1.08	0.70	0.91
384		1-stearoyl-2-oleoyl-GPE (18:0/18:1)	1.19	1.13	0.90

385	Lipid	Phosphatidylethanolamine (PE)	1-stearoyl-2-linoleoyl-GPE (18:0/18:2)*	1.13	0.51	1.10
386			1-stearoyl-2-arachidonoyl-GPE (18:0/20:4)	1.05	0.76	1.05
387			1-stearoyl-2-docosahexaenoyl-GPE (18:0/22:6)*	1.13	0.69	0.93
388			1-oleoyl-2-linoleoyl-GPE (18:1/18:2)*	1.18	0.33	0.94
389			1-oleoyl-2-arachidonoyl-GPE (18:1/20:4)*	1.05	0.74	0.90
390			1-oleoyl-2-docosahexaenoyl-GPE (18:1/22:6)*	1.05	0.73	0.85
391			1,2-dilinoleoyl-GPE (18:2/18:2)*	1.17	0.09	1.11
392			1-linoleoyl-2-arachidonoyl-GPE (18:2/20:4)*	1.11	0.28	0.99
393		Phosphatidylserine (PS)	1-stearoyl-2-oleoyl-GPS (18:0/18:1)	1.28	0.84	1.05
394			1-stearoyl-2-arachidonoyl-GPS (18:0/20:4)	1.12	0.68	1.09
395		Phosphatidylglycerol (PG)	1-palmitoyl-2-oleoyl-GPG (16:0/18:1)	1.11	0.77	1.09
396			1-palmitoyl-2-linoleoyl-GPG (16:0/18:2)	1.17	0.57	1.25
397		Phosphatidylinositol (PI)	1-palmitoyl-2-linoleoyl-GPI (16:0/18:2)	1.51	0.15	1.10
398			1-palmitoyl-2-arachidonoyl-GPI (16:0/20:4)*	1.09	0.58	1.03
399			1-stearoyl-2-linoleoyl-GPI (18:0/18:2)	1.61	0.24	1.14
400			1-oleoyl-2-linoleoyl-GPI (18:1/18:2)*	1.38	0.12	0.79
401			1-stearoyl-2-arachidonoyl-GPI (18:0/20:4)	1.07	0.71	1.07
402			1-oleoyl-2-arachidonoyl-GPI (18:1/20:4) *	1.29	0.69	0.85
403		Lysophospholipid	1-palmitoyl-GPC (16:0)	1.05	0.88	1.04
404			2-palmitoyl-GPC (16:0)*	0.58	0.90	0.50
405			1-palmitoleoyl-GPC (16:1)*	0.81	0.57	0.95
406			1-stearoyl-GPC (18:0)	0.93	1.23	1.11
407			1-oleoyl-GPC (18:1)	1.02	0.99	0.85
408			1-linoleoyl-GPC (18:2)	0.60	0.60	0.45
409			1-linolenoyl-GPC (18:3)*	1.07	0.28	0.93
410			1-arachidonoyl-GPC (20:4n6)*	0.52	1.31	0.39
411			1-palmitoyl-GPE (16:0)	1.02	0.55	0.91
412			1-stearoyl-GPE (18:0)	0.95	0.77	1.06
413			2-stearoyl-GPE (18:0)*	0.49	0.88	0.50
414			1-oleoyl-GPE (18:1)	1.01	0.70	0.89
415			1-linoleoyl-GPE (18:2)*	1.27	0.30	0.89
416			1-arachidonoyl-GPE (20:4n6)*	0.52	0.73	0.46
417			1-palmitoyl-GPS (16:0)*	0.65	0.54	0.58
418			1-stearoyl-GPS (18:0)*	1.26	0.66	1.22
419			1-oleoyl-GPS (18:1)	0.56	0.77	0.36
420			1-palmitoyl-GPG (16:0)*	0.46	1.05	0.46
421			1-stearoyl-GPG (18:0)	0.43	1.44	0.59
422			1-oleoyl-GPG (18:1)*	0.55	4.14	0.17
423			1-linoleoyl-GPG (18:2)*	0.50	1.07	0.43
424			1-palmitoyl-GPI (16:0)	0.56	0.63	0.45
425			1-stearoyl-GPI (18:0)	0.58	0.86	0.52
426			1-oleoyl-GPI (18:1)*	0.66	0.72	0.29
427			1-linoleoyl-GPI (18:2)*	0.85	0.18	0.45
428			1-arachidonoyl-GPI (20:4)*	0.57	1.01	0.48
429			1-(1-enyl-palmitoyl)-2-oleoyl-GPE (P-16:0/18:1)*	1.17	0.82	0.78
430			1-(1-enyl-palmitoyl)-2-linoleoyl-GPE (P-16:0/18:2)*	1.18	0.32	0.64
431			1-(1-enyl-palmitoyl)-2-palmitoyl-GPC (P-16:0/16:0)*	1.26	0.47	1.00

432		Plasmalogen	1-(1-enyl-palmitoyl)-2-arachidonoyl-GPE (P-16:0/20:4)*	1.28	0.77	0.86
433			1-(1-enyl-palmitoyl)-2-oleoyl-GPC (P-16:0/18:1)*	0.98	0.55	0.83
434			1-(1-enyl-stearoyl)-2-oleoyl-GPE (P-18:0/18:1)	1.38	0.67	0.75
435			1-(1-enyl-stearoyl)-2-linoleoyl-GPE (P-18:0/18:2)*	1.15	0.47	0.77
436			1-(1-enyl-stearoyl)-2-arachidonoyl-GPE (P-18:0/20:4)*	1.27	1.08	0.96
437		Lysoplasmalogen	1-(1-enyl-palmitoyl)-GPE (P-16:0)*	1.23	0.88	0.78
438			1-(1-enyl-oleoyl)-GPE (P-18:1)*	1.08	0.94	0.85
439			1-(1-enyl-stearoyl)-GPE (P-18:0)*	1.19	1.11	0.80
440		Glycerolipid Metabolism	glycerol	0.93	1.97	0.78
441			glycerol 3-phosphate	0.98	0.16	1.92
442			glycerophosphoglycerol	0.80	2.65	1.00
443		Monoacylglycerol	1-myristoylglycerol (14:0)	0.29	1.55	0.44
444			1-palmitoylglycerol (16:0)	1.19	2.27	0.65
445			1-palmitoleoylglycerol (16:1)*	0.28	1.46	0.43
446			1-margaroylglycerol (17:0)	0.57	1.12	0.33
447			1-stearoylglycerol (18:0)	0.96	3.06	0.68
448			1-oleoylglycerol (18:1)	1.02	1.56	0.80
449			1-linoleoylglycerol (18:2)	1.71	0.74	0.83
450			1-linolenoylglycerol (18:3)	1.32	0.94	0.96
451			1-dihomo-linolenylglycerol (20:3)	1.63	0.66	0.96
452			1-arachidonylglycerol (20:4)	1.50	0.42	1.17
453			1-docosahexaenoylglycerol (22:6)	1.30	1.24	1.17
454			2-myristoylglycerol (14:0)	0.36	1.11	0.62
455			2-palmitoylglycerol (16:0)	0.63	0.95	0.42
456			2-palmitoleoylglycerol (16:1)*	0.23	0.76	0.56
457			2-stearoylglycerol (18:0)	1.33	0.73	1.07
458			2-oleoylglycerol (18:1)	0.90	0.91	1.13
459			2-linoleoylglycerol (18:2)	0.82	0.30	1.29
460			2-arachidonoylglycerol (20:4)	0.54	0.28	0.73
461			2-docosahexaenoylglycerol (22:6)*	0.54	1.09	0.77
462			1-dihomo-linoleoylglycerol (20:2)	1.74	0.96	0.76
463			diacylglycerol (12:0/18:1, 14:0/16:1, 16:0/14:1) [1]*	0.72	8.05	0.83
464			diacylglycerol (12:0/18:1, 14:0/16:1, 16:0/14:1) [2]*	0.64	2.28	0.88
465			diacylglycerol (14:0/18:1, 16:0/16:1) [1]*	0.65	3.43	0.90
466			diacylglycerol (14:0/18:1, 16:0/16:1) [2]*	0.67	2.11	0.96
467			diacylglycerol (16:1/18:2 [2], 16:0/18:3 [1])*	0.74	0.75	0.85
468			palmitoyl-myristoyl-glycerol (16:0/14:0) [1]*	0.88	3.12	0.96
469			palmitoyl-myristoyl-glycerol (16:0/14:0) [2]	0.92	1.94	1.04
470			palmitoyl-palmitoyl-glycerol (16:0/16:0) [1]*	0.98	3.00	0.99
471			palmitoyl-palmitoyl-glycerol (16:0/16:0) [2]*	1.18	1.09	1.01
472			palmitoleoyl-palmitoleoyl-glycerol (16:1/16:1) [2]*	0.62	1.85	0.83
473			palmitoyl-oleoyl-glycerol (16:0/18:1) [1]*	0.85	2.85	0.89
474			palmitoyl-oleoyl-glycerol (16:0/18:1) [2]*	0.90	1.59	1.00
475			palmitoyl-linoleoyl-glycerol (16:0/18:2) [1]*	1.07	1.73	0.88
476			palmitoyl-linoleoyl-glycerol (16:0/18:2) [2]*	1.01	1.11	1.01
477			palmitoyl-linolenoyl-glycerol (16:0/18:3) [2]*	1.08	0.39	0.47

478	Diacylglycerol	palmitoleoyl-oleoyl-glycerol (16:1/18:1) [2]*	0.57	1.75	0.86
479		palmitoleoyl-linoleoyl-glycerol (16:1/18:2) [1]*	0.75	1.28	0.30
480		palmitoyl-arachidonoyl-glycerol (16:0/20:4) [1]*	1.70	1.80	0.94
481		palmitoyl-arachidonoyl-glycerol (16:0/20:4) [2]*	1.20	0.91	1.10
482		palmitoleoyl-arachidonoyl-glycerol (16:1/20:4) [2]*	0.78	0.60	0.82
483		palmitoyl-docosahexaenoyl-glycerol (16:0/22:6) [2]*	1.40	2.70	1.23
484		stearoyl-linoleoyl-glycerol (18:0/18:2) [2]*	1.32	0.76	0.94
485		oleoyl-oleoyl-glycerol (18:1/18:1) [1]*	0.83	2.24	0.73
486		oleoyl-oleoyl-glycerol (18:1/18:1) [2]*	0.83	1.42	0.83
487		oleoyl-linoleoyl-glycerol (18:1/18:2) [1]	1.08	0.97	0.78
488		oleoyl-linoleoyl-glycerol (18:1/18:2) [2]	0.91	0.70	0.88
489		oleoyl-linolenoyl-glycerol (18:1/18:3) [2]*	0.74	0.08	0.93
490		linoleoyl-linoleoyl-glycerol (18:2/18:2) [1]*	1.42	0.35	0.87
491		linoleoyl-linoleoyl-glycerol (18:2/18:2) [2]*	1.33	0.20	0.84
492		linoleoyl-linolenoyl-glycerol (18:2/18:3) [1]*	1.27	0.26	1.12
493		linoleoyl-linolenoyl-glycerol (18:2/18:3) [2]*	0.91	0.31	0.83
494		stearoyl-arachidonoyl-glycerol (18:0/20:4) [1]*	1.83	1.25	0.80
495		stearoyl-arachidonoyl-glycerol (18:0/20:4) [2]*	1.17	0.50	0.95
496		oleoyl-arachidonoyl-glycerol (18:1/20:4) [1]*	3.52	7.25	0.77
497		oleoyl-arachidonoyl-glycerol (18:1/20:4) [2]*	0.91	1.04	0.88
498		linoleoyl-arachidonoyl-glycerol (18:2/20:4) [1]*	1.54	0.65	1.14
499		linoleoyl-arachidonoyl-glycerol (18:2/20:4) [2]*	1.24	0.47	1.10
500		stearoyl-docosahexaenoyl-glycerol (18:0/22:6) [2]*	1.34	0.67	1.10
501		linoleoyl-docosahexaenoyl-glycerol (18:2/22:6) [2]*	1.02	0.33	0.90
502	Sphingolipid Synthesis	sphinganine	2.09	2.33	0.84
503		sphingadienine	1.11	1.13	0.76
504		phytosphingosine	1.19	1.45	0.87
505	Dihydroceramides	N-palmitoyl-sphinganine (d18:0/16:0)	1.42	1.53	0.93
506		N-stearoyl-sphinganine (d18:0/18:0)*	1.70	5.22	0.80
507	Ceramides	N-palmitoyl-sphingosine (d18:1/16:0)	1.42	1.53	0.85
508		N-(2-hydroxypalmitoyl)-sphingosine (d18:1/16:0(2OH))	1.14	2.55	0.83
509		N-stearoyl-sphingosine (d18:1/18:0)*	1.11	2.37	0.97
510		N-palmitoyl-sphingadienine (d18:2/16:0)*	1.40	1.26	0.92
511		N-behenoyl-sphingadienine (d18:2/22:0)*	0.90	0.44	1.09
512		N-palmitoyl-heptadecasphingosine (d17:1/16:0)*	1.44	1.64	0.95
513		ceramide (d18:1/14:0, d16:1/16:0)*	1.25	3.04	0.94
514		ceramide (d18:1/17:0, d17:1/18:0)*	1.35	1.74	1.00
515		ceramide (d18:1/20:0, d16:1/22:0, d20:1/18:0)*	0.87	1.29	1.07
516		ceramide (d16:1/24:1, d18:1/22:1)*	0.82	0.76	1.01
517		ceramide (d18:2/24:1, d18:1/24:2)*	1.29	0.75	0.91
518		glycosyl-N-palmitoyl-sphingosine (d18:1/16:0)	1.50	0.97	0.92
519		glycosyl-N-stearoyl-sphingosine (d18:1/18:0)	1.20	2.32	1.03

520		Hexosylceramides (HCER)	glycosyl-N-behenoyl-sphingadienine (d18:2/22:0)*	1.03	0.36	1.37
521			glycosyl ceramide (d18:1/20:0, d16:1/22:0)*	1.05	1.15	1.15
522			glycosyl ceramide (d16:1/24:1, d18:1/22:1)*	1.14	0.60	1.07
523			glycosyl ceramide (d18:1/23:1, d17:1/24:1)*	1.93	0.35	1.18
524			glycosyl ceramide (d18:2/24:1, d18:1/24:2)*	1.79	0.63	0.85
525		Lactosylceramides (LCER)	lactosyl-N-palmitoyl-sphingosine (d18:1/16:0)	1.60	1.89	0.83
526			lactosyl-N-nervonoyl-sphingosine (d18:1/24:1)*	1.72	0.97	0.90
527		Dihydrosphingomyelins	palmitoyl dihydrosphingomyelin (d18:0/16:0)*	1.40	0.89	0.98
528			behenoyl dihydrosphingomyelin (d18:0/22:0)*	1.00	0.86	1.02
529			sphingomyelin (d18:0/18:0, d19:0/17:0)*	1.48	1.06	1.04
530			sphingomyelin (d18:0/20:0, d16:0/22:0)*	0.99	1.04	1.02
531		Sphingomyelins	palmitoyl sphingomyelin (d18:1/16:0)	1.14	1.18	0.94
532			stearoyl sphingomyelin (d18:1/18:0)	1.01	1.34	0.96
533			behenoyl sphingomyelin (d18:1/22:0)*	0.94	0.29	1.13
534			tricosanoyl sphingomyelin (d18:1/23:0)*	1.25	0.61	0.85
535			lignoceroyl sphingomyelin (d18:1/24:0)	1.09	0.39	0.99
536			sphingomyelin (d18:2/23:1)*	1.39	0.31	0.84
537			sphingomyelin (d18:2/24:2)*	1.81	0.27	1.00
538			sphingomyelin (d18:1/14:0, d16:1/16:0)*	0.97	1.43	0.87
539			sphingomyelin (d17:1/16:0, d18:1/15:0, d16:1/17:0)*	1.06	0.88	0.89
540			sphingomyelin (d18:2/16:0, d18:1/16:1)*	1.14	0.47	0.93
541			sphingomyelin (d18:1/17:0, d17:1/18:0, d19:1/16:0)	1.19	1.11	1.01
542			sphingomyelin (d18:1/18:1, d18:2/18:0)	1.00	1.14	0.96
543			sphingomyelin (d18:1/19:0, d19:1/18:0)*	0.97	0.56	1.08
544			sphingomyelin (d18:1/20:0, d16:1/22:0)*	0.95	0.65	1.11
545			sphingomyelin (d18:1/20:1, d18:2/20:0)*	0.76	0.48	0.94
546			sphingomyelin (d18:1/21:0, d17:1/22:0, d16:1/23:0)*	0.95	0.29	1.14
547			sphingomyelin (d18:2/21:0, d16:2/23:0)*	0.84	0.23	1.03
548			sphingomyelin (d18:1/22:1, d18:2/22:0, d16:1/24:1)*	0.88	0.32	1.03
549			sphingomyelin (d18:1/22:2, d18:2/22:1, d16:1/24:2)*	1.03	0.37	0.93
550			sphingomyelin (d18:2/23:0, d18:1/23:1, d17:1/24:1)*	1.23	0.39	0.93
551			sphingomyelin (d18:1/24:1, d18:2/24:0)*	1.29	0.49	0.88
552			sphingomyelin (d18:2/24:1, d18:1/24:2)*	1.35	0.45	0.90
553		Sphingosines	sphingosine	1.22	1.64	0.78
554			hexadecasphingosine (d16:1)*	1.31	2.78	0.81
555			heptadecasphingosine (d17:1)	1.14	1.35	0.73
556		Mevalonate Metabolism	3-hydroxy-3-methylglutarate	1.01	0.57	1.08
557		Sterol	cholesterol	1.27	0.91	0.91
558			7-alpha-hydroxy-3-oxo-4-cholestenoate (7-Hoca)	0.92	0.88	0.86
559			4-cholesten-3-one	0.78	5.18	1.09
560			beta-sitosterol	1.50	0.50	1.00
561			campesterol	1.23	0.04	1.16
562			7-hydroxycholesterol (alpha or beta)	0.47	0.65	2.24
563		Corticosteroids	corticosterone	2.14	1.22	0.89

564		Primary Bile Acid Metabolism	cholate	1.62	**0.40**	1.05
565			glycocholate	1.69	1.22	0.49
566			taurocholate	1.16	0.69	0.69
567			chenodeoxycholate	0.91	0.44	1.25
568			taurochenodeoxycholate	0.72	0.61	0.68
569			beta-muricholate	0.95	0.65	0.74
570			alpha-muricholate	1.34	**0.34**	1.23
571			tauro-beta-muricholate	0.85	0.56	0.62
572		Secondary Bile Acid Metabolism	deoxycholate	**1.82**	**0.21**	0.73
573			taurodeoxycholate	**1.67**	**0.22**	0.80
574			6-beta-hydroxylithocholate	1.15	**0.18**	0.93
575			taurolithocholate	0.90	**0.18**	**0.71**
576			ursodeoxycholate	0.98	**0.35**	1.19
577			tauroursodeoxycholate	0.76	0.40	0.87
578			6-oxolithocholate	1.24	**0.06**	0.89
579			hyodeoxycholate	1.04	**0.08**	1.18
580			taurohyodeoxycholic acid	0.61	0.14	0.95
581			3-dehydrocholate	2.17	**0.51**	0.56
582			7-ketodeoxycholate	1.14	**0.10**	0.71
583			ursocholate	0.93	**0.03**	1.56
584		Purine Metabolism, (Hypo)Xanthine/Inosine containing	inosine	**0.57**	**0.34**	**1.75**
585			hypoxanthine	**1.26**	**0.59**	**1.21**
586			xanthine	**1.22**	**0.93**	**0.96**
587			xanthosine	1.15	**0.69**	**0.60**
588			2'-deoxyinosine	**1.76**	1.26	**0.59**
589			urate	1.47	0.80	0.98
590			uric acid ribonucleoside*	**4.35**	**3.99**	**0.52**
591			allantoin	**1.20**	1.10	**0.81**
592			allantoic acid	1.07	**0.41**	0.82
593		Purine Metabolism, Adenine containing	adenosine 5'-diphosphate (ADP)	**1.31**	**0.59**	**1.39**
594			adenosine 5'-monophosphate (AMP)	**1.57**	**0.66**	1.15
595			adenosine 3'-monophosphate (3'-AMP)	**2.29**	1.12	**0.68**
596			adenosine 2'-monophosphate (2'-AMP)	0.84	**0.83**	1.02
597			adenosine 3',5'-diphosphate	0.73	0.42	**1.54**
598			adenylosuccinate	1.20	1.27	**0.58**
599			adenosine	**0.67**	**0.44**	1.00
600			adenine	1.15	**1.87**	1.01
601			N1-methyladenosine	**1.89**	0.87	0.84
602			N6-carbamoylthreonyladenosine	**1.71**	**0.58**	0.86
603			2'-deoxyadenosine 5'-monophosphate	**2.35**	**0.28**	1.09
604			2'-deoxyadenosine 3'-monophosphate	**3.31**	**4.25**	**0.30**
605			N6-succinyladenosine	0.71	0.66	**0.69**
606		Purine Metabolism, Guanine containing	guanosine 5'- diphosphate (GDP)	1.30	**0.37**	1.07
607			guanosine 5'- monophosphate (5'-GMP)	0.89	**0.39**	**1.78**
608			guanosine	0.95	**0.69**	**1.65**
609			guanine	**1.51**	1.23	**0.63**
610			7-methylguanine	**1.48**	**0.53**	**1.19**
611			N2,N2-dimethylguanosine	**2.50**	**0.38**	1.22
612			2'-deoxyguanosine	**1.97**	**1.49**	0.76

613			guanosine 2'-monophosphate (2'-GMP)*	1.32	1.16	0.60
614		Pyrimidine Metabolism, Orotate containing	orotate	1.15	0.24	1.17
615			orotidine	1.17	1.01	0.97
616	Nucleotide	Pyrimidine Metabolism, Uracil containing	uridine 5'-monophosphate (UMP)	1.78	0.81	1.32
617			uridine 3'-monophosphate (3'-UMP)	1.41	1.01	0.62
618			uridine-2',3'-cyclic monophosphate	0.79	0.54	0.78
619			uridine	0.98	0.46	1.33
620			uracil	2.13	1.41	0.65
621			pseudouridine	2.07	0.80	0.62
622			5,6-dihydrouridine	1.51	0.82	0.66
623			2'-O-methyluridine	1.07	0.62	1.20
624			5-methyluridine (ribothymidine)	1.42	0.32	1.27
625			2'-deoxyuridine	1.52	0.68	0.73
626			3-ureidopropionate	0.96	353.07	0.61
627			beta-alanine	2.37	1.49	0.71
628		Pyrimidine Metabolism, Cytidine containing	cytidine diphosphate	0.86	0.31	1.46
629			cytidine 5'-monophosphate (5'-CMP)	0.95	0.44	1.27
630			cytidine	1.11	0.45	1.16
631			cytosine	1.24	2.15	1.11
632			3-methylcytidine	0.90	0.61	0.73
633			5-methylcytidine	1.20	0.27	0.97
634			2'-deoxycytidine 5'-monophosphate	2.23	0.91	0.48
635			2'-deoxycytidine	1.78	0.60	0.64
636			2'-O-methylcytidine	1.21	0.38	1.32
637			5-methyl-2'-deoxycytidine	2.08	0.45	0.39
638		Pyrimidine Metabolism, Thymine containing	thymidine	1.81	0.99	0.55
639			thymine	1.78	0.94	0.65
640			3-aminoisobutyrate	1.26	0.52	1.03
641		Purine and Pyrimidine Metabolism	methylphosphate	0.98	0.56	0.99
642		Dinucleotide	(3'-5')-adenylylcytidine	0.80	0.24	1.21
643			(3'-5')-adenylyluridine	1.19	0.18	1.43
644			(3'-5')-uridylyluridine	0.69	0.20	2.31
645			(3'-5')-adenylyladenosine*	0.71	0.22	1.46
646			(3'-5')-cytidylyluridine*	1.30	0.80	0.66
647			(3'-5')-guanylylcytidine	0.80	0.27	1.33
648			(3'-5')-uridylylcytidine*	0.81	0.39	1.28
649		Nicotinate and Nicotinamide Metabolism	quinolinate	0.94	2.10	1.03
650			nicotinate	1.05	0.83	0.99
651			nicotinate ribonucleoside	2.00	2.24	0.59
652			nicotinamide	0.96	0.84	1.07
653			nicotinamide adenine dinucleotide (NAD+)	1.19	0.51	1.07
654			1-methylnicotinamide	0.42	3.02	1.46
655			trigonelline (N'-methylnicotinate)	1.22	0.04	1.54
656			N1-Methyl-2-pyridone-5-carboxamide	1.35	1.41	1.07
657		Riboflavin Metabolism	riboflavin (Vitamin B2)	1.76	0.70	0.72
658			flavin adenine dinucleotide (FAD)	1.30	0.55	1.13
659			flavin mononucleotide (FMN)	1.31	0.58	0.92
660		Pantothenate and	pantothenate	1.08	0.58	0.98
661			phosphopantetheine	0.49	0.42	4.33

662	Cofactors and Vitamins	Pantothenate and CoA Metabolism	3'-dephosphocoenzyme A	**0.32**	1.25	**2.08**
663			coenzyme A	0.51	0.53	**3.63**
664			pantetheine	0.71	0.73	**1.57**
665		Ascorbate and Aldarate Metabolism	ascorbate (Vitamin C)	0.99	**1.99**	1.10
666			dehydroascorbate	0.96	0.89	1.09
667			threonate	1.22	0.82	1.38
668			oxalate (ethanedioate)	0.98	**0.72**	0.91
669			gulonate*	1.01	**0.43**	1.22
670		Tocopherol Metabolism	alpha-tocopherol	**1.18**	**2.56**	**0.87**
671			gamma-tocopherol/beta-tocopherol	**1.60**	**6.27**	1.03
672		Folate Metabolism	5-methyltetrahydrofolate (5MeTHF)	**1.99**	**1.51**	1.26
673		Tetrahydrobiopterin Metabolism	biopterin	**1.42**	**0.70**	0.97
674			dihydrobiopterin	1.13	**0.59**	0.95
675		Pterin Metabolism	pterin	**1.48**	**0.55**	0.97
676			xanthopterin	1.03	**0.46**	**0.81**
677		Hemoglobin and Porphyrin Metabolism	heme	2.53	1.97	0.46
678			bilirubin (Z,Z)	1.74	**3.39**	0.80
679			biliverdin	**1.92**	**1.57**	0.85
680		Thiamine Metabolism	thiamin (Vitamin B1)	**1.19**	**0.65**	1.00
681			thiamin monophosphate	**1.52**	**0.53**	0.93
682		Vitamin A Metabolism	retinol (Vitamin A)	1.91	0.36	0.97
683			retinal	1.33	**0.16**	**1.30**
684		Vitamin B6 Metabolism	pyridoxamine	**1.74**	0.76	**0.77**
685			pyridoxamine phosphate	1.13	**0.57**	0.88
686			pyridoxal phosphate	**1.71**	**0.34**	1.29
687			pyridoxal	0.91	**0.71**	1.00
688			pyridoxate	1.16	**0.73**	**0.81**
689	Xenobiotics	Benzoate Metabolism	hippurate	1.37	**0.38**	**0.40**
690			4-hydroxyhippurate	1.04	0.86	0.69
691			catechol sulfate	1.41	**0.33**	1.00
692			p-cresol sulfate	**4.02**	1.59	1.12
693			phenylpropionylglycine	1.13	**0.03**	1.00
694		Food Component/Plant	2,3-dihydroxyisovalerate	1.19	**0.32**	0.76
695			2,8-quinolinediol	0.98	**0.46**	1.00
696			gluconate	**0.44**	**0.47**	**1.33**
697			beta-guanidinopropanoate	1.03	**0.39**	0.94
698			cinnamoylglycine	**1.65**	**0.37**	1.00
699			enterolactone	**0.26**	**0.06**	1.00
700			ergothioneine	1.20	**0.01**	0.87
701			erythritol	1.12	1.11	0.93
702			kojibiose	**0.26**	**0.67**	0.84
703			N-glycolylneuraminate	**1.46**	0.82	0.90
704			stachydrine	**3.08**	**0.13**	0.83
705			tartarate	0.87	**16.59**	1.23
706			methyl glucopyranoside (alpha + beta)	**0.40**	**0.46**	1.07
707		Bacterial/Fungal	tartronate (hydroxymalonate)	0.99	**0.57**	0.87
708		Drug - Analgesics, Anesthetics	lidocaine	1.05	**3.57**	**0.28**
709			N-ethylglycinexylidide	1.00	1.00	1.00
710			sulfate*	1.03	**0.63**	1.03

711		Chemical	S-(3-hydroxypropyl)mercapturic acid (HPMA)	**2.16**	**0.23**	1.02
712			perfluorooctanesulfonate (PFOS)	0.87	**0.06**	1.17
713			3-hydroxypyridine sulfate	**1.36**	**0.16**	1.00
714			thioproline	0.93	0.54	1.90

Green: indicates significant difference ($p \leq 0.05$) between the groups shown, metabolite ratio of < 1.00

Light Green: narrowly missed statistical cutoff for significance 0.05<p<0.10, metabolite ratio of < 1.00

Red: indicates significant difference ($p \leq 0.05$) between the groups shown; metabolite ratio of ≥ 1.00

Light Red: narrowly missed statistical cutoff for significance 0.05<p<0.10, metabolite ratio of ≥ 1.00

D
AAV8.*Ucn2* vs Saline
1.09
1.16
0.81
0.91
1.06
**1.82**
1.04
0.94
1.00
1.16
1.07
0.97
1.06
1.45
1.04
1.17
**1.26**
1.13
1.03
1.43
1.06
**1.25**
**1.50**
1.10
1.40
0.85
**2.43**
1.05
**1.38**
1.30
**1.46**
1.88
**2.04**
0.90
0.79
0.78
1.22
**2.56**
1.25
**1.78**
1.14
1.03
1.38
1.19
1.03

0.96
1.22
2.26
**1.71**
1.13
**1.34**
0.70
1.02
**0.66**
1.04
1.00
0.98
0.91
0.72
**1.75**
**3.03**
1.15
1.03
**1.38**
1.35
1.51
1.75
1.20
0.98
**1.38**
1.01
1.05
1.76
**1.52**
1.00
1.23
**0.52**
1.34
**2.35**
0.93
0.99
1.09
**1.29**
1.14
1.20
1.04
0.75
0.74
1.27
1.01
1.04
**0.53**
1.32
**1.76**

1.15
1.07
1.07
0.97
0.36
0.93
0.79
0.82
**1.91**
1.17
0.76
1.86
0.93
**0.59**
1.20
1.01
0.95
1.09
0.86
1.23
0.95
1.42
1.05
**3.91**
0.99
1.09
1.48
1.94
1.10
**0.48**
0.86
1.17
1.51
1.09
0.98
1.33
**0.47**
**0.82**
1.03
**0.71**
0.89
**2.46**
5.15
**1.18**
**0.82**
1.05
1.05
1.10
0.66

1.66
1.43
1.52
1.14
1.30
1.43
0.76
1.27
1.94
1.19
1.57
1.20
1.20
1.78
1.99
1.66
1.24
1.22
1.74
1.00
2.07
1.88
2.54
1.94
1.19
1.12
1.42
1.44
0.78
1.23
1.32
0.92
0.94
0.94
1.76
1.57
1.29
2.01
1.01
1.07
0.82
0.76
1.69
0.87
2.40
1.90
1.42
1.04
1.29

2.43
1.13
1.24
1.22
1.75
0.77
1.18
0.69
1.33
1.40
1.14
1.96
0.93
0.94
1.00
2.42
1.18
1.17
1.37
1.02
1.16
1.09
0.69
0.43
0.96
1.07
1.11
1.22
0.93
0.91
0.32
0.50
0.62
0.58
0.35
0.47
0.35
0.56
0.52
0.58
0.35
0.59
0.39
0.52
0.46
0.45
0.65
0.56

0.44
0.41
0.48
0.53
0.47
0.59
0.44
0.54
0.57
0.41
0.42
0.39
0.35
0.35
0.33
0.43
0.45
1.02
1.57
0.93
1.00
1.62
1.19
1.50
0.76
0.82
0.96
0.89
1.04
1.38
1.01
1.09
0.91
0.97
0.72
1.11
1.67
2.67
0.95
1.56
1.50
1.37
1.41
1.26
0.53
1.00
0.92
1.02
1.07

0.83
0.84
**1.70**
**0.83**
1.10
**2.43**
0.86
**1.22**
0.84
0.65
**0.61**
0.86
1.25
1.02
1.09
**1.56**
**1.85**
**2.14**
**2.80**
**2.08**
**1.65**
0.92
**0.70**
0.58
1.09
0.90
0.87
**0.48**
0.90
1.12
0.71
1.23
1.37
1.37
1.22
**1.38**
0.95
**1.28**
0.92
**1.48**
0.97
**1.59**
1.12
0.89
**0.79**
0.92
1.18
**0.57**
**0.28**

0.47
0.19
0.36
0.52
0.53
0.45
1.12
0.78
1.07
1.31
1.23
0.65
0.90
0.76
0.73
0.97
1.16
0.97
0.93
0.98
0.94
0.72
1.04
0.88
1.18
1.21
1.00
1.06
1.14
1.18
0.88
1.19
1.04
1.18
0.95
1.06
1.66
2.43
1.42
0.74
0.77
0.82
1.24
1.03
1.06
0.87

1.33
1.11
1.03
1.23
0.99
0.93
1.83
1.46
1.09
1.26
0.96
1.21
1.62
1.10
1.41
1.66
1.16
1.02
1.01
0.47
0.93
1.05
0.84
0.53
1.26
0.42
0.98
1.07
0.50
0.93
1.21
0.47
0.51
1.25
0.33
0.33
0.46
0.14
0.43
0.38
0.47
0.31
0.64
0.49
0.88
1.01
1.10

0.91
1.08
1.03
1.17
1.16
0.67
0.87
0.78
0.78
2.56
0.68
0.52
0.63
0.53
0.42
0.65
1.03
2.47
1.62
2.11
2.04
1.95
0.99
0.60
0.76
1.48
1.61
2.64
1.62
1.65
1.20
0.32
0.58
0.58
0.65
0.89
0.65
0.73
0.71
0.80
0.57
0.72
0.88
0.78
1.00
1.74

**0.64**
0.72
1.03
1.07
1.22
0.95
1.13
**0.65**
**0.82**
0.89
1.06
1.27
**1.51**
1.70
**1.67**
**1.44**
**0.64**
1.03
**0.75**
0.95
1.38
1.35
**1.19**
**1.50**
0.92
0.88
1.16
0.91
**0.68**
**0.82**
**0.62**
0.88
0.97
**1.34**
0.90
0.92
1.11
1.08
1.01
1.01
0.95
0.85

1.81
1.23
0.98
1.27
1.00
0.77
0.67
1.20
1.24
1.33
1.33
1.04
1.09
1.49
1.05
1.15
1.33
1.66
1.16
1.26
1.36
1.35
1.23
1.55
1.46
1.45
1.70
1.64
1.39
1.55
1.21
1.03
1.21
0.79
1.03
0.85
1.06
1.05
0.90
1.15
1.00
1.10
1.24
0.74

0.93
0.84
**0.57**
0.83
**0.36**
**0.60**
1.08
**0.33**
1.06
0.93
0.93
**0.57**
0.88
**0.46**
0.97
1.25
**0.40**
0.78
1.07
**1.60**
**2.04**
**1.34**
1.04
1.17
0.66
0.97
1.39
**0.89**
**1.58**
**1.42**
**1.28**
1.03
1.16
**2.37**
0.83
**1.39**
0.90
1.13
**1.32**
1.40
**0.40**
**0.76**
**1.52**
1.13
**1.35**
**0.62**
**1.49**
**1.68**
0.76

0.91
1.01
1.01
0.96
0.82
1.55
1.20
0.89
0.91
0.87
**1.63**
**1.36**
1.00
**0.17**
1.15
**2.18**
**1.23**
**1.37**
**0.52**
1.01
1.26
**0.59**
0.90
**1.85**
0.57
**0.62**
0.86
1.18
1.06
**1.60**
**2.56**
**2.87**
**2.03**
1.02
1.54
**1.45**
0.97
**1.31**
0.89
**1.15**
**1.41**
**1.90**
1.33
**1.53**
1.12
**1.45**
1.25
1.13
**3.57**

1.60
**3.67**
**1.40**
1.19
1.06
**1.52**
0.87
1.15
**0.86**
**0.68**
**1.91**
1.13
**1.37**
1.23
0.84
0.59
**0.59**
0.82
**1.24**
**1.35**
**1.72**
**2.04**
0.95
**1.32**
1.15
0.97
0.84
0.60
0.79
1.00
**2.28**
1.00
1.23
1.00
**1.66**
1.00
1.00
1.00
**1.49**
1.16
0.99
1.07
0.92
2.79
**1.24**
0.99
**0.28**
1.00
1.22

**1.69**
**1.60**
1.00
1.80
