# Supplementary material for: Significant alteration of liver metabolites by AAV8.Urocortin 2 gene transfer in mice with insulin resistance
Source: PLoS One. 2019 Dec 2;14(12):e0224428. doi: 10.1371/journal.pone.0224428 (PMC6886859; doi:10.1371/journal.pone.0224428)
Supplement: S2 Table — (PDF) [file pone.0224428.s003.pdf]

Supplementary Table 2. AAV8.*UCn2* altered metabolites in CHOW-fed mice

Number	Super Pathway	Sub Pathway	Biochemical Name	AAV8.*Ucn2* vs Saline
1	Amino Acid	Glycine, Serine and Threonine Metabolism	glycine	1.24
2			N-acetylglycine	1.78
3			betaine	2.19
4			betaine aldehyde	2.40
5			serine	1.15
6			N-acetylserine	1.63
7			threonine	1.24
8			N-acetylthreonine	2.37
9		Alanine and Aspartate Metabolism	aspartate	1.47
10			N-acetylaspartate (NAA)	1.71
11		Glutamate Metabolism	N-acetylglutamine	0.77
12		Histidine Metabolism	histidine	2.08
13			formiminoglutamate	5.48
14			anserine	1.79
15		Lysine Metabolism	lysine	1.19
16			N2-acetyllysine	2.06
17			N6-acetyllysine	2.03
18			N6,N6,N6-trimethyllysine	1.50
19		Phenylalanine Metabolism	phenylalanine	1.18
20		Tryptophan Metabolism	C-glycosyltryptophan	1.49
21			kynurenine	3.74
22		Leucine, Isoleucine and Valine Metabolism	leucine	1.20
23			isovalerylglycine	1.66
24			3-methylcrotonylglycine	2.78
25			isoleucine	1.35
26			N-acetylisoleucine	1.34
27			2-methylbutyrylglycine	2.33
28			ethylmalonate	0.61
29			valine	1.32
30			N-acetylvaline	1.57
31		Methionine, Cysteine, SAM and Taurine Metabolism	methionine	1.31
32			N-acetylmethionine	1.25
33			S-methylcysteine	1.63
34			hypotaurine	2.55
35			taurine	0.55
36			taurocyamine	1.59
37		Urea cycle; Arginine and Proline Metabolism	urea	1.60
38			ornithine	1.19
39			proline	1.26
40			dimethylarginine (SDMA + ADMA)	1.33
41			N-acetylcitrulline	2.52
42			N-delta-acetylornithine	4.51
43			N-alpha-acetylornithine	1.98

44			N-monomethylarginine	1.21
45		Polyamine Metabolism	spermidine	0.72
46			(N(1) + N(8))-acetylspermidine	0.68
47		Guanidino and Acetamido Metabolism	4-guanidinobutanoate	1.63
48		Glutathione Metabolism	5-oxoproline	1.28
49			2-hydroxybutyrate/2-hydroxyisobutyrate	2.10
50	Peptide	Gamma-glutamyl Amino Acid	gamma-glutamylisoleucine*	1.69
51			gamma-glutamylleucine	1.54
52			gamma-glutamyl-epsilon-lysine	1.69
53			gamma-glutamylphenylalanine	1.59
54		Dipeptide	glycylvaline	1.56
55	Carbohydrate	Glycolysis, Gluconeogenesis, and Pyruvate Metabolism	1,5-anhydroglucitol (1,5-AG)	1.52
56			glucose	0.38
57			lactate	0.59
58		Pentose Metabolism	ribose	0.76
59			arabitol/xylitol	0.84
60			ribulose/xylulose	0.47
61			sedoheptulose	0.32
62			ribulonate/xylulonate*	0.54
63		Glycogen Metabolism	maltopentaose	0.09
64			maltotetraose	0.01
65			maltotriose	0.06
66			maltose	0.10
67		Fructose, Mannose and Galactose Metabolism	fructose	0.12
68			mannitol/sorbitol	0.32
69			mannose	0.37
70		Nucleotide Sugar	UDP-glucose/UDP-galactose	3.21
71		Aminosugar Metabolism	glucuronate	0.67
72			N-acetylneuraminate	1.45
73			N-acetylglucosamine/N-acetylgalactosamine	1.85
74		Advanced Glycation End-product	N6-carboxymethyllysine	1.39
75	Energy	TCA Cycle	fumarate	1.31
76			malate	1.20
77			itaconate	1.70
78		Medium Chain Fatty Acid	5-dodecenoate (12:1n7)	1.64
79		Long Chain Fatty Acid	myristate (14:0)	0.44
80			palmitoleate (16:1n7)	0.42
81			arachidate (20:0)	0.36
82			behenate (22:0)*	0.35
83			erucate (22:1n9)	0.36
84		Fatty Acid, Dicarboxylate	adipate (C6-DC)	1.52
85			tetradecanedioate (C14-DC)	1.61
86		Fatty Acid Metabolism (also BCAA Metabolism)	propionylcarnitine (C3)	1.69
87			propionylglycine	4.60
88		Fatty Acid Metabolism(Acyl Glycine)	valerylglycine	2.61
89		Fatty Acid Metabolism(Acyl Carnitine)	palmitoylcarnitine (C16)	2.06
90			linoleoylcarnitine (C18:2)*	1.79

91			oleoylcarnitine (C18:1)	1.57
92		Ketone Bodies	3-hydroxybutyrate (BHBA)	2.12
93		Fatty Acid Metabolism (Acyl Choline)	palmitoylcholine	4.52
94			oleoylcholine	2.73
95			palmitoloelycholine	2.85
96			linoleoylcholine*	4.40
97			stearoylcholine*	3.92
98		Fatty Acid, Monohydroxy	4-HDoHE	0.36
99		Eicosanoid	5-HETE	0.31
100			12-HETE	0.69
101		Endocannabinoid	stearoyl ethanolamide	1.26
102			N-arachidonoyltaurine	0.40
103			N-oleoyltaurine	0.24
104			N-stearoyltaurine	0.48
105			N-palmitoyltaurine	0.16
106			N-palmitoleoyltaurine*	0.19
107			N-linoleoyltaurine*	0.32
108			N-linolenoyltaurine*	0.34
109		Inositol Metabolism	inositol 1-phosphate (I1P)	2.15
110		Phospholipid Metabolism	phosphoethanolamine	1.28
111			glycerophosphoinositol*	1.41
112		Phosphatidylcholine (PC)	1,2-dipalmitoyl-GPC (16:0/16:0)	1.26
113			1-palmitoyl-2-stearoyl-GPC (16:0/18:0)	1.25
114			1-palmitoyl-2-oleoyl-GPC (16:0/18:1)	1.08
115			1-palmitoyl-2-linoleoyl-GPC (16:0/18:2)	1.12
116			1-palmitoyl-2-docosahexaenoyl-GPC (16:0/22:6)	1.09
117			1,2-dilinoleoyl-GPC (18:2/18:2)	1.29
118			1-linoleoyl-2-linolenoyl-GPC (18:2/18:3)*	1.56
119		Phosphatidylethanolamine (PE)	1,2-dipalmitoyl-GPE (16:0/16:0)*	1.31
120			1-palmitoyl-2-stearoyl-GPE (16:0/18:0)*	1.43
121			1-stearoyl-2-oleoyl-GPE (18:0/18:1)	1.19
122		Phosphatidylserine (PS)	1-stearoyl-2-oleoyl-GPS (18:0/18:1)	1.28
123	Lipid	Phosphatidylinositol (PI)	1-palmitoyl-2-linoleoyl-GPI (16:0/18:2)	1.51
124			1-stearoyl-2-linoleoyl-GPI (18:0/18:2)	1.61
125			1-oleoyl-2-linoleoyl-GPI (18:1/18:2)*	1.38
126			1-stearoyl-2-arachidonoyl-GPI (18:0/20:4)	1.07
127			1-oleoyl-2-arachidonoyl-GPI (18:1/20:4) *	1.29
128		Plasmalogen	1-(1-enyl-palmitoyl)-2-palmitoyl-GPC (P-16:0/16:0)*	1.26
129			1-(1-enyl-palmitoyl)-2-arachidonoyl-GPE (P-16:0/20:4)*	1.28
130			1-(1-enyl-stearoyl)-2-arachidonoyl-GPE (P-18:0/20:4)*	1.27
131		Monoacylglycerol	1-myristoylglycerol (14:0)	0.29
132			1-palmitoleoylglycerol (16:1)*	0.28
133			2-palmitoleoylglycerol (16:1)*	0.23
134			2-arachidonoylglycerol (20:4)	0.54
135			diacylglycerol (12:0/18:1, 14:0/16:1, 16:0/14:1) [2]*	0.64
136			diacylglycerol (14:0/18:1, 16:0/16:1) [1]*	0.65
137			diacylglycerol (14:0/18:1, 16:0/16:1) [2]*	0.67

138		Diacylglycerol	palmitoleoyl-palmitoleoyl-glycerol (16:1/16:1) [2]*	0.62
139			palmitoleoyl-oleoyl-glycerol (16:1/18:1) [2]*	0.57
140			palmitoyl-arachidonoyl-glycerol (16:0/20:4) [1]*	1.70
141			palmitoyl-arachidonoyl-glycerol (16:0/20:4) [2]*	1.20
142			stearoyl-arachidonoyl-glycerol (18:0/20:4) [1]*	1.83
143			oleoyl-arachidonoyl-glycerol (18:1/20:4) [1]*	3.52
144			stearoyl-docosahexaenoyl-glycerol (18:0/22:6) [2]*	1.34
145		Sphingolipid Synthesis	sphinganine	2.09
146		Dihydroceramides	N-palmitoyl-sphinganine (d18:0/16:0)	1.42
147			N-stearoyl-sphinganine (d18:0/18:0)*	1.70
148		Ceramides	N-palmitoyl-sphingosine (d18:1/16:0)	1.42
149			N-palmitoyl-heptadecasphingosine (d17:1/16:0)*	1.44
150			ceramide (d18:1/17:0, d17:1/18:0)*	1.35
151			ceramide (d16:1/24:1, d18:1/22:1)*	0.82
152			ceramide (d18:2/24:1, d18:1/24:2)*	1.29
153		Hexosylceramides (HCER)	glycosyl-N-palmitoyl-sphingosine (d18:1/16:0)	1.50
154			glycosyl ceramide (d18:1/23:1, d17:1/24:1)*	1.93
155			glycosyl ceramide (d18:2/24:1, d18:1/24:2)*	1.79
156		Lactosylceramides (LCER)	lactosyl-N-palmitoyl-sphingosine (d18:1/16:0)	1.60
157			lactosyl-N-nervonoyl-sphingosine (d18:1/24:1)*	1.72
158		Dihydrosphingomyelins	palmitoyl dihydrosphingomyelin (d18:0/16:0)*	1.40
159			sphingomyelin (d18:0/18:0, d19:0/17:0)*	1.48
160		Sphingomyelins	tricosanoyl sphingomyelin (d18:1/23:0)*	1.25
161			sphingomyelin (d18:2/23:1)*	1.39
162			sphingomyelin (d18:2/24:2)*	1.81
163			sphingomyelin (d18:1/17:0, d17:1/18:0, d19:1/16:0)	1.19
164			sphingomyelin (d18:1/24:1, d18:2/24:0)*	1.29
165			sphingomyelin (d18:2/24:1, d18:1/24:2)*	1.35
166		Sphingosines	sphingosine	1.22
167		Sterol	cholesterol	1.27
168			beta-sitosterol	1.50
169		Secondary Bile Acid Metabolism	deoxycholate	1.82
170	Nucleotide	Purine Metabolism, (Hypo)Xanthine/Inosine containing	inosine	0.57
171			hypoxanthine	1.26
172			xanthine	1.22
173			2'-deoxyinosine	1.76
174			uric acid ribonucleoside*	4.35
175			allantoin	1.20
176		Purine Metabolism, Adenine containing	adenosine 5'-monophosphate (AMP)	1.57
177			adenosine 3'-monophosphate (3'-AMP)	2.29
178			adenosine	0.67
179			N1-methyladenosine	1.89
180			N6-carbamoylthreonyladenosine	1.71
181			2'-deoxyadenosine 5'-monophosphate	2.35
182			2'-deoxyadenosine 3'-monophosphate	3.31
183		Purine Metabolism, Guanine containing	guanine	1.51
184			2'-deoxyguanosine	1.97

185		Pyrimidine Metabolism, Uracil containing	uridine 5'-monophosphate (UMP)	1.78
186			uridine 3'-monophosphate (3'-UMP)	1.41
187			uracil	2.13
188			pseudouridine	2.07
189			5-methyluridine (ribothymidine)	1.42
190			beta-alanine	2.37
191		Pyrimidine Metabolism, Cytidine containing	2'-deoxycytidine 5'-monophosphate	2.23
192		Pyrimidine Metabolism, Thymine containing	thymidine	1.81
193			thymine	1.78
194	Cofactors and Vitamins	Nicotinate and Nicotinamide Metabolism	nicotinamide adenine dinucleotide (NAD+)	1.19
195			1-methylnicotinamide	0.42
196			N1-Methyl-2-pyridone-5-carboxamide	1.35
197		Riboflavin Metabolism	riboflavin (Vitamin B2)	1.76
198			flavin adenine dinucleotide (FAD)	1.30
199			flavin mononucleotide (FMN)	1.31
200		Tocopherol Metabolism	gamma-tocopherol/beta-tocopherol	1.60
201		Folate Metabolism	5-methyltetrahydrofolate (5MeTHF)	1.99
202		Tetrahydrobiopterin Metabolism	biopterin	1.42
203		Pterin Metabolism	pterin	1.48
204		Hemoglobin and Porphyrin Metabolism	biliverdin	1.92
205		Thiamine Metabolism	thiamin monophosphate	1.52
206		Vitamin B6 Metabolism	pyridoxamine	1.74
207	Xenobiotics	Benzoate Metabolism	p-cresol sulfate	4.02
208		Food Component/Plant	enterolactone	0.26
209			kojibiose	0.26
210			stachydrine	3.08
211			methyl glucopyranoside (alpha + beta)	0.40
212		Chemical	S-(3-hydroxypropyl)mercapturic acid (HPMA)	2.16

Green: indicates significant difference ($p \leq 0.05$) between the groups shown, metabolite ratio of < 1.00

Red: indicates significant difference ($p \leq 0.05$) between the groups shown; metabolite ratio of ≥ 1.00
