# Supplementary material for: Significant alteration of liver metabolites by AAV8.Urocortin 2 gene transfer in mice with insulin resistance
Source: PLoS One. 2019 Dec 2;14(12):e0224428. doi: 10.1371/journal.pone.0224428 (PMC6886859; doi:10.1371/journal.pone.0224428)
Supplement: S3 Table — (PDF) [file pone.0224428.s004.pdf]

	Supplementary Table 3. HFD altered metabolites in Liver (HFD-saline vs CHOW-saline)			
Number	Super Pathway	Sub Pathway	Biochemical Name	HFD-Saline vs CHOW-Saline
1		Glycine, Serine and Threonine Metabolism	glycine	0.63
2			N-acetylglycine	0.38
3			betaine	1.64
4			betaine aldehyde	0.25
5			serine	0.61
6			N-acetylserine	0.70
7			threonine	0.63
8			N-acetylthreonine	0.59
9		Alanine and Aspartate Metabolism	alanine	0.73
10			N-acetylalanine	0.71
11			aspartate	0.61
12			N-acetylaspartate (NAA)	0.43
13			asparagine	0.81
14			N-acetylasparagine	0.56
15		Glutamate Metabolism	glutamate	0.46
16			glutamine	1.84
17			N-acetylglutamine	0.70
18			gamma-carboxyglutamate	0.69
19			glutamate, gamma-methyl ester	0.53
20			pyroglutamine*	0.48
21			carboxyethyl-GABA	0.49
22			N-methyl-GABA	0.57
23		Histidine Metabolism	histidine	0.75
24			1-methylhistidine	0.66
25			3-methylhistidine	0.52
26			N-acetylhistidine	0.48
27			N-acetyl-3-methylhistidine*	0.30
28			N-acetyl-1-methylhistidine*	0.24
29			formiminoglutamate	0.25
30			anserine	0.54
31			1-methylhistamine	0.25
32			1-ribosyl-imidazoleacetate*	0.11
33			4-imidazoleacetate	0.34
34		Lysine Metabolism	lysine	0.72
35			N2-acetyllysine	0.35
36			N6-acetyllysine	0.47
37			N6,N6,N6-trimethyllysine	0.70
38			5-(galactosylhydroxy)-L-lysine	1.59
39			saccharopine	1.82
40			pipecolate	0.62
41			5-aminovalerate	0.52
42			N-trimethyl 5-aminovalerate	0.60
43		Phenylalanine Metabolism	phenylalanine	0.68

44	Amino Acid	Tyrosine Metabolism	tyrosine	0.69
45			phenol sulfate	0.27
46			O-methyltyrosine	0.23
47			N-formylphenylalanine	0.46
48		Tryptophan Metabolism	tryptophan	0.73
49			C-glycosyltryptophan	0.60
50			kynurenine	0.45
51			kynurenate	0.39
52			5-hydroxyindoleacetate	0.31
53			indolelactate	0.26
54			indole-3-carboxylate	0.56
55			indoleacetylglycine	0.09
56			3-indoxyl sulfate	0.35
57		Leucine, Isoleucine and Valine Metabolism	leucine	0.70
58			4-methyl-2-oxopentanoate	4.11
59			isovalerylglycine	0.26
60			3-methylcrotonylglycine	0.18
61			isoleucine	0.76
62			valine	0.68
63			N-acetylvaline	0.59
64			3-methyl-2-oxobutyrate	6.10
65			isobutyrylcarnitine (C4)	0.18
66			3-hydroxyisobutyrate	0.64
67		Methionine, Cysteine, SAM and Taurine Metabolism	methionine	0.65
68			N-formylmethionine	0.44
69			S-methylmethionine	0.14
70			methionine sulfoxide	0.81
71			S-adenosylmethionine (SAM)	3.59
72			S-adenosylhomocysteine (SAH)	0.34
73			alpha-ketobutyrate	1.89
74			hypotaurine	0.50
75		Urea cycle; Arginine and Proline Metabolism	arginine	1.38
76			urea	0.67
77			ornithine	0.82
78			citrulline	0.64
79			homocitrulline	0.12
80			proline	0.70
81			dimethylarginine (SDMA + ADMA)	0.54
82			N-acetylarginine	0.39
83			N-acetylcitrulline	0.22
84			trans-4-hydroxyproline	0.50
85			N-monomethylarginine	0.56
86			argininate*	1.50
87		Creatine Metabolism	guanidinoacetate	0.53
88		Polyamine Metabolism	putrescine	14.55
89			spermidine	1.31
90			5-methylthioadenosine (MTA)	2.29

91			(N(1) + N(8))-acetylspermidine	1.64
92		Guanidino and Acetamido Metabolism	4-guanidinobutanoate	0.14
93		Glutathione Metabolism	glutathione, oxidized (GSSG)	0.68
94			S-methylglutathione	1.83
95			4-hydroxy-nonenal-glutathione	0.38
96	Peptide	Gamma-glutamyl Amino Acid	gamma-glutamylglutamate	0.37
97			gamma-glutamylglycine	0.14
98			gamma-glutamylisoleucine*	0.57
99			gamma-glutamylleucine	0.51
100			gamma-glutamyl-epsilon-lysine	2.71
101			gamma-glutamylphenylalanine	0.46
102			gamma-glutamylthreonine	0.17
103			gamma-glutamylvaline	0.49
104		Dipeptide	glycylisoleucine	0.55
105			glycylleucine	0.55
106			glycylvaline	0.57
107			isoleucylglycine	0.55
108			leucylglycine	0.53
109			prolylglycine	0.41
110		Acetylated Peptides	phenylacetylglycine	0.32
111	Carbohydrate	Glycolysis, Gluconeogenesis, and Pyruvate Metabolism	1,5-anhydroglucitol (1,5-AG)	0.29
112			glucose	0.71
113			glucose 6-phosphate	0.56
114		Pentose Phosphate Pathway	sedoheptulose-7-phosphate	0.57
115		Pentose Metabolism	ribose	0.69
116			ribitol	0.52
117			sedoheptulose	0.37
118			ribulonate/xylulonate*	0.56
119		Glycogen Metabolism	maltose	0.64
120		Fructose, Mannose and Galactose Metabolism	fructose	0.33
121			mannitol/sorbitol	0.56
122			mannose	0.51
123			galactose 1-phosphate	0.58
124			2-ketogulonate	0.50
125			galactonate	0.31
126		Aminosugar Metabolism	glucuronate	0.68
127			N-acetylglucosamine/N-acetylgalactosamine	0.70
128		Advanced Glycation End-product	N6-carboxymethyllysine	0.14
129	Energy	TCA Cycle	succinate	1.58
130			2-methylcitrate/homocitrate	0.66
131		Oxidative Phosphorylation	phosphate	0.80
132		Fatty Acid Synthesis	malonylcarnitine	0.46
133		Medium Chain Fatty Acid	5-dodecenoate (12:1n7)	0.57
134			myristate (14:0)	2.62
135			myristoleate (14:1n5)	1.71
136			palmitate (16:0)	1.31
137			palmitoleate (16:1n7)	2.01

138	Long Chain Fatty Acid	10-heptadecenoate (17:1n7)	2.30
139		oleate/vaccenate (18:1)	1.69
140		nonadecanoate (19:0)	0.61
141		10-nonadecenoate (19:1n9)	1.84
142		eicosenoate (20:1)	2.17
143	Polyunsaturated Fatty Acid (n3 and n6)	eicosapentaenoate (EPA; 20:5n3)	0.62
144		docosahexaenoate (DHA; 22:6n3)	1.51
145		adrenate (22:4n6)	1.79
146		docosapentaenoate (n6 DPA; 22:5n6)	1.89
147		dihomo-linoleate (20:2n6)	1.98
148		mead acid (20:3n9)	2.99
149		docosatrienoate (22:3n6)*	4.64
150	Fatty Acid, Dicarboxylate	3-methylglutarate/2-methylglutarate	0.61
151		2-hydroxyglutarate	1.35
152		2-hydroxyadipate	0.57
153		3-hydroxyadipate*	0.46
154		3-methyladipate	0.42
155		pimelate (C7-DC)	0.69
156		azelate (C9-DC)	0.66
157		hexadecenedioate (C16:1-DC)*	1.88
158		octadecanedioate (C18-DC)	0.38
159	Fatty Acid, Amino	2-aminooctanoate	0.50
160	Fatty Acid Metabolism (also BCAA Metabolism)	butyrylglycine	0.47
161		propionylcarnitine (C3)	0.54
162		propionylglycine	0.23
163		methylmalonate (MMA)	1.40
164	Fatty Acid Metabolism(Acyl Glycine)	isocaproylglycine	0.20
165		hexanoylglycine	0.17
166		3,4-methylene heptanoylglycine	0.37
167		N-octanoylglycine	0.04
168		N-palmitoylglycine	0.33
169		N-linoleoylglycine	0.25
170	Fatty Acid Metabolism(Acyl Carnitine)	3-hydroxybutyrylcarnitine (2)	1.82
171		palmitoylcarnitine (C16)	2.33
172		linoleoylcarnitine (C18:2)*	0.61
173		oleoylcarnitine (C18:1)	2.76
174		pimeloylcarnitine/3-methyladipoylcarnitine (C7-DC)	0.27
175		arachidoylcarnitine (C20)*	1.39
176		eicosenoylcarnitine (C20:1)*	2.82
177		erucoylcarnitine (C22:1)*	1.84
178	Carnitine Metabolism	deoxycarnitine	0.32
179	Fatty Acid Metabolism (Acyl Choline)	palmitoloelycholine	0.57
180		linoleoylcholine*	0.30
181	Fatty Acid, Monohydroxy	4-HDoHE	0.33
182		13-HODE + 9-HODE	0.32
183		12,13-DiHOME	0.61
184		9,10-DiHOME	0.42

185	Fatty Acid, Dihydroxy	19,20-DiHDPA	1.95
186		5,6-DiHETrE	0.52
187		14,15-DiHETrE	0.62
188	Fatty Acid, Oxidized	4-hydroxy-2-nonenal	0.28
189	Eicosanoid	prostaglandin F2alpha	0.66
190		5-HETE	0.25
191		12-HETE	0.79
192		15-HETE	0.35
193	Endocannabinoid	N-arachidonoyltaurine	0.63
194		N-stearoyltaurine	1.56
195		N-linoleoyltaurine*	0.35
196		N-linolenoyltaurine*	0.15
197		linoleoyl ethanolamide	0.35
198	Inositol Metabolism	myo-inositol	0.62
199	Phospholipid Metabolism	glycerophosphorylcholine (GPC)	2.73
200		phosphoethanolamine	1.29
201		cytidine-5'-diphosphoethanolamine	0.44
202		glycerophosphoethanolamine	1.61
203	Phosphatidylcholine (PC)	1-myristoyl-2-palmitoyl-GPC (14:0/16:0)	0.81
204		1-myristoyl-2-arachidonoyl-GPC (14:0/20:4)*	0.70
205		1,2-dipalmitoyl-GPC (16:0/16:0)	0.89
206		1-palmitoyl-2-palmitoleoyl-GPC (16:0/16:1)*	0.78
207		1-palmitoyl-2-stearoyl-GPC (16:0/18:0)	1.12
208		1-palmitoyl-2-linoleoyl-GPC (16:0/18:2)	0.62
209		1-palmitoyl-2-gamma-linolenoyl-GPC (16:0/18:3n6)*	0.58
210		1-palmitoyl-2-dihomo-linolenoyl-GPC (16:0/20:3n3 or 6)*	1.15
211		1-palmitoyl-2-docosahexaenoyl-GPC (16:0/22:6)	0.82
212		1-palmitoleoyl-2-linoleoyl-GPC (16:1/18:2)*	0.32
213		1-stearoyl-2-oleoyl-GPC (18:0/18:1)	1.42
214		1-stearoyl-2-linoleoyl-GPC (18:0/18:2)*	0.83
215		1-stearoyl-2-arachidonoyl-GPC (18:0/20:4)	1.12
216		1-oleoyl-2-linoleoyl-GPC (18:1/18:2)*	0.69
217		1-oleoyl-2-docosahexaenoyl-GPC (18:1/22:6)*	0.74
218		1,2-dilinoleoyl-GPC (18:2/18:2)	0.28
219		1-linoleoyl-2-linolenoyl-GPC (18:2/18:3)*	0.06
220		1-linoleoyl-2-arachidonoyl-GPC (18:2/20:4n6)*	0.51
221	Phosphatidylethanolamine (PE)	1-palmitoyl-2-oleoyl-GPE (16:0/18:1)	0.75
222		1-palmitoyl-2-linoleoyl-GPE (16:0/18:2)	0.21
223		1-palmitoyl-2-arachidonoyl-GPE (16:0/20:4)*	0.63
224		1-palmitoyl-2-docosahexaenoyl-GPE (16:0/22:6)*	0.70
225		1-stearoyl-2-linoleoyl-GPE (18:0/18:2)*	0.51
226		1-stearoyl-2-arachidonoyl-GPE (18:0/20:4)	0.76
227		1-stearoyl-2-docosahexaenoyl-GPE (18:0/22:6)*	0.69
228		1-oleoyl-2-linoleoyl-GPE (18:1/18:2)*	0.33
229		1-oleoyl-2-arachidonoyl-GPE (18:1/20:4)*	0.74
230		1-oleoyl-2-docosahexaenoyl-GPE (18:1/22:6)*	0.73
231		1,2-dilinoleoyl-GPE (18:2/18:2)*	0.09

232	Lipid		1-linoleoyl-2-arachidonoyl-GPE (18:2/20:4)*	0.28
233		Phosphatidylserine (PS)	1-stearoyl-2-oleoyl-GPS (18:0/18:1)	0.84
234			1-stearoyl-2-arachidonoyl-GPS (18:0/20:4)	0.68
235		Phosphatidylglycerol (PG)	1-palmitoyl-2-oleoyl-GPG (16:0/18:1)	0.77
236			1-palmitoyl-2-linoleoyl-GPG (16:0/18:2)	0.57
237		Phosphatidylinositol (PI)	1-palmitoyl-2-linoleoyl-GPI (16:0/18:2)	0.15
238			1-palmitoyl-2-arachidonoyl-GPI (16:0/20:4)*	0.58
239			1-stearoyl-2-linoleoyl-GPI (18:0/18:2)	0.24
240			1-oleoyl-2-linoleoyl-GPI (18:1/18:2)*	0.12
241			1-stearoyl-2-arachidonoyl-GPI (18:0/20:4)	0.71
242			1-oleoyl-2-arachidonoyl-GPI (18:1/20:4) *	0.69
243		Lysophospholipid	1-palmitoleoyl-GPC (16:1)*	0.57
244			1-stearoyl-GPC (18:0)	1.23
245			1-linoleoyl-GPC (18:2)	0.60
246			1-linolenoyl-GPC (18:3)*	0.28
247			1-palmitoyl-GPE (16:0)	0.55
248			1-stearoyl-GPE (18:0)	0.77
249			1-oleoyl-GPE (18:1)	0.70
250			1-linoleoyl-GPE (18:2)*	0.30
251			1-arachidonoyl-GPE (20:4n6)*	0.73
252			1-palmitoyl-GPS (16:0)*	0.54
253			1-stearoyl-GPS (18:0)*	0.66
254			1-oleoyl-GPG (18:1)*	4.14
255			1-palmitoyl-GPI (16:0)	0.63
256			1-linoleoyl-GPI (18:2)*	0.18
257		Plasmalogen	1-(1-enyl-palmitoyl)-2-linoleoyl-GPE (P-16:0/18:2)*	0.32
258			1-(1-enyl-palmitoyl)-2-palmitoyl-GPC (P-16:0/16:0)*	0.47
259			1-(1-enyl-palmitoyl)-2-arachidonoyl-GPE (P-16:0/20:4)*	0.77
260			1-(1-enyl-palmitoyl)-2-oleoyl-GPC (P-16:0/18:1)*	0.55
261			1-(1-enyl-stearoyl)-2-linoleoyl-GPE (P-18:0/18:2)*	0.47
262		Glycerolipid Metabolism	glycerol	1.97
263			glycerol 3-phosphate	0.16
264			glycerophosphoglycerol	2.65
265		Monoacylglycerol	1-palmitoylglycerol (16:0)	2.27
266			1-stearoylglycerol (18:0)	3.06
267			1-oleoylglycerol (18:1)	1.56
268			1-dihomo-linolenylglycerol (20:3)	0.66
269			1-arachidonylglycerol (20:4)	0.42
270			1-docosahexaenoylglycerol (22:6)	1.24
271			2-linoleoylglycerol (18:2)	0.30
272			2-arachidonoylglycerol (20:4)	0.28
273			diacylglycerol (12:0/18:1, 14:0/16:1, 16:0/14:1) [1]*	8.05
274			diacylglycerol (12:0/18:1, 14:0/16:1, 16:0/14:1) [2]*	2.28
275			diacylglycerol (14:0/18:1, 16:0/16:1) [1]*	3.43
276			diacylglycerol (14:0/18:1, 16:0/16:1) [2]*	2.11
277			diacylglycerol (16:1/18:2 [2], 16:0/18:3 [1])*	0.75
278			palmitoyl-myristoyl-glycerol (16:0/14:0) [1]*	3.12

279		palmitoyl-myristoyl-glycerol (16:0/14:0) [2]	1.94
280		palmitoyl-palmitoyl-glycerol (16:0/16:0) [1]*	3.00
281		palmitoleoyl-palmitoleoyl-glycerol (16:1/16:1) [2]*	1.85
282		palmitoyl-oleoyl-glycerol (16:0/18:1) [1]*	2.85
283		palmitoyl-oleoyl-glycerol (16:0/18:1) [2]*	1.59
284		palmitoyl-linoleoyl-glycerol (16:0/18:2) [1]*	1.73
285		palmitoyl-linolenoyl-glycerol (16:0/18:3) [2]*	0.39
286		palmitoleoyl-oleoyl-glycerol (16:1/18:1) [2]*	1.75
287		palmitoyl-arachidonoyl-glycerol (16:0/20:4) [1]*	1.80
288	Diacylglycerol	palmitoleoyl-arachidonoyl-glycerol (16:1/20:4) [2]*	0.60
289		palmitoyl-docosahexaenoyl-glycerol (16:0/22:6) [2]*	2.70
290		oleoyl-oleoyl-glycerol (18:1/18:1) [1]*	2.24
291		oleoyl-oleoyl-glycerol (18:1/18:1) [2]*	1.42
292		oleoyl-linoleoyl-glycerol (18:1/18:2) [2]	0.70
293		oleoyl-linolenoyl-glycerol (18:1/18:3) [2]*	0.08
294		linoleoyl-linoleoyl-glycerol (18:2/18:2) [1]*	0.35
295		linoleoyl-linoleoyl-glycerol (18:2/18:2) [2]*	0.20
296		linoleoyl-linolenoyl-glycerol (18:2/18:3) [1]*	0.26
297		linoleoyl-linolenoyl-glycerol (18:2/18:3) [2]*	0.31
298		stearoyl-arachidonoyl-glycerol (18:0/20:4) [2]*	0.50
299		oleoyl-arachidonoyl-glycerol (18:1/20:4) [1]*	7.25
300		linoleoyl-arachidonoyl-glycerol (18:2/20:4) [2]*	0.47
301		stearoyl-docosahexaenoyl-glycerol (18:0/22:6) [2]*	0.67
302		linoleoyl-docosahexaenoyl-glycerol (18:2/22:6) [2]*	0.33
303	Sphingolipid Synthesis	sphinganine	2.33
304	Dihydroceramides	N-palmitoyl-sphinganine (d18:0/16:0)	1.53
305		N-stearoyl-sphinganine (d18:0/18:0)*	5.22
306		N-palmitoyl-sphingosine (d18:1/16:0)	1.53
307		N-(2-hydroxypalmitoyl)-sphingosine (d18:1/16:0(2OH))	2.55
308		N-stearoyl-sphingosine (d18:1/18:0)*	2.37
309		N-palmitoyl-sphingadienine (d18:2/16:0)*	1.26
310		N-behenoyl-sphingadienine (d18:2/22:0)*	0.44
311	Ceramides	N-palmitoyl-heptadecasphingosine (d17:1/16:0)*	1.64
312		ceramide (d18:1/14:0, d16:1/16:0)*	3.04
313		ceramide (d18:1/17:0, d17:1/18:0)*	1.74
314		ceramide (d18:1/20:0, d16:1/22:0, d20:1/18:0)*	1.29
315		ceramide (d16:1/24:1, d18:1/22:1)*	0.76
316		ceramide (d18:2/24:1, d18:1/24:2)*	0.75
317		glycosyl-N-stearoyl-sphingosine (d18:1/18:0)	2.32
318		glycosyl-N-behenoyl-sphingadienine (d18:2/22:0)*	0.36
319	Hexosylceramides (HCER)	glycosyl ceramide (d16:1/24:1, d18:1/22:1)*	0.60
320		glycosyl ceramide (d18:1/23:1, d17:1/24:1)*	0.35
321		glycosyl ceramide (d18:2/24:1, d18:1/24:2)*	0.63
322		lactosyl-N-palmitoyl-sphingosine (d18:1/16:0)	1.89
323		palmitoyl sphingomyelin (d18:1/16:0)	1.18
324		stearoyl sphingomyelin (d18:1/18:0)	1.34
325		behenoyl sphingomyelin (d18:1/22:0)*	0.29

326			tricosanoyl sphingomyelin (d18:1/23:0)*	0.61
327			lignoceroyl sphingomyelin (d18:1/24:0)	0.39
328			sphingomyelin (d18:2/23:1)*	0.31
329			sphingomyelin (d18:2/24:2)*	0.27
330			sphingomyelin (d18:1/14:0, d16:1/16:0)*	1.43
331			sphingomyelin (d18:2/16:0, d18:1/16:1)*	0.47
332		Sphingomyelins	sphingomyelin (d18:1/19:0, d19:1/18:0)*	0.56
333			sphingomyelin (d18:1/20:0, d16:1/22:0)*	0.65
334			sphingomyelin (d18:1/20:1, d18:2/20:0)*	0.48
335			sphingomyelin (d18:1/21:0, d17:1/22:0, d16:1/23:0)*	0.29
336			sphingomyelin (d18:2/21:0, d16:2/23:0)*	0.23
337			sphingomyelin (d18:1/22:1, d18:2/22:0, d16:1/24:1)*	0.32
338			sphingomyelin (d18:1/22:2, d18:2/22:1, d16:1/24:2)*	0.37
339			sphingomyelin (d18:2/23:0, d18:1/23:1, d17:1/24:1)*	0.39
340			sphingomyelin (d18:1/24:1, d18:2/24:0)*	0.49
341			sphingomyelin (d18:2/24:1, d18:1/24:2)*	0.45
342			sphingosine	1.64
343		Sphingosines	hexadecasphingosine (d16:1)*	2.78
344			heptadecasphingosine (d17:1)	1.35
345		Mevalonate Metabolism	3-hydroxy-3-methylglutarate	0.57
346			4-cholesten-3-one	5.18
347		Sterol	beta-sitosterol	0.50
348			campesterol	0.04
349		Primary Bile Acid Metabolism	cholate	0.40
350			deoxycholate	0.21
351			taurodeoxycholate	0.22
352			6-beta-hydroxylithocholate	0.18
353			taurolithocholate	0.18
354		Secondary Bile Acid Metabolism	6-oxolithocholate	0.06
355			hyodeoxycholate	0.08
356			7-ketodeoxycholate	0.10
357			ursocholate	0.03
358			inosine	0.34
359			hypoxanthine	0.59
360		Purine Metabolism, (Hypo)Xanthine/Inosine containing	xanthine	0.93
361			xanthosine	0.69
362			uric acid ribonucleoside*	3.99
363			allantoic acid	0.41
364			adenosine 5'-diphosphate (ADP)	0.59
365			adenosine 5'-monophosphate (AMP)	0.66
366			adenosine	0.44
367		Purine Metabolism, Adenine containing	adenine	1.87
368			N6-carbamoylthreonyladenosine	0.58
369			2'-deoxyadenosine 5'-monophosphate	0.28
370			2'-deoxyadenosine 3'-monophosphate	4.25
371			guanosine 5'- diphosphate (GDP)	0.37
372			guanosine	0.69

373	Nucleotide	Purine Metabolism, Guanine containing	7-methylguanine	0.53
374			N2,N2-dimethylguanosine	0.38
375			2'-deoxyguanosine	1.49
376		Pyrimidine Metabolism, Orotate containing	orotate	0.24
377		Pyrimidine Metabolism, Uracil containing	uridine-2',3'-cyclic monophosphate	0.54
378			uridine	0.46
379			uracil	1.41
380			2'-O-methyluridine	0.62
381			5-methyluridine (ribothymidine)	0.32
382			2'-deoxyuridine	0.68
383			3-ureidopropionate	353.07
384			beta-alanine	1.49
385		Pyrimidine Metabolism, Cytidine containing	cytidine diphosphate	0.31
386			cytidine 5'-monophosphate (5'-CMP)	0.44
387			cytidine	0.45
388			cytosine	2.15
389			3-methylcytidine	0.61
390			5-methylcytidine	0.27
391			2'-deoxycytidine	0.60
392			2'-O-methylcytidine	0.38
393			5-methyl-2'-deoxycytidine	0.45
394		Pyrimidine Metabolism, Thymine containing	3-aminoisobutyrate	0.52
395		Purine and Pyrimidine Metabolism	methylphosphate	0.56
396		Dinucleotide	(3'-5')-adenylylcytidine	0.24
397			(3'-5')-adenylyluridine	0.18
398			(3'-5')-uridylyluridine	0.20
399			(3'-5')-adenylyladenosine*	0.22
400			(3'-5')-guanylylcytidine	0.27
401			(3'-5')-uridylylcytidine*	0.39
402	Cofactors and Vitamins	Nicotinate and Nicotinamide Metabolism	quinolinate	2.10
403			nicotinate	0.83
404			nicotinate ribonucleoside	2.24
405			nicotinamide	0.84
406			nicotinamide adenine dinucleotide (NAD+)	0.51
407			1-methylnicotinamide	3.02
408			trigonelline (N'-methylnicotinate)	0.04
409			N1-Methyl-2-pyridone-5-carboxamide	1.41
410		Riboflavin Metabolism	riboflavin (Vitamin B2)	0.70
411			flavin adenine dinucleotide (FAD)	0.55
412			flavin mononucleotide (FMN)	0.58
413		Pantothenate and CoA Metabolism	pantothenate	0.58
414		Ascorbate and Aldarate Metabolism	ascorbate (Vitamin C)	1.99
415			oxalate (ethanedioate)	0.72
416			gulonate*	0.43
417		Tocopherol Metabolism	alpha-tocopherol	2.56
418			gamma-tocopherol/beta-tocopherol	6.27

419		Tetrahydrobiopterin Metabolism	biopterin	0.70
420			dihydrobiopterin	0.59
421		Pterin Metabolism	pterin	0.55
422			xanthopterin	0.46
423		Hemoglobin and Porphyrin Metabolism	bilirubin (Z,Z)	3.39
424			biliverdin	1.57
425		Thiamine Metabolism	thiamin (Vitamin B1)	0.65
426			thiamin monophosphate	0.53
427		Vitamin A Metabolism	retinal	0.16
428		Vitamin B6 Metabolism	pyridoxamine phosphate	0.57
429			pyridoxal phosphate	0.34
430			pyridoxal	0.71
431	Xenobiotics	Benzoate Metabolism	hippurate	0.38
432			catechol sulfate	0.33
433			phenylpropionylglycine	0.03
434		Food Component/Plant	2,3-dihydroxyisovalerate	0.32
435			2,8-quinolinediol	0.46
436			beta-guanidinopropanoate	0.39
437			cinnamoylglycine	0.37
438			enterolactone	0.06
439			ergothioneine	0.01
440			kojibiose	0.67
441			stachydrine	0.13
442			tartarate	16.59
443			methyl glucopyranoside (alpha + beta)	0.46
444		Bacterial/Fungal	tartronate (hydroxymalonate)	0.57
445		Chemical	sulfate*	0.63
446			S-(3-hydroxypropyl)mercapturic acid (HPMA)	0.23
447			perfluorooctanesulfonate (PFOS)	0.06
448			3-hydroxypyridine sulfate	0.16

Green: indicates significant difference ($p \leq 0.05$) between the groups shown, metabolite ratio of < 1.00

Red: indicates significant difference ($p \leq 0.05$) between the groups shown; metabolite ratio of ≥ 1.00
