# Supplementary material for: Significant alteration of liver metabolites by AAV8.Urocortin 2 gene transfer in mice with insulin resistance
Source: PLoS One. 2019 Dec 2;14(12):e0224428. doi: 10.1371/journal.pone.0224428 (PMC6886859; doi:10.1371/journal.pone.0224428)
Supplement: S4 Table — (PDF) [file pone.0224428.s005.pdf]

Supplementary Table 4. Effects of diet and AAV8.Ucn2 on Chow-fed and HFD-fed mice liver metabolites.													
#	Super Pathway	Sub Pathway	Biochemical Name	Mean				*p*-value			dark blue, p≤0.05; light blue, 0.05<p<0.10		
				Chow-Saline	HFD-Saline	Chow-Ucn2	HFD-Ucn2	Ucn2 Effect	Diet Effect	Interaction	Ucn2 Effect	Diet Effect	Interaction
1		Glycine, Serine and Threonine Metabolism	glycine	1.5318	0.9684	1.9046	1.0546	0.0024	0.0000	0.1334			
2			N-acetylglycine	2.6386	1.0002	4.6852	1.1601	0.0201	0.0000	0.0832			
3			sarcosine	1.4724	1.4573	1.4732	1.1773	0.1568	0.7677	0.8439			
4			dimethylglycine	0.9766	1.2433	1.0934	1.1281	0.9572	0.3298	0.3495			
5			betaine	0.7625	1.2508	1.6710	1.3296	0.0023	0.2814	0.0056			
6			betaine aldehyde	1.7846	0.4476	4.2902	0.8153	0.0014	0.0000	0.4982			
7			serine	1.6891	1.0273	1.9505	1.0715	0.0059	0.0000	0.1128			
8			N-acetylserine	1.5321	1.0792	2.5041	1.0167	0.0049	0.0000	0.0007			
9			threonine	1.6180	1.0125	2.0012	1.0079	0.0189	0.0000	0.0115			
10			N-acetylthreonine	1.5807	0.9403	3.7505	1.0949	0.0000	0.0000	0.0002			
11		Alanine and Aspartate Metabolism	alanine	1.3219	0.9628	1.3530	1.0319	0.3941	0.0000	0.7443			
12			N-acetylalanine	1.5055	1.0763	1.8046	1.0490	0.2824	0.0000	0.1858			
13			aspartate	1.6503	1.0055	2.4269	1.0707	0.0014	0.0000	0.0170			
14			N-acetylaspartate (NAA)	2.4370	1.0511	4.1610	1.5214	0.0131	0.0000	0.3403			
15			asparagine	1.2952	1.0462	1.2861	1.0886	0.7967	0.0005	0.5572			
16			N-acetylasparagine	1.5898	0.8894	1.7790	1.0406	0.2956	0.0001	0.9791			
17		Glutamate Metabolism	glutamate	1.7558	0.8082	2.2432	1.0194	0.0192	0.0000	0.8734			
18			glutamine	0.6328	1.1664	0.4767	1.3176	0.3400	0.0000	0.0323			
19			alpha-ketoglutaramate*	1.3269	1.0732	1.3111	1.1075	0.7571	0.0882	0.8266			
20			N-acetylglutamate	1.1436	0.8318	1.5629	1.1861	0.0800	0.0484	0.7495			
21			N-acetylglutamine	1.3369	0.9418	1.0327	1.0017	0.4300	0.0398	0.0684			
22			gamma-carboxyglutamate	1.2700	0.8787	1.6483	1.0965	0.0013	0.0000	0.7821			
23			glutamate, gamma-methyl ester	1.5228	0.8069	1.5412	1.2065	0.2598	0.0082	0.0682			
24			pyroglutamine*	2.1473	1.0231	2.0546	1.1214	0.8516	0.0000	0.3486			
25			carboxyethyl-GABA	1.3445	0.6562	0.8282	0.9202	0.4052	0.1849	0.0695			
26			N-methyl-GABA	2.2677	1.2967	2.3783	1.1013	0.9342	0.0005	0.7189			
27			S-1-pyrroline-5-carboxylate	1.2255	0.6448	1.3112	1.5694	0.0444	0.3541	0.1466			
28		Histidine Metabolism	histidine	1.3640	1.0165	2.8428	1.0668	0.0000	0.0000	0.0000			
29			1-methylhistidine	1.4754	0.9750	1.8169	1.3419	0.0657	0.0130	0.5618			
30			3-methylhistidine	1.7911	0.9253	2.6616	1.2044	0.1223	0.0005	0.5591			
31			N-acetylhistidine	1.6312	0.7788	1.7904	1.1363	0.0342	0.0000	0.2169			
32			N-acetyl-3-methylhistidine*	1.3156	0.3887	1.7343	0.7314	0.2302	0.0000	0.8166			
33			N-acetyl-1-methylhistidine*	2.6002	0.6261	3.5877	1.2768	0.0100	0.0000	0.2504			
34			imidazole propionate	1.9801	0.8362	0.5422	0.7514	0.1943	0.2309	0.2449			
35			formiminoglutamate	4.3343	1.0623	23.7655	0.8382	0.0208	0.0000	0.0020			
36			imidazole lactate	0.8409	1.0079	1.0175	0.7814	0.9028	0.9057	0.0845			
37			anserine	1.8331	0.9857	3.2903	1.1995	0.0082	0.0000	0.1253			
38			1-methylhistamine	1.8350	0.4513	1.7749	1.1534	0.1571	0.0069	0.2405			
39			1-methyl-4-imidazoleacetate	1.4657	1.0679	1.6245	1.3302	0.1693	0.0204	0.7869			

40			1-ribosyl-imidazoleacetate*	4.1760	0.4391	4.9876	0.7822	0.0170	0.0000	0.2890			
41			4-imidazoleacetate	2.4409	0.8226	2.2968	0.9389	0.6416	0.0000	0.4149			
42		Lysine Metabolism	lysine	1.3987	1.0010	1.6596	1.0278	0.0667	0.0000	0.1215			
43			N2-acetyllysine	1.6747	0.5892	3.4492	0.8102	0.0296	0.0000	0.0995			
44			N6-acetyllysine	2.0371	0.9660	4.1365	1.1449	0.0044	0.0000	0.0161			
45			N6,N6,N6-trimethyllysine	1.4315	1.0021	2.1485	1.0281	0.0108	0.0000	0.0167			
46			5-(galactosylhydroxy)-L-lysine	0.8101	1.2857	0.9483	1.2342	0.2632	0.0000	0.0740			
47			saccharopine	0.5219	0.9492	0.8072	1.1539	0.0439	0.0049	0.4184			
48			2-aminoadipate	0.8280	0.9398	1.7393	2.1268	0.0892	0.9297	0.4881			
49			glutarylcarnitine (C5-DC)	0.9754	0.7952	1.3238	1.3595	0.0020	0.3046	0.3374			
50			pipecolate	1.5814	0.9874	1.9640	1.1153	0.1041	0.0000	0.5262			
51			5-aminovalerate	1.1409	0.5893	0.7300	0.7901	0.7324	0.2085	0.0162			
52			N,N,N-trimethyl-5-aminovalerate	2.3688	1.4120	10.1552	0.9930	0.1902	0.0000	0.0109			
53		Phenylalanine Metabolism	phenylalanine	1.5064	1.0180	1.7716	1.0433	0.0022	0.0000	0.0191			
54			phenylpyruvate	2.0317	1.4014	2.3500	0.9246	0.3609	0.0003	0.0897			
55			phenyllactate (PLA)	0.8073	1.0208	0.7772	1.0667	0.6247	0.0226	0.9884			
56		Tyrosine Metabolism	tyrosine	1.5162	1.0455	1.6051	1.0481	0.3752	0.0000	0.4685			
57			4-hydroxyphenylpyruvate	1.0165	1.2760	1.4604	1.2529	0.2878	0.9313	0.2916			
58			3-(4-hydroxyphenyl)lactate	1.1672	1.1977	1.3315	1.0880	0.9191	0.5212	0.2386			
59			phenol sulfate	3.4447	0.9242	3.0034	0.6678	0.4442	0.0000	0.7756			
60			O-methyltyrosine	2.2632	0.5212	2.6077	0.9118	0.0881	0.0000	0.1676			
61			p-cresol glucuronide*	0.7030	0.6277	1.5785	1.9000	0.0009	0.4309	0.6662			
62			N-formylphenylalanine	1.7250	0.7967	2.7904	0.9189	0.1230	0.0000	0.2858			
63		Tryptophan Metabolism	tryptophan	1.3871	1.0133	1.6060	1.0392	0.0206	0.0000	0.0973			
64			C-glycosyltryptophan	1.5033	0.8968	2.2414	1.2377	0.0004	0.0000	0.5822			
65			kynurenine	1.9366	0.8673	7.2380	1.1672	0.0001	0.0000	0.0044			
66			kynurenate	1.5692	0.6129	7.4399	0.9285	0.0093	0.0001	0.1206			
67			xanthurenate	1.6204	0.6317	1.3425	1.1042	0.4491	0.0252	0.4372			
68			picolinate	0.7964	0.9140	1.3324	1.0952	0.0365	0.8131	0.3121			
69			serotonin	1.3477	1.2336	1.0723	1.2121	0.8130	0.4607	0.8050			
70			5-hydroxyindoleacetate	2.0092	0.6175	2.1173	0.8517	0.2936	0.0002	0.6410			
71			indolelactate	3.7005	0.9742	5.4165	0.9807	0.1149	0.0000	0.1268			
72			indole-3-carboxylate	1.6563	0.9290	2.0118	0.9747	0.3048	0.0000	0.6111			
73			indoleacetylglycine	2.6018	0.2438	3.0899	0.4282	0.0939	0.0000	0.8509			
74			3-indoxyl sulfate	2.2918	0.7935	2.3918	1.2053	0.0819	0.0000	0.2340			
75	Amino Acid		leucine	1.4775	1.0288	1.7659	1.0299	0.0222	0.0000	0.0165			
76			N-acetylleucine	1.2833	1.0996	1.3767	1.3530	0.2974	0.6612	0.9386			
77			4-methyl-2-oxopentanoate	0.3987	1.6391	0.3549	0.8470	0.0328	0.0000	0.0693			
78			isovalerylglycine	3.4128	0.8964	5.6708	1.2054	0.0890	0.0000	0.2330			

79	Leucine, Isoleucine and Valine Metabolism	3-methylcrotonylglycine	1.4682	0.2576	4.0875	0.6058	0.0039	0.0000	0.7949			
80		beta-hydroxyisovalerate	1.0320	1.1279	1.8599	1.0530	0.0583	0.1151	0.0158			
81		isoleucine	1.3664	1.0416	1.8392	1.0274	0.0009	0.0000	0.0004			
82		N-acetylisoleucine	1.1254	0.9785	1.5129	1.0700	0.0285	0.0077	0.2512			
83		2-hydroxy-3-methylvalerate	0.9890	0.8155	1.0538	1.0741	0.8414	0.7771	0.4326			
84		2-methylbutyrylcarnitine (C5)	0.9726	0.7320	2.1621	0.8373	0.1093	0.0206	0.2051			
85		2-methylbutyrylglycine	1.5394	1.0372	3.5843	1.2489	0.0064	0.0001	0.0303			
86		tiglylcarnitine (C5:1-DC)	1.2959	0.9481	2.9657	0.9836	0.0426	0.0021	0.0984			
87		3-hydroxy-2-ethylpropionate	0.7436	0.8777	0.5930	0.6585	0.1380	0.6037	0.9436			
88		ethylmalonate	1.4585	1.4563	0.8918	1.0799	0.0091	0.5742	0.5787			
89		methylsuccinate	1.6823	1.1991	1.8600	1.5178	0.1688	0.0360	0.7112			
90		valine	1.4931	1.0175	1.9729	1.0276	0.0014	0.0000	0.0018			
91		N-acetylvaline	1.6569	0.9696	2.5977	1.0075	0.0052	0.0000	0.0244			
92		3-methyl-2-oxobutyrate	0.4345	2.6522	0.4185	1.4052	0.0504	0.0000	0.0425			
93		alpha-hydroxyisovalerate	0.8083	0.9872	0.8763	1.2736	0.0340	0.0009	0.2419			
94		isobutyrylcarnitine (C4)	2.9096	0.5151	3.2379	0.9041	0.0072	0.0000	0.0430			
95		3-hydroxyisobutyrate	1.4546	0.9372	1.6481	1.0785	0.6353	0.0108	0.8883			
96	Methionine, Cysteine, SAM and Taurine Metabolism	methionine	1.5555	1.0051	2.0435	1.0741	0.0004	0.0000	0.0178			
97		N-acetylmethionine	1.2926	1.0847	1.6188	1.1638	0.0384	0.0011	0.3321			
98		N-formylmethionine	2.4061	1.0527	3.0139	1.0220	0.2662	0.0000	0.1048			
99		S-methylmethionine	1.5727	0.2164	2.6606	0.0779	0.9458	0.0000	0.0812			
100		methionine sulfoxide	1.3072	1.0633	1.1916	0.9850	0.3084	0.0404	0.9014			
101		N-acetylmethionine sulfoxide	1.1357	1.4895	1.3114	1.1815	0.5999	0.9762	0.2981			
102		S-adenosylmethionine (SAM)	0.4125	1.4797	0.1130	1.2112	0.0526	0.0000	0.1399			
103		S-adenosylhomocysteine (SAH)	1.4427	0.4942	1.6648	0.9419	0.0002	0.0000	0.0117			
104		cystathionine	0.9318	1.0866	0.9206	1.2678	0.7178	0.0350	0.2430			
105		alpha-ketobutyrate	0.6921	1.3092	0.5830	0.9956	0.3141	0.0013	0.8208			
106		cysteine	1.0941	1.0491	0.6680	1.9518	0.6728	0.0155	0.1746			
107		S-methylcysteine	1.0060	1.3535	1.6388	1.2529	0.0611	0.9349	0.0133			
108		cystine	1.3694	1.7245	2.2233	1.0211	0.9986	0.1024	0.0007			
109		hypotaurine	1.5448	0.7695	3.9403	0.9219	0.0026	0.0000	0.0140			
110		taurine	0.9698	1.0192	0.5331	1.0292	0.0001	0.0000	0.0001			
111		N-acetyltaurine	1.2316	1.2193	1.1227	1.1631	0.6057	0.9413	0.8830			
112		taurocyamine	1.0503	1.1504	1.6715	1.2560	0.0324	0.3768	0.1199			
113		arginine	0.9531	1.3137	1.0629	1.1359	0.8413	0.0199	0.1429			
114		urea	1.4556	0.9684	2.3278	1.1863	0.0068	0.0000	0.1334			
115		ornithine	1.2841	1.0516	1.5298	0.9967	0.0461	0.0000	0.0006			
116		2-oxoarginine*	1.1885	0.8587	1.1269	1.2169	0.6428	0.1775	0.4710			
117		citrulline	1.4455	0.9278	2.0791	0.9767	0.0188	0.0000	0.0727			

118		Urea cycle; Arginine and Proline Metabolism	homocitrulline	1.9965	0.2370	1.7740	0.9266	0.0042	0.0000	0.0016			
119			proline	1.4484	1.0207	1.8256	1.0068	0.0381	0.0000	0.0165			
120			dimethylarginine (SDMA + ADMA)	1.8815	1.0110	2.5096	1.1009	0.0150	0.0000	0.1294			
121			N-acetylarginine	1.3373	0.5191	1.4689	0.7659	0.2496	0.0000	0.8105			
122			N-acetylcitrulline	3.4436	0.7528	8.6809	1.4636	0.1210	0.0000	0.3172			
123			N-delta-acetylornithine	0.8445	0.6081	3.8076	0.6667	0.0000	0.0000	0.0000			
124			N-alpha-acetylornithine	0.8182	1.0435	1.6231	0.4985	0.5864	0.0045	0.0012			
125			trans-4-hydroxyproline	1.9894	0.9878	1.6994	0.8492	0.1033	0.0000	0.8891			
126			N-monomethylarginine	1.6828	0.9356	2.0284	1.0916	0.0257	0.0000	0.7558			
127			argininate*	0.6326	0.9520	0.7570	1.4402	0.0937	0.0028	0.6045			
128		Creatine Metabolism	guanidinoacetate	1.8315	0.9767	2.1028	1.0686	0.2771	0.0000	0.8330			
129			creatine	1.4554	1.1101	1.2899	1.0861	0.6761	0.0559	0.9771			
130			creatinine	1.5498	1.0543	1.4784	1.4042	0.3207	0.1395	0.5248			
131		Polyamine Metabolism	putrescine	0.1603	2.3322	0.1454	1.0941	0.0023	0.0000	0.0092			
132			spermidine	0.9281	1.2183	0.6719	0.9932	0.0014	0.0002	0.4648			
133			(N(1) + N(8))-acetylspermidine	0.9471	1.5502	0.6413	1.0994	0.0010	0.0000	0.6775			
134			5-methylthioadenosine (MTA)	0.6107	1.3973	0.5630	1.4457	0.8447	0.0000	0.8918			
135		Guanidino and Acetamido Metabolism	4-guanidinobutanoate	6.0268	0.8735	9.8416	0.7765	0.1907	0.0000	0.0222			
136			guanidinosuccinate	0.7726	0.7104	0.5793	1.7443	0.2593	0.0044	0.0028			
137		Glutathione Metabolism	glutathione, reduced (GSH)	1.8963	0.7945	0.3457	4.0906	0.7039	0.0163	0.0630			
138			glutathione, oxidized (GSSG)	1.1933	0.8108	1.3044	0.9549	0.0276	0.0000	0.4416			
139			cysteine-glutathione disulfide	1.1908	1.2647	1.4464	1.0313	0.8097	0.1314	0.0218			
140			S-methylglutathione	0.6252	1.1419	0.7606	1.1994	0.3458	0.0000	0.4825			
141			5-oxoproline	1.0353	0.9864	1.3281	1.0322	0.0052	0.0036	0.0419			
142			2-hydroxybutyrate/2-hydroxyisobutyrate	0.9883	1.0638	2.0779	1.1732	0.0008	0.0320	0.0040			
143			ophthalmate	3.0035	1.7132	2.5584	1.1365	0.7463	0.0103	0.6643			
144			4-hydroxy-nonenal-glutathione	1.5689	0.5971	0.8050	0.9910	0.9271	0.1669	0.0085			
145		Gamma-glutamyl Amino Acid	gamma-glutamylglutamate	2.2170	0.8185	2.5664	1.1708	0.0011	0.0000	0.1154			
146			gamma-glutamylglutamine	1.2249	1.0900	1.0595	1.6562	0.5691	0.7516	0.6141			
147			gamma-glutamylglycine	6.7344	0.9310	5.9257	1.0643	0.5962	0.0000	0.4399			
148			gamma-glutamylisoleucine*	1.5554	0.8858	2.6245	1.1512	0.0001	0.0000	0.1465			
149			gamma-glutamylleucine	1.6293	0.8365	2.5075	1.1978	0.0000	0.0000	0.4873			
150			gamma-glutamyl-epsilon-lysine	0.6995	1.8954	1.1837	1.4396	0.2334	0.0000	0.0001			
151			gamma-glutamylphenylalanine	1.9655	0.8958	3.1329	1.1415	0.0048	0.0000	0.2737			
152			gamma-glutamylthreonine	3.2616	0.5582	2.3270	1.0805	0.2282	0.0000	0.0581			
153			gamma-glutamylvaline	2.0517	1.0078	2.3754	1.2013	0.2348	0.0001	0.9360			

154			glycylisoleucine	1.9709	1.0768	2.9059	1.6946	0.0115	0.0003	0.7313			
155	Peptide		glycylleucine	2.1811	1.1910	2.4550	1.4246	0.4119	0.0003	0.6784			
156			glycylvaline	2.2555	1.2750	3.5111	1.5268	0.0766	0.0000	0.2888			
157			isoleucylglycine	1.5793	0.8641	1.5966	1.5390	0.1826	0.1006	0.0701			
158			leucylglycine	1.8201	0.9668	2.0693	1.9285	0.0565	0.0896	0.1372			
159			phenylalanylalanine	1.6429	1.0804	1.4514	1.7984	0.3266	0.6379	0.0672			
160		Dipeptide	phenylalanylglycine	1.3278	1.6787	1.1013	2.0806	0.8466	0.0074	0.1874			
161			prolylglycine	2.5350	1.0300	3.0555	1.2608	0.1210	0.0000	0.9078			
162			threonylphenylalanine	1.2415	0.8230	1.2053	1.4352	0.3054	0.5720	0.0859			
163			tyrosylglycine	1.5415	1.7654	1.7990	1.7662	0.7691	0.7304	0.5835			
164			valylglutamine	0.9638	0.5610	1.0409	1.1587	0.2606	0.8628	0.2090			
165			valylglycine	1.2835	0.9394	1.3816	1.7675	0.1317	0.9397	0.1069			
166			valylleucine	1.9196	1.0672	1.6956	2.7115	0.1755	0.8435	0.1076			
167		Acetylated Peptides	phenylacetylglycine	2.2261	0.7030	2.3527	1.3609	0.0475	0.0000	0.1356			
168			1,5-anhydroglucitol (1,5-AG)	2.6180	0.7717	3.9918	0.9212	0.0005	0.0000	0.1202			
169			glucose	1.2548	0.8923	0.4819	1.0034	0.0003	0.0276	0.0000			
170			glucose 6-phosphate	1.1446	0.6448	1.2854	0.9129	0.0237	0.0001	0.2272			
171			2,3-diphosphoglycerate	1.5081	1.0661	1.0716	1.5335	0.7016	0.8955	0.4469			
172		Glycolysis, Gluconeogenesis, and Pyruvate Metabolism	dihydroxyacetone phosphate (DHAP)	2.3008	1.4659	1.6185	1.1382	0.0623	0.8729	0.3318			
173			3-phosphoglycerate	0.6240	0.8756	0.6463	1.0751	0.2346	0.0273	0.6593			
174			phosphoenolpyruvate (PEP)	0.7753	0.7372	0.9038	0.9741	0.1462	0.4174	0.5691			
175			pyruvate	0.7814	1.2542	0.8293	1.1600	0.9548	0.0247	0.4860			
176			lactate	1.2071	1.1515	0.7062	1.0778	0.0005	0.0132	0.0040			
177			glycerate	1.5192	1.0888	0.8049	1.0235	0.0880	0.6149	0.1800			
178		Pentose Phosphate Pathway	6-phosphogluconate	1.3933	0.6206	1.1292	1.0933	0.1841	0.5620	0.6837			
179			sedoheptulose-7-phosphate	1.1036	0.6241	0.8913	0.9796	0.1867	0.0588	0.0051			
180			ribose	1.3018	0.8963	0.9891	1.1560	0.7853	0.1526	0.0033			
181			ribitol	0.9097	0.4767	1.0918	0.9564	0.0002	0.0004	0.0210			
182			ribonate	0.8242	0.9597	0.5925	0.9698	0.1814	0.0025	0.1757			
183		Pentose Metabolism	ribulose/xylulose	1.6282	1.2420	0.7632	1.0196	0.0003	0.6453	0.0172			
184			arabitol/xylitol	1.1568	1.1434	0.9730	1.2222	0.4228	0.2701	0.1638			
185			arabonate/xylonate	1.2428	0.9891	1.0879	0.7497	0.0365	0.0071	0.6219			
186			sedoheptulose	1.3845	0.5117	0.4477	0.8659	0.0153	0.2648	0.0000			
187			ribulonate/xylulonate/lyxonate*	1.5878	0.8903	0.8504	0.7786	0.0078	0.0352	0.0659			
188			maltopentaose	0.5499	0.4247	0.0484	1.0195	0.3691	0.1377	0.0469			
189		Glycogen Metabolism	maltotetraose	0.9153	0.5984	0.0109	1.1349	0.0010	0.0018	0.0012			
190			maltotriose	0.9439	0.6357	0.0597	0.9052	0.0004	0.0028	0.0003			
191			maltose	1.4385	0.9168	0.1446	0.9500	0.0000	0.0003	0.0000			
192	Carbohydrate		fructose	1.9014	0.6325	0.2336	0.8152	0.0000	0.5586	0.0000			
193			mannitol/sorbitol	0.8154	0.4530	0.2598	1.1006	0.1019	0.0011	0.0000			
194		Fructose, Mannose and Galactose Metabolism	mannose	1.4250	0.7335	0.5336	0.8292	0.0001	0.2830	0.0000			
195			galactose 1-phosphate	1.2740	0.7420	1.1931	0.9172	0.9469	0.0186	0.2052			

196		m	2-ketogulonate	1.4108	0.7112	1.3265	0.8656	0.6969	0.0001	0.3746
197			galactonate	1.8686	0.5823	1.8678	1.0163	0.0863	0.0000	0.1477
198		Nucleotide Sugar	UDP-glucose/UDP-galactose	0.9444	1.2180	3.0321	0.9346	0.3664	0.0494	0.0080
199			UDP-N-acetylglucosamine/galactosamine	1.5002	0.9352	0.8640	1.1020	0.2461	0.6438	0.0419
200		Aminosugar Metabolism	glucuronate	1.5426	1.0423	1.0317	0.7141	0.0347	0.0037	0.7135
201			N-acetylglucosamine 6-phosphate	0.8445	0.8063	0.5209	1.0688	0.5031	0.0155	0.0179
202			N-acetyl-glucosamine 1-phosphate	1.8798	0.7528	0.9525	1.0566	0.8614	0.1637	0.0359
203			N-acetylneuraminate	1.1523	0.8447	1.6713	0.9618	0.0368	0.0007	0.3208
204			N-acetylglucosaminylasparagine	0.5234	0.5991	0.6601	1.1736	0.0170	0.0339	0.1152
205			erythronate*	1.0832	1.1379	0.9622	1.0549	0.1031	0.2363	0.7291
206			N-acetylglucosamine/N-acetylgalactosamine	1.6037	1.1199	2.9727	1.0553	0.0200	0.0000	0.0024
207			N-glycolylneuraminate	1.1465	0.9391	1.6711	1.0062	0.0932	0.0039	0.1880
208		Advanced Glycation End-product	N6-carboxymethyllysine	0.9251	0.1324	1.2886	0.1324	0.0114	0.0000	0.0114
209	Energy	TCA Cycle	citrate	1.5321	0.8329	1.7634	2.0193	0.1910	0.1489	0.4769
210			aconitate [cis or trans]	1.2230	0.9113	1.5287	1.0720	0.1881	0.3439	0.4348
211			alpha-ketoglutarate	0.9396	0.9617	1.1628	1.1246	0.1998	0.9334	0.6152
212			succinylcarnitine (C4-DC)	0.6310	0.9872	0.4269	1.3486	0.5167	0.0001	0.0331
213			succinate	0.7006	1.1074	0.7257	1.1334	0.9758	0.0004	0.9570
214			fumarate	0.9606	0.8992	1.2583	1.0395	0.0018	0.0388	0.2636
215			malate	0.9178	0.9694	1.0983	1.0536	0.0007	0.8682	0.1523
216			itaconate	1.1515	1.9964	1.9599	1.3775	0.4045	0.8477	0.0406
217			tricarballylate	3.8100	3.2679	3.3577	1.3984	0.7043	0.0558	0.6531
218			2-methylcitrate/homocitrate	1.6849	1.1182	2.0930	1.0751	0.3539	0.0000	0.1054
219		Oxidative Phosphorylation	acetylphosphate	1.0410	0.7888	1.2628	0.8426	0.3573	0.0623	0.5006
220			phosphate	1.1631	0.9360	1.2203	1.0403	0.0041	0.0000	0.2474
221		Fatty Acid Synthesis	malonylcarnitine	1.6545	0.7693	2.3661	0.9395	0.0129	0.0000	0.3802
222			malonate	0.9792	1.0297	1.0555	0.9545	0.9666	0.7102	0.3463
223		Medium Chain Fatty Acid	5-dodecenoate (12:1n7)	1.8617	1.0700	3.0506	0.9741	0.0332	0.0000	0.0041
224		Long Chain Saturated Fatty Acid	myristate (14:0)	0.9708	2.5427	0.4306	0.8191	0.0000	0.0006	0.3688
225			pentadecanoate (15:0)	1.1092	1.1660	0.7028	0.7174	0.0006	0.8390	0.9353
226			palmitate (16:0)	1.0364	1.3609	0.6448	0.7835	0.0008	0.1613	0.8230
227			margarate (17:0)	1.0522	1.5026	0.5635	0.7110	0.0020	0.3193	0.9090
228			stearate (18:0)	1.1052	1.2709	0.6637	0.7070	0.0016	0.6963	0.8488

229		nonadecanoate (19:0)	1.8400	1.1139	1.0588	0.6435	0.0063	0.0137	0.8800
230		arachidate (20:0)	1.5154	1.2916	0.5477	0.7609	0.0007	0.9481	0.2278
231		behenate (22:0)*	2.0159	1.2060	0.6981	0.6276	0.0150	0.1371	0.3364
232	Long Chain Monounsaturated Fatty Acid	myristoleate (14:1n5)	0.9616	1.6464	1.0181	0.8212	0.0018	0.0720	0.0003
233		palmitoleate (16:1n7)	1.1024	2.2153	0.4611	0.7775	0.0000	0.0120	0.7937
234		10-heptadecenoate (17:1n7)	1.0088	2.3251	0.4498	0.8147	0.0002	0.0144	0.7537
235		oleate/vaccenate (18:1)	0.8845	1.4981	0.4873	0.7815	0.0006	0.0135	0.9726
236		10-nonadecenoate (19:1n9)	1.1356	2.0926	0.5859	0.7376	0.0009	0.2018	0.4840
237		eicosenoate (20:1)	0.9106	1.9780	0.3621	0.7731	0.0012	0.0379	0.9066
238		erucate (22:1n9)	1.8707	1.4470	0.6825	0.6599	0.0002	0.5387	0.5060
239	Long Chain Polyunsaturated Fatty Acid (n3 and n6)	stearidonate (18:4n3)	1.6054	1.2817	0.9944	0.7229	0.0020	0.0933	0.9546
240		eicosapentaenoate (EPA; 20:5n3)	2.2970	1.4168	1.0728	0.6303	0.0004	0.0557	0.7658
241		heneicosapentaenoate (21:5n3)	1.8197	1.5106	0.7340	0.6802	0.0002	0.8171	0.6840
242		docosapentaenoate (n3 DPA; 22:5n3)	1.4884	1.6312	0.8117	0.6769	0.0023	0.9257	0.7747
243		docosahexaenoate (DHA; 22:6n3)	1.0235	1.5482	0.6903	0.7409	0.0033	0.3213	0.4511
244		docosatrienoate (22:3n3)	1.1241	1.3625	0.5892	0.7211	0.0055	0.6090	0.9230
245		nisinate (24:6n3)	1.1827	1.5570	0.7332	0.7395	0.0033	0.4704	0.6844
246		hexadecadienoate (16:2n6)	1.4755	1.2730	1.1456	0.8278	0.0406	0.1126	0.7135
247		linoleate (18:2n6)	1.3241	1.2530	0.8611	0.7423	0.0022	0.5005	0.7982
248		linolenate [alpha or gamma; (18:3n3 or 6)]	1.8245	1.4398	1.0222	0.6293	0.0006	0.0993	0.7288
249		dihomo-linoleate (20:2n6)	0.9947	1.9721	0.5180	0.6904	0.0011	0.1422	0.5362
250		dihomo-linolenate (20:3n3 or n6)	1.0742	1.3678	0.6295	0.7318	0.0034	0.4198	0.9293
251		arachidonate (20:4n6)	1.0912	1.2273	0.7122	0.6962	0.0020	0.8281	0.6760
252		docosatrienoate (22:3n6)*	0.5503	2.5548	0.2745	0.8315	0.0010	0.0004	0.3914
253		adrenate (22:4n6)	1.0076	1.8056	0.6435	0.7476	0.0020	0.1535	0.4442
254		docosapentaenoate (n6 DPA; 22:5n6)	0.8528	1.6083	0.7234	0.6696	0.0039	0.1596	0.1075
255		docosadienoate (22:2n6)	1.2202	1.7112	0.5896	0.6589	0.0015	0.6220	0.8362
256		mead acid (20:3n9)	0.6463	1.9307	0.4534	0.6792	0.0127	0.0060	0.2089
257	Fatty Acid, Branched	(14 or 15)-methylpalmitate (a17:0 or i17:0)	1.2776	1.5724	0.8166	0.6737	0.0005	0.7765	0.5105
258		(16 or 17)-methylstearate (a19:0 or i19:0)	1.7416	1.4887	0.9648	0.6640	0.0035	0.1707	0.7657
259		glutarate (C5-DC)	0.9639	1.1729	1.1246	1.2019	0.9240	0.5260	0.5905
260		3-methylglutarate/2-methylglutarate	1.4155	0.8585	1.7185	1.3474	0.0109	0.0043	0.2122
261		2-hydroxyglutarate	0.8515	1.1488	0.7079	1.0685	0.0612	0.0000	0.4415
262		adipate (C6-DC)	1.4321	1.1107	2.1712	1.1123	0.0432	0.0001	0.0525
263		2-hydroxyadipate	1.9506	1.1138	2.8041	1.8023	0.2535	0.0044	0.8814
264		3-hydroxyadipate*	2.3858	1.0904	3.3476	1.2978	0.1130	0.0000	0.5578

265	Fatty Acid, Dicarboxylate	3-methyladipate	0.5596	0.2373	1.0388	0.3565	0.0276	0.0000	0.2226			
266		pimelate (C7-DC)	1.3555	0.9371	1.6198	0.7080	0.6223	0.0001	0.1035			
267		suberate (C8-DC)	1.1107	0.9830	0.8422	0.8063	0.0581	0.5398	0.6455			
268		azelate (C9-DC)	1.4997	0.9921	1.5578	0.9492	0.9732	0.0015	0.9102			
269		sebacate (C10-DC)	1.1736	0.9452	1.6339	0.8445	0.2924	0.0009	0.0647			
270		dodecanedioate (C12-DC)	1.2486	0.8609	1.5798	1.1903	0.0536	0.0538	0.7423			
271		dodecadienoate (12:2)*	1.2240	0.8648	0.8404	0.8957	0.2344	0.3260	0.0889			
272		tetradecanedioate (C14-DC)	0.8962	1.0718	1.4411	1.0854	0.0074	0.5139	0.0121			
273		hexadecanedioate (C16-DC)	1.1552	0.9881	2.3194	1.0739	0.0043	0.0013	0.0322			
274		hexadecenedioate (C16:1-DC)*	0.6509	1.2243	0.9315	1.1173	0.1630	0.0001	0.0158			
275		octadecanedioate (C18-DC)	2.4954	0.9365	2.0869	0.9118	0.4025	0.0005	0.4879			
276	Fatty Acid, Amino	2-aminooctanoate	1.0924	0.5426	1.1547	0.3905	0.7407	0.0000	0.2291			
277	Fatty Acid Metabolism (also BCAA Metabolism)	butyrylglycine	1.9058	0.8874	2.6307	0.9894	0.1818	0.0000	0.3469			
278		propionylcarnitine (C3)	0.8591	0.4597	1.4518	0.7696	0.0229	0.0014	0.7942			
279		propionylglycine	1.4222	0.3271	6.5451	0.8732	0.0033	0.0000	0.2286			
280		methylmalonate (MMA)	0.9037	1.2621	0.7485	1.1973	0.2104	0.0006	0.5651			
281	Fatty Acid Metabolism (Acyl Glycine)	isocaproylglycine	3.8139	0.7808	4.3920	1.2185	0.1718	0.0000	0.7593			
282		valerylglycine	1.6318	1.0654	4.2588	1.5946	0.0023	0.0012	0.0391			
283		hexanoylglycine	4.3898	0.7284	5.0770	1.0001	0.1771	0.0000	0.8553			
284		3,4-methylene heptanoylglycine	1.8718	0.7000	2.0332	0.9891	0.0514	0.0000	0.5168			
285		N-octanoylglycine	2.4055	0.0971	2.8940	0.1222	0.1409	0.0000	0.8452			
286		N-palmitoylglycine	2.3174	0.7630	1.6437	0.4056	0.0350	0.0006	0.6846			
287		N-linoleoylglycine	1.6523	0.4181	1.8153	0.4181	0.4591	0.0000	0.4591			
288	Fatty Acid Metabolism (Acyl Carnitine, Short Chain)	acetylcarnitine (C2)	0.9781	1.1952	1.7772	1.1012	0.2222	0.4410	0.0568			
289	Fatty Acid Metabolism (Acyl Carnitine, Long Chain Saturated)	palmitoylcarnitine (C16)	0.5330	1.2426	1.0967	1.0291	0.0907	0.0078	0.0027			
290		stearoylcarnitine (C18)	0.8172	1.1156	1.4524	0.9391	0.2029	0.8779	0.0208			
291		arachidoylcarnitine (C20)*	0.7764	1.0810	0.9719	0.9281	0.7444	0.3653	0.3746			
292		behenoylcarnitine (C22)*	1.2860	0.8115	0.7878	0.6830	0.2962	0.3747	0.8507			
293		lignoceroylcarnitine (C24)*	1.1811	0.9808	1.4580	0.8458	0.7604	0.0310	0.3255			
294	Fatty Acid Metabolism (Acyl Carnitine, Monounsaturated)	oleoylcarnitine (C18:1)	0.4863	1.3441	0.7632	1.1178	0.2265	0.0000	0.0033			
295		eicosenoylcarnitine (C20:1)*	0.4752	1.3414	0.7799	0.8700	0.9025	0.0751	0.0903			
296		erucoylcarnitine (C22:1)*	1.1183	2.0630	0.6852	1.2598	0.0066	0.0008	0.9964			
297	Fatty Acid Metabolism (Acyl Carnitine, Polyunsat	linoleoylcarnitine (C18:2)*	1.0770	0.6620	1.9307	1.1260	0.0000	0.0000	0.8593			
298		arachidonoylcarnitine (C20:4)	0.9944	0.8749	1.6740	1.0636	0.0044	0.0246	0.2137			
299	Fatty Acid Metabolism (Acyl	adipoylcarnitine (C6-DC)	1.6841	0.9907	1.8306	1.0943	0.5031	0.0169	0.8226			

300	m (Acyl Carnitine, Dicarboxylate)	pimeloylcarnitine/3-methyladipoylcarnitine (C7-DC)	1.9871	0.5278	2.4460	1.2844	0.0278	0.0001	0.2787			
301	Fatty Acid Metabolism (Acyl Carnitine, Hydroxy)	(R)-3-hydroxybutyrylcarnitine	1.0824	1.4275	1.0148	1.4600	0.7011	0.0175	0.5541			
302		(S)-3-hydroxybutyrylcarnitine	0.6368	1.1597	0.5752	1.2420	0.8292	0.0001	0.8599			
303	Carnitine Metabolism	deoxycarnitine	2.6074	0.8453	2.0890	1.0536	0.9567	0.0000	0.0732			
304		carnitine	0.9691	1.1092	1.0434	1.1263	0.4819	0.0628	0.7313			
305	Ketone Bodies	3-hydroxybutyrate (BHBA)	0.8745	1.1266	1.8580	1.2243	0.0019	0.3772	0.0069			
306	Fatty Acid Metabolism (Acyl Choline)	palmitoylcholine	0.8010	0.7888	3.6191	1.2326	0.0001	0.0221	0.0348			
307		oleoylcholine	0.4945	0.6724	1.3493	1.2438	0.0000	0.3093	0.2903			
308		palmitoloelycholine	0.8285	0.4725	2.3644	1.0118	0.0020	0.0060	0.6716			
309		linoleoylcholine*	1.5215	0.4594	6.6895	1.2852	0.0000	0.0000	0.5939			
310		stearoylcholine*	0.6272	0.6163	2.4592	1.2821	0.0010	0.4628	0.3417			
311		arachidonoylcholine	0.4098	0.3820	3.5247	0.6303	0.0007	0.0175	0.0288			
312	Fatty Acid, Monohydroxy	2-hydroxyheptanoate*	1.2209	1.0144	0.6392	0.9362	0.1756	0.5915	0.1162			
313		2-hydroxypalmitate	1.1155	0.9313	0.7052	0.6475	0.0032	0.4275	0.6668			
314		2-hydroxystearate	0.5215	0.4186	0.4364	0.2440	0.2939	0.1002	0.7931			
315		3-hydroxyhexanoate	1.0793	0.9069	0.8386	0.9871	0.5586	0.9201	0.2997			
316		3-hydroxyoctanoate	1.0012	0.8882	0.7505	0.7967	0.1683	0.8248	0.4288			
317		3-hydroxydecanoate	1.0005	1.0339	1.6275	0.9044	0.2747	0.0257	0.0141			
318		3-hydroxyoleate*	1.1668	1.5730	0.6795	0.7539	0.0002	0.1443	0.8594			
319		8-hydroxyoctanoate	1.0572	1.0102	1.1618	0.9060	0.9763	0.2728	0.6248			
320		16-hydroxypalmitate	1.2023	0.9027	1.9899	1.0125	0.0170	0.0010	0.1270			
321		13-HODE + 9-HODE	2.6483	0.8534	1.9454	1.0469	0.7100	0.0001	0.0861			
322	Fatty Acid, Dihydroxy	12,13-DiHOME	1.4780	0.9055	1.4064	1.2450	0.3983	0.0262	0.1944			
323		9,10-DiHOME	2.1757	0.9097	1.6359	1.1130	0.7408	0.0024	0.2403			
324		19,20-DiHDPA	0.5244	1.0216	0.6603	1.4068	0.0200	0.0000	0.8644			
325		5,6-DiHETrE	1.7736	0.9163	1.0966	0.8667	0.0613	0.0294	0.1172			
326		14,15-DiHETrE	1.2570	0.7813	1.4273	1.0017	0.0649	0.0001	0.4671			
327	Fatty Acid, Oxidized	4-hydroxynonenal	1.7542	0.4839	1.3257	0.4464	0.2332	0.0000	0.4778			
328	Docosanoid	14-HDoHE/17-HDoHE	1.0813	0.8112	0.9146	1.1124	0.7741	0.9880	0.1847			
329		4-HDoHE	2.9882	0.9717	1.0633	0.6886	0.0191	0.0143	0.1082			
330	Eicosanoid	prostaglandin F2alpha	1.0052	0.6661	1.2498	0.9868	0.1393	0.0818	0.3134			
331		5-HETE	2.5041	0.6211	0.7846	0.6005	0.0933	0.0729	0.0111			
332		12-HETE	0.9322	0.7405	0.6463	1.1755	0.7086	0.0650	0.0001			
333		15-HETE	2.2984	0.8100	1.5015	0.9032	0.4005	0.0003	0.0594			
334		12-HHTrE	1.4155	1.1196	1.9814	1.0016	0.7813	0.0206	0.3621			
335		oleoyl ethanolamide	1.3029	1.2635	1.4918	1.0038	0.5454	0.0181	0.0510			
336		palmitoyl ethanolamide	1.2499	1.2448	1.4585	1.1406	0.7757	0.1220	0.1636			
337		stearoyl ethanolamide	1.0814	0.9725	1.3666	1.1475	0.0152	0.0463	0.5002			
338		N-arachidonoyltaurine	1.6295	1.0299	0.6453	0.5820	0.0004	0.1148	0.3157			

339			N-oleoyltaurine	1.4050	2.0813	0.3422	0.5770	0.0000	0.1632	0.6449			
340		Endocannabinoid	N-stearoyltaurine	0.8438	1.3169	0.4038	0.6235	0.0009	0.2464	0.7876			
341			N-palmitoyltaurine	1.6462	1.8374	0.2699	0.3443	0.0000	0.8980	0.9562			
342			N-palmitoleoyltaurine*	1.8683	1.4668	0.3475	0.5209	0.0000	0.9027	0.1024			
343			N-linoleoyltaurine*	2.7722	0.9785	0.8952	0.5119	0.0004	0.0004	0.1683			
344			N-linolenoyltaurine*	3.4847	0.5273	1.1923	0.2779	0.0005	0.0000	0.1022			
345			linoleoyl ethanolamide	3.1012	1.0887	1.8783	0.4861	0.0049	0.0001	0.8243			
346		Inositol Metabolism	myo-inositol	1.5055	0.9388	1.9918	1.0508	0.0775	0.0000	0.5373			
347			inositol 1-phosphate (I1P)	0.9426	1.2894	2.0291	1.0048	0.2073	0.2054	0.0062			
348			choline	1.0268	0.9699	1.1694	1.0401	0.1087	0.1398	0.5707			
349			choline phosphate	0.6134	0.8537	0.7258	1.1186	0.0437	0.0031	0.6257			
350			cytidine 5'-diphosphocholine	1.3926	0.9283	1.0355	1.1459	0.9714	0.2645	0.0545			
351			glycerophosphorylcholine (GPC)	0.6039	1.6495	0.5664	1.0681	0.0075	0.0000	0.1334			
352		Phospholipid Metabolism	phosphoethanolamine	0.9693	1.2535	1.2427	1.1308	0.1965	0.1754	0.0051			
353			cytidine-5'-diphosphoethanolamine	2.5168	1.0978	1.3430	0.8347	0.0050	0.0004	0.1164			
354			glycerophosphoethanolamine	0.9123	1.4655	0.8614	1.0647	0.0191	0.0004	0.2922			
355			glycerophosphoserine*	0.9338	1.0685	1.1152	1.0332	0.4008	0.7330	0.1918			
356			glycerophosphoinositol*	1.1080	0.9568	1.5651	1.1062	0.0172	0.0076	0.2451			
357			trimethylamine N-oxide	0.9440	0.9763	0.7322	0.9511	0.5788	0.7191	0.8234			
358			1-myristoyl-2-palmitoyl-GPC (14:0/16:0)	1.2661	1.0204	1.3280	0.9493	0.7182	0.0000	0.1449			
359			1-myristoyl-2-arachidonoyl-GPC (14:0/20:4)*	1.3226	0.9203	1.3009	0.9007	0.5896	0.0000	0.7991			
360			1,2-dipalmitoyl-GPC (16:0/16:0)	1.2275	1.0870	1.5420	1.0206	0.0090	0.0000	0.0000			
361			1-palmitoyl-2-palmitoleoyl-GPC (16:0/16:1)*	1.5160	1.1798	1.3398	0.8439	0.0003	0.0000	0.0503			
362			1-palmitoyl-2-stearoyl-GPC (16:0/18:0)	0.8839	0.9912	1.1014	1.0262	0.0031	0.5970	0.0188			
363			1-palmitoyl-2-oleoyl-GPC (16:0/18:1)	1.0206	1.1008	1.1062	0.9645	0.4331	0.3392	0.0048			
364			1-palmitoyl-2-linoleoyl-GPC (16:0/18:2)	1.4637	0.9026	1.6431	1.0652	0.0007	0.0000	0.5780			
365			1-palmitoyl-2-gamma-linolenoyl-GPC (16:0/18:3n6)*	1.5906	0.9186	1.4815	1.1146	0.4219	0.0000	0.1236			
366			1-palmitoyl-2-dihomo-linolenoyl-GPC (16:0/20:3n3 or 6)*	0.9068	1.0443	0.8649	1.0433	0.5348	0.0007	0.5899			
367			1-palmitoyl-2-arachidonoyl-GPC (16:0/20:4n6)	1.0043	0.9408	1.0246	0.9998	0.1829	0.1125	0.5092			
368		Phosphatidylcholine (PC)	1-palmitoyl-2-docosahexaenoyl-GPC (16:0/22:6)	1.0885	0.8878	1.1830	1.0094	0.0026	0.0000	0.5117			

369			1-palmitoleoyl-2-linoleoyl-GPC (16:1/18:2)*	2.8122	0.9012	2.7364	1.0647	0.3711	0.0000	0.2171			
370			1-stearoyl-2-oleoyl-GPC (18:0/18:1)	0.7860	1.1135	0.7689	0.9828	0.0806	0.0000	0.2435			
371			1-stearoyl-2-linoleoyl-GPC (18:0/18:2)*	1.0870	0.9006	1.1093	1.0737	0.0601	0.0265	0.1256			
372			1-stearoyl-2-arachidonoyl-GPC (18:0/20:4)	0.8378	0.9403	0.7960	0.9812	0.8541	0.0001	0.1653			
373			1-stearoyl-2-docosahexaenoyl-GPC (18:0/22:6)	0.9648	0.8810	0.9573	1.0374	0.1105	0.8188	0.0880			
374			1-oleoyl-2-linoleoyl-GPC (18:1/18:2)*	1.4998	1.0350	1.4812	0.9819	0.5208	0.0000	0.9345			
375			1-oleoyl-2-docosahexaenoyl-GPC (18:1/22:6)*	1.2335	0.9169	1.3449	0.9698	0.1522	0.0000	0.6757			
376			1,2-dilinoleoyl-GPC (18:2/18:2)	2.2440	0.6362	2.8942	1.0590	0.0000	0.0000	0.1388			
377			1-linoleoyl-2-linolenoyl-GPC (18:2/18:3)*	9.2803	0.5195	14.5016	1.2613	0.0002	0.0000	0.2914			
378			1-linoleoyl-2-arachidonoyl-GPC (18:2/20:4n6)*	1.3855	0.7127	1.5480	1.0153	0.0011	0.0000	0.0792			
379			1,2-dipalmitoyl-GPE (16:0/16:0)*	1.4274	1.2508	1.8632	0.9259	0.8203	0.0000	0.0001			
380			1-palmitoyl-2-stearoyl-GPE (16:0/18:0)*	1.0715	1.0830	1.5363	0.8350	0.4269	0.0002	0.0001			
381			1-palmitoyl-2-oleoyl-GPE (16:0/18:1)	1.5804	1.1802	1.8793	0.9704	0.9326	0.0000	0.0213			
382			1-palmitoyl-2-linoleoyl-GPE (16:0/18:2)	4.2429	0.9111	4.8980	1.1330	0.1143	0.0000	0.9051			
383			1-palmitoyl-2-arachidonoyl-GPE (16:0/20:4)*	1.5201	0.9523	1.6157	0.9782	0.3360	0.0000	0.6353			
384			1-palmitoyl-2-docosahexaenoyl-GPE (16:0/22:6)*	1.3997	0.9749	1.5182	1.0287	0.2259	0.0000	0.7738			
385			1-stearoyl-2-oleoyl-GPE (18:0/18:1)	1.0002	1.1313	1.1861	0.9795	0.7389	0.4708	0.0038			
386		Phosphatidylethanolamine (PE)	1-stearoyl-2-linoleoyl-GPE (18:0/18:2)*	1.5113	0.7647	1.7149	1.0198	0.0032	0.0000	0.2837			
387			1-stearoyl-2-arachidonoyl-GPE (18:0/20:4)	1.1318	0.8569	1.1841	0.9540	0.0220	0.0000	0.3230			
388			1-stearoyl-2-docosahexaenoyl-GPE (18:0/22:6)*	1.3822	0.9474	1.5603	0.9788	0.1907	0.0000	0.3963			
389			1-oleoyl-2-linoleoyl-GPE (18:1/18:2)*	2.6458	0.8614	3.1193	1.0585	0.0169	0.0000	0.9190			
390			1-oleoyl-2-arachidonoyl-GPE (18:1/20:4)*	1.2946	0.9533	1.3630	0.9465	0.5667	0.0000	0.4183			
391			1-oleoyl-2-docosahexaenoyl-GPE (18:1/22:6)*	1.3852	1.0132	1.4479	0.9379	0.6951	0.0000	0.2079			

392			1,2-dilinoleoyl-GPE (18:2/18:2)*	7.3531	0.6381	8.6386	1.1690	0.0078	0.0000	0.1889			
393			1-linoleoyl-2-arachidonoyl-GPE (18:2/20:4)*	2.5844	0.7330	2.8698	1.0682	0.0087	0.0000	0.1733			
394		Phosphatidylserine (PS)	1-stearoyl-2-oleoyl-GPS (18:0/18:1)	1.1548	0.9662	1.4788	1.0493	0.0269	0.0006	0.1964			
395			1-stearoyl-2-arachidonoyl-GPS (18:0/20:4)	1.1748	0.8035	1.3214	1.0140	0.0072	0.0000	0.3299			
396		Phosphatidylglycerol (PG)	1-palmitoyl-2-oleoyl-GPG (16:0/18:1)	1.2083	0.9304	1.3427	0.8890	0.4393	0.0000	0.0651			
397			1-palmitoyl-2-linoleoyl-GPG (16:0/18:2)	1.2879	0.7329	1.5012	0.8893	0.1029	0.0000	0.9112			
398		Phosphatidylinositol (PI)	1-palmitoyl-2-linoleoyl-GPI (16:0/18:2)	4.2733	0.6335	6.4469	1.0259	0.0084	0.0000	0.8994			
399	Lipid		1-palmitoyl-2-arachidonoyl-GPI (16:0/20:4)*	1.5792	0.9162	1.7139	1.0044	0.1253	0.0000	0.9716			
400			1-stearoyl-2-linoleoyl-GPI (18:0/18:2)	2.8778	0.7012	4.6313	0.9856	0.0000	0.0000	0.3812			
401			1-oleoyl-2-linoleoyl-GPI (18:1/18:2)*	5.3443	0.6507	7.3831	1.0829	0.0002	0.0000	0.3707			
402			1-stearoyl-2-arachidonoyl-GPI (18:0/20:4)	1.1680	0.8328	1.2512	0.9687	0.0002	0.0000	0.1181			
403			1-oleoyl-2-arachidonoyl-GPI (18:1/20:4)*	1.4205	0.9821	1.8321	1.0031	0.0135	0.0000	0.0194			
404		Lysophospholipid	1-palmitoyl-GPC (16:0)	1.1016	0.9712	1.1540	0.9850	0.6443	0.0118	0.8277			
405			2-palmitoyl-GPC (16:0)*	1.5792	1.4242	0.9142	0.6644	0.0006	0.7641	0.8868			
406			1-palmitoleoyl-GPC (16:1)*	1.8240	1.0306	1.4684	0.9590	0.0799	0.0000	0.4375			
407			1-stearoyl-GPC (18:0)	0.7776	0.9554	0.7216	1.0077	0.7330	0.0000	0.1435			
408			1-oleoyl-GPC (18:1)	1.1819	1.1677	1.2107	0.9781	0.2168	0.1250	0.1416			
409			1-linoleoyl-GPC (18:2)	2.2340	1.3434	1.3363	0.7099	0.0026	0.0551	0.9023			
410			1-linolenoyl-GPC (18:3)*	3.1229	0.8691	3.3483	1.0941	0.1417	0.0000	0.4563			
411			1-arachidonoyl-GPC (20:4n6)*	1.3197	1.7257	0.6815	0.7234	0.0007	0.4585	0.8319			
412			1-palmitoyl-GPE (16:0)	1.7418	0.9647	1.7715	0.9429	0.8298	0.0000	0.8474			
413			1-stearoyl-GPE (18:0)	1.1597	0.8970	1.0995	0.9558	0.9999	0.0085	0.3331			
414			2-stearoyl-GPE (18:0)*	1.3390	1.1833	0.6590	0.5941	0.0010	0.7028	0.8271			
415			1-oleoyl-GPE (18:1)	1.4682	1.0209	1.4844	0.9521	0.5749	0.0002	0.7084			
416			1-linoleoyl-GPE (18:2)*	2.7854	0.8351	3.5290	1.0141	0.0799	0.0000	0.7593			

417			1-arachidonoyl-GPE (20:4n6)*	1.8419	1.3492	0.9646	0.6328	0.0005	0.1631	0.9243			
418			1-palmitoyl-GPS (16:0)*	2.1434	1.1501	1.3993	0.5852	0.0024	0.0023	0.5833			
419			1-stearoyl-GPS (18:0)*	1.1399	0.7566	1.4403	0.9494	0.0060	0.0000	0.9445			
420			1-oleoyl-GPS (18:1)	2.3459	1.8176	1.3040	0.5959	0.0004	0.0780	0.4238			
421			1-palmitoyl-GPG (16:0)*	1.6674	1.7432	0.7676	0.5770	0.0005	0.8149	0.7763			
422			1-stearoyl-GPG (18:0)	1.0528	1.5200	0.4539	0.6929	0.0009	0.1031	0.6579			
423			1-oleoyl-GPG (18:1)*	1.2089	5.0022	0.6699	0.7118	0.0001	0.0276	0.1359			
424			1-linoleoyl-GPG (18:2)*	1.5485	1.6513	0.7808	0.7067	0.0008	0.9271	0.9233			
425			1-palmitoyl-GPI (16:0)	2.3434	1.4649	1.3226	0.5542	0.0002	0.0059	0.5633			
426			1-stearoyl-GPI (18:0)	1.4234	1.2232	0.8313	0.5756	0.0006	0.2601	0.8316			
427			1-oleoyl-GPI (18:1)	2.3242	1.6806	1.5343	0.5126	0.0004	0.0238	0.3233			
428			1-linoleoyl-GPI (18:2)*	4.7028	0.8382	3.9892	0.5377	0.1426	0.0000	0.9461			
429			1-arachidonoyl-GPI (20:4)*	1.4192	1.4285	0.8044	0.6999	0.0019	0.6989	0.8622			
430		Plasmalogen	1-(1-enyl-palmitoyl)-2-oleoyl-GPE (P-16:0/18:1)*	1.5696	1.2820	1.8425	1.1262	0.7159	0.0009	0.1024			
431			1-(1-enyl-palmitoyl)-2-linoleoyl-GPE (P-16:0/18:2)*	2.9186	0.9337	3.4538	0.9459	0.5239	0.0000	0.6048			
432			1-(1-enyl-palmitoyl)-2-palmitoyl-GPC (P-16:0/16:0)*	2.0834	0.9863	2.6162	1.0872	0.0041	0.0000	0.1464			
433			1-(1-enyl-palmitoyl)-2-arachidonoyl-GPE (P-16:0/20:4)*	1.4838	1.1478	1.9019	1.0463	0.1105	0.0000	0.0022			
434			1-(1-enyl-palmitoyl)-2-oleoyl-GPC (P-16:0/18:1)*	1.6539	0.9035	1.6210	0.9713	0.9111	0.0061	0.6940			
435			1-(1-enyl-stearoyl)-2-oleoyl-GPE (P-18:0/18:1)	1.2779	0.8605	1.7603	0.8840	0.2110	0.0108	0.3225			
436			1-(1-enyl-stearoyl)-2-linoleoyl-GPE (P-18:0/18:2)*	2.0532	0.9639	2.3553	1.1301	0.2479	0.0001	0.7707			
437			1-(1-enyl-stearoyl)-2-arachidonoyl-GPE (P-18:0/20:4)*	0.8945	0.9662	1.1391	1.1176	0.0016	0.5294	0.3365			
438		Lysoplasmalogen	1-(1-enyl-palmitoyl)-GPE (P-16:0)*	1.5935	1.3983	1.9638	0.9429	0.2952	0.0183	0.0931			
439			1-(1-enyl-oleoyl)-GPE (P-18:1)*	1.1202	1.0481	1.2119	0.9085	0.7156	0.1111	0.2124			
440			1-(1-enyl-stearoyl)-GPE (P-18:0)*	1.1169	1.2453	1.3336	0.9764	0.5213	0.8889	0.2396			
441		Glycerolipid Metabolism	glycerol	0.7133	1.4041	0.6631	1.0889	0.0144	0.0000	0.1368			
442			glycerol 3-phosphate	3.0390	0.4768	2.9829	1.2218	0.0588	0.0000	0.1468			
443			glycerophosphoglycerol	0.5150	1.3630	0.4133	0.9212	0.0559	0.0000	0.6621			
444			1-myristoylglycerol (14:0)	1.0020	1.5511	0.2917	0.8013	0.0010	0.1293	0.6424			
445			1-palmitoylglycerol (16:0)	0.8418	1.9138	1.0047	1.1997	0.1409	0.0002	0.0036			
446			1-palmitoleoylglycerol (16:1)*	1.4752	2.1566	0.4143	1.1405	0.0013	0.1273	0.5643			
447			1-margaroylglycerol (17:0)	1.7380	1.9472	0.9958	0.8258	0.0174	0.8286	0.3188			
448			1-stearoylglycerol (18:0)	0.6034	1.8467	0.5819	1.1951	0.0063	0.0000	0.0192			
449			1-oleoylglycerol (18:1)	0.7778	1.2172	0.7905	1.2596	0.8938	0.0023	0.7328			
450			1-linoleoylglycerol (18:2)	1.4164	1.0418	2.4155	2.5681	0.1033	0.2689	0.9609			
451			1-linolenoylglycerol (18:3)	1.0920	1.0242	1.4382	1.6604	0.0635	0.9458	0.6796			

452		1-dihomo-linolenylglycerol (20:3)	1.3392	0.8878	2.1883	1.8709	0.0765	0.0853	0.8852
453	Monoacylglycerol	1-arachidonylglycerol (20:4)	1.6995	0.7075	2.5421	1.4417	0.0144	0.0002	0.4600
454		1-docosahexaenoylglycerol (22:6)	0.7747	0.9607	1.0044	1.8735	0.0190	0.0259	0.3513
455		2-myristoylglycerol (14:0)	0.7608	0.8469	0.2760	0.8423	0.2540	0.1874	0.2568
456		2-palmitoylglycerol (16:0)	1.4208	1.3477	0.8939	0.8048	0.0213	0.7855	0.7356
457		2-palmitoleoylglycerol (16:1)*	2.0305	1.5371	0.4653	1.1729	0.0026	0.8648	0.2801
458		2-stearoylglycerol (18:0)	1.0408	0.7592	1.3866	1.1222	0.3102	0.0971	0.8332
459		2-oleoylglycerol (18:1)	1.0823	0.9834	0.9785	1.5859	0.4946	0.3230	0.1114
460		2-linoleoylglycerol (18:2)	2.2275	0.6652	1.8235	1.7529	0.1407	0.0004	0.0121
461		2-arachidonoylglycerol (20:4)	3.0792	0.8670	1.6501	1.4063	0.2941	0.0024	0.2357
462		2-docosahexaenoylglycerol (22:6)*	0.9087	0.9884	0.4910	1.6271	0.4903	0.4057	0.3039
463		1-dihomo-linoleoylglycerol (20:2)	1.1450	1.0996	1.9951	1.3234	0.2118	0.1222	0.2112
464		diacylglycerol (12:0/18:1, 14:0/16:1, 16:0/14:1) [1]*	0.2473	1.9899	0.1775	0.6464	0.0107	0.0000	0.0363
465		diacylglycerol (12:0/18:1, 14:0/16:1, 16:0/14:1) [2]*	0.6914	1.5766	0.4403	0.9099	0.0000	0.0000	0.4082
466		diacylglycerol (14:0/18:1, 16:0/16:1) [1]*	0.5219	1.7904	0.3386	1.0459	0.0000	0.0000	0.4023
467		diacylglycerol (14:0/18:1, 16:0/16:1) [2]*	0.6218	1.3132	0.4175	0.8565	0.0000	0.0000	0.6365
468		diacylglycerol (16:1/18:2 [2], 16:0/18:3 [1])*	1.3951	1.0532	1.0355	0.9349	0.0044	0.0118	0.2305
469		palmitoyl-myristoyl-glycerol (16:0/14:0) [1]*	0.5331	1.6607	0.4686	1.0718	0.0011	0.0000	0.0411
470		palmitoyl-myristoyl-glycerol (16:0/14:0) [2]	0.6336	1.2293	0.5853	0.8974	0.0367	0.0000	0.1231
471		palmitoyl-palmitoyl-glycerol (16:0/16:0) [1]*	0.5136	1.5408	0.5020	1.0977	0.0411	0.0000	0.0307
472		palmitoyl-palmitoyl-glycerol (16:0/16:0) [2]*	1.0246	1.1140	1.2131	0.8928	0.6223	0.0910	0.0015
473		palmitoleoyl-palmitoleoyl-glycerol (16:1/16:1) [2]*	0.8583	1.5914	0.5284	0.9065	0.0000	0.0000	0.4473
474		palmitoyl-oleoyl-glycerol (16:0/18:1) [1]*	0.5007	1.4283	0.4247	1.0292	0.0023	0.0000	0.1812
475		palmitoyl-oleoyl-glycerol (16:0/18:1) [2]*	0.6826	1.0885	0.6117	0.9585	0.0070	0.0000	0.7269
476		palmitoyl-linoleoyl-glycerol (16:0/18:2) [1]*	0.7694	1.3332	0.8268	1.0463	0.1812	0.0000	0.0236
477		palmitoyl-linoleoyl-glycerol (16:0/18:2) [2]*	0.9030	1.0002	0.9094	1.0031	0.8346	0.0273	0.8849
478		palmitoyl-linolenoyl-glycerol (16:0/18:3) [2]*	1.5237	0.5992	1.6390	1.0447	0.2080	0.0049	0.5138
479		palmitoleoyl-oleoyl-glycerol (16:1/18:1) [2]*	0.8144	1.4224	0.4627	0.9103	0.0000	0.0000	0.5619
480		palmitoleoyl-linoleoyl-glycerol (16:1/18:2) [1]*	1.1831	1.5156	0.8853	1.0852	0.2795	0.8791	0.7240
481		palmitoyl-arachidonoyl-glycerol (16:0/20:4) [1]*	0.5149	0.9290	0.8757	0.9564	0.1071	0.1326	0.1427
482		palmitoyl-arachidonoyl-glycerol (16:0/20:4) [2]*	1.0222	0.9289	1.2247	0.9901	0.0048	0.0008	0.1763
483	Diacylglycerol	palmitoleoyl-arachidonoyl-glycerol (16:1/20:4) [2]*	1.4642	0.8797	1.1412	1.0707	0.6620	0.0743	0.3015
484		palmitoyl-docosahexaenoyl-glycerol (16:0/22:6)	0.3473	0.9366	0.4854	0.8942	0.2287	0.0000	0.1170
485		stearoyl-linoleoyl-glycerol (18:0/18:2) [2]*	1.2097	0.9165	1.5913	1.0385	0.0603	0.0381	0.2842
486		oleoyl-oleoyl-glycerol (18:1/18:1) [1]*	0.7070	1.5849	0.5868	1.0359	0.0009	0.0000	0.1127

487		oleoyl-oleoyl-glycerol (18:1/18:1) [2]*	0.8438	1.1979	0.7031	0.9817	0.0005	0.0000	0.8578			
488		oleoyl-linoleoyl-glycerol (18:1/18:2) [1]	1.1951	1.1549	1.2885	1.0241	0.6775	0.1110	0.2110			
489		oleoyl-linoleoyl-glycerol (18:1/18:2) [2]	1.3986	0.9841	1.2791	1.0427	0.7390	0.0006	0.3840			
490		oleoyl-linolenoyl-glycerol (18:1/18:3) [2]*	2.1847	0.1804	1.6211	0.2297	0.7689	0.0000	0.4679			
491		linoleoyl-linoleoyl-glycerol (18:2/18:2) [1]*	2.3975	0.8482	3.4093	1.2799	0.0040	0.0000	0.9790			
492		linoleoyl-linoleoyl-glycerol (18:2/18:2) [2]*	2.9655	0.5941	3.9381	1.0073	0.0646	0.0000	0.8123			
493		linoleoyl-linolenoyl-glycerol (18:2/18:3) [1]*	2.5133	0.6504	3.1809	1.0846	0.0413	0.0000	0.2878			
494		linoleoyl-linolenoyl-glycerol (18:2/18:3) [2]*	2.5552	0.7798	2.3231	1.1247	0.3267	0.0000	0.1282			
495		stearoyl-arachidonoyl-glycerol (18:0/20:4) [1]*	1.0131	1.2670	1.8491	0.8060	0.5402	0.0135	0.0001			
496		stearoyl-arachidonoyl-glycerol (18:0/20:4) [2]*	1.8889	0.9452	2.2045	0.9774	0.1659	0.0000	0.3176			
497		oleoyl-arachidonoyl-glycerol (18:1/20:4) [1]*	0.1894	1.3737	0.6674	1.0337	0.0018	0.0000	0.0000			
498		oleoyl-arachidonoyl-glycerol (18:1/20:4) [2]*	0.9942	1.0334	0.9008	0.9797	0.1556	0.2344	0.7019			
499		linoleoyl-arachidonoyl-glycerol (18:2/20:4) [1]*	1.2300	0.7964	1.8922	1.0974	0.1061	0.0632	0.6041			
500		linoleoyl-arachidonoyl-glycerol (18:2/20:4) [2]*	1.4009	0.6575	1.7401	0.8861	0.0558	0.0000	0.9886			
501		stearoyl-docosahexaenoyl-glycerol (18:0/22:6)	1.2586	0.8379	1.6927	1.0002	0.0005	0.0000	0.2991			
502		linoleoyl-docosahexaenoyl-glycerol (18:2/22:6)	2.3047	0.7709	2.3535	1.1578	0.0501	0.0000	0.0697			
503		sphinganine	0.5365	1.2512	1.1221	1.1474	0.0021	0.0000	0.0002			
504	Sphingolipid Synthesis	sphingadienine	1.0391	1.1735	1.1547	1.0314	0.8056	0.9263	0.0805			
505		phytosphingosine	0.7357	1.0660	0.8760	1.2325	0.0721	0.0013	0.5401			
506	Dihydroceramides	N-palmitoyl-sphinganine (d18:0/16:0)	0.7167	1.0954	1.0181	1.0016	0.1030	0.0103	0.0086			
507		N-stearoyl-sphinganine (d18:0/18:0)*	0.2701	1.4104	0.4583	0.9573	0.5299	0.0000	0.0010			
508		N-palmitoyl-sphingosine (d18:1/16:0)	0.7427	1.1382	1.0531	0.9293	0.3897	0.0699	0.0029			
509		N-(2-hydroxypalmitoyl)-sphingosine (d18:1/16:0(2OH))	0.7891	2.0115	0.9034	1.2472	0.2670	0.0006	0.0395			
510		N-stearoyl-sphingosine (d18:1/18:0)*	0.5279	1.2507	0.5863	1.0965	0.7970	0.0000	0.1220			
511		N-palmitoyl-sphingadienine (d18:2/16:0)*	0.8306	1.0470	1.1652	1.0144	0.0955	0.4482	0.0418			
512		N-behenoyl-sphingadienine (d18:2/22:0)*	1.4599	0.6386	1.3170	0.8585	0.2730	0.0000	0.0555			
513	Ceramides	N-palmitoyl-heptadecasphingosine (d17:1/16:0)*	0.6347	1.0418	0.9143	0.9346	0.1727	0.0067	0.0101			
514		ceramide (d18:1/14:0, d16:1/16:0)*	0.3622	1.0996	0.4542	1.0156	0.5163	0.0000	0.0766			
515		ceramide (d18:1/17:0, d17:1/18:0)*	0.5588	0.9734	0.7531	1.0825	0.0069	0.0000	0.1398			
516		ceramide (d18:1/20:0, d16:1/22:0, d20:1/18:0)*	0.7216	0.9338	0.6243	1.0056	0.6966	0.0000	0.0926			
517		ceramide (d16:1/24:1, d18:1/22:1)*	1.2098	0.9215	0.9972	0.9267	0.3169	0.0326	0.2572			
518		ceramide (d18:2/24:1, d18:1/24:2)*	1.3395	0.9986	1.7283	1.0045	0.0269	0.0000	0.0350			
519		glycosyl-N-palmitoyl-sphingosine (d18:1/16:0)	1.0640	1.0310	1.5994	0.9748	0.0273	0.0015	0.0052			
520		glycosyl-N-stearoyl-sphingosine (d18:1/18:0)	0.5618	1.3046	0.6719	1.1131	0.7147	0.0000	0.1083			
521	Hexosylce	glycosyl-N-behenoyl-sphingadienine (d18:2/22:0)*	1.3756	0.4997	1.4206	0.9026	0.0839	0.0005	0.0801			

522	Hexosylceramides (HCER)	glycosyl ceramide (d18:1/20:0, d16:1/22:0)*	0.7379	0.8466	0.7717	1.0394	0.0582	0.0032	0.2949			
523		glycosyl ceramide (d16:1/24:1, d18:1/22:1)*	1.3526	0.8138	1.5378	0.7965	0.5526	0.0000	0.3444			
524		glycosyl ceramide (d18:1/23:1, d17:1/24:1)*	1.4734	0.5125	2.8411	0.6524	0.0035	0.0000	0.1053			
525		glycosyl ceramide (d18:2/24:1, d18:1/24:2)*	1.6794	1.0506	3.0063	1.0466	0.0017	0.0000	0.0021			
526	Lactosylceramides (LCER)	lactosyl-N-palmitoyl-sphingosine (d18:1/16:0)	0.6946	1.3122	1.1130	1.0084	0.2545	0.0112	0.0007			
527		lactosyl-N-nervonoyl-sphingosine (d18:1/24:1)*	1.3398	1.2948	2.3051	0.8675	0.7527	0.0003	0.0009			
528	Dihydrosphingomyelins	palmitoyl dihydrosphingomyelin (d18:0/16:0)*	1.0112	0.9036	1.4149	1.0822	0.0002	0.0022	0.1546			
529		behenoyl dihydrosphingomyelin (d18:0/22:0)*	0.9756	0.8350	0.9754	1.0387	0.3439	0.4344	0.3700			
530		sphingomyelin (d18:0/18:0, d19:0/17:0)*	0.8006	0.8519	1.1858	1.1370	0.0008	0.9991	0.4648			
531		sphingomyelin (d18:0/20:0, d16:0/22:0)*	0.5736	0.5974	0.5653	0.7929	0.5320	0.3485	0.5130			
532	Sphingomyelins	palmitoyl sphingomyelin (d18:1/16:0)	0.9072	1.0661	1.0367	1.1119	0.0672	0.0173	0.2968			
533		stearoyl sphingomyelin (d18:1/18:0)	0.7961	1.0688	0.8078	1.1652	0.4711	0.0000	0.6723			
534		behenoyl sphingomyelin (d18:1/22:0)*	2.4628	0.7113	2.3094	1.0573	0.2933	0.0000	0.1541			
535		tricosanoyl sphingomyelin (d18:1/23:0)*	1.5547	0.9549	1.9508	0.9985	0.0611	0.0000	0.1530			
536		lignoceroyl sphingomyelin (d18:1/24:0)	2.1810	0.8401	2.3704	0.9627	0.0539	0.0000	0.6687			
537		sphingomyelin (d18:2/23:1)*	2.4390	0.7564	3.3798	1.0037	0.0384	0.0000	0.8372			
538		sphingomyelin (d18:2/24:2)*	1.1157	0.3007	2.0217	0.4988	0.0046	0.0000	0.3804			
539		sphingomyelin (d18:1/14:0, d16:1/16:0)*	0.7426	1.0647	0.7177	1.2370	0.5967	0.0000	0.3429			
540		sphingomyelin (d17:1/16:0, d18:1/15:0,	1.1219	0.9920	1.1933	1.2543	0.2021	0.5967	0.4462			
541		sphingomyelin (d18:2/16:0, d18:1/16:1)*	1.7369	0.8223	1.9739	1.1215	0.0353	0.0000	0.3885			
542		sphingomyelin (d18:1/17:0, d17:1/18:0,	0.8347	0.9238	0.9948	1.2447	0.0011	0.0201	0.4037			
543		sphingomyelin (d18:1/18:1, d18:2/18:0)	0.9076	1.0315	0.9111	1.2644	0.2494	0.0171	0.3016			
544		sphingomyelin (d18:1/19:0, d19:1/18:0)*	1.3093	0.7345	1.2745	1.1357	0.0606	0.0008	0.0441			
545		sphingomyelin (d18:1/20:0, d16:1/22:0)*	1.1132	0.7271	1.0552	1.0610	0.1303	0.0174	0.0516			
546		sphingomyelin (d18:1/20:1, d18:2/20:0)*	1.6475	0.7903	1.2558	1.1428	0.8750	0.0094	0.1625			
547		sphingomyelin (d18:1/21:0, d17:1/22:0,	2.1927	0.6428	2.0729	1.0918	0.1166	0.0000	0.0613			
548		sphingomyelin (d18:2/21:0, d16:2/23:0)*	2.5133	0.5771	2.1162	0.9469	0.3655	0.0000	0.0674			
549		sphingomyelin (d18:1/22:1, d18:2/22:0,	2.1586	0.6831	1.8901	0.9486	0.6166	0.0000	0.1380			
550		sphingomyelin (d18:1/22:2, d18:2/22:1,	1.9258	0.7190	1.9742	1.1152	0.0834	0.0000	0.1260			
551		sphingomyelin (d18:2/23:0, d18:1/23:1,	2.1953	0.8468	2.6959	1.0205	0.0574	0.0000	0.7467			
552		sphingomyelin (d18:1/24:1, d18:2/24:0)*	2.0190	0.9851	2.6071	1.0174	0.0962	0.0000	0.1220			
553		sphingomyelin (d18:2/24:1, d18:1/24:2)*	2.0796	0.9350	2.7988	1.1316	0.0052	0.0000	0.4317			
554	Sphingosines	sphingosine	0.8482	1.3939	1.0381	1.1032	0.7377	0.0000	0.0005			
555		hexadecasphingosine (d16:1)*	0.3773	1.0507	0.4951	1.0845	0.1562	0.0000	0.1356			
556		heptadecasphingosine (d17:1)	0.9201	1.2441	1.0494	1.0584	0.8563	0.0352	0.0315			

557		Mevalonate Metabolism	3-hydroxy-3-methylglutarate	1.5089	0.8593	1.5283	0.9126	0.7286	0.0002	0.9073			
558		Sterol	cholesterol	1.1222	1.0185	1.4237	1.0726	0.0056	0.0005	0.0788			
559			7-alpha-hydroxy-3-oxo-4-cholestenoate (7-Hoca)	1.1283	0.9943	1.0395	0.8959	0.6683	0.4988	0.7767			
560			4-cholesten-3-one	0.1881	0.9736	0.1471	1.1219	0.5665	0.0000	0.0742			
561			beta-sitosterol	0.7925	0.3923	1.1884	0.3923	0.0080	0.0000	0.0080			
562			campesterol	1.2579	0.0473	1.5459	0.0522	0.0515	0.0000	0.3032			
563			7-hydroxycholesterol (alpha or beta)	1.1433	0.7409	0.5354	0.9184	0.5296	0.5672	0.0615			
564		Corticosteroids	corticosterone	0.8175	0.9969	1.7478	0.7370	0.3309	0.1484	0.0634			
565		Primary Bile Acid Metabolism	cholate	2.6422	1.0593	4.2857	0.9891	0.4169	0.0001	0.1949			
566			glycocholate	1.6261	1.9767	2.7500	1.6592	0.5075	0.8261	0.1388			
567			taurocholate	2.1493	1.4780	2.4960	0.8444	0.6879	0.0310	0.0817			
568			chenodeoxycholate	2.2256	0.9740	2.0255	0.8051	0.7778	0.0022	0.6138			
569			taurochenodeoxycholate	3.3322	2.0412	2.3849	0.7314	0.0986	0.2403	0.0911			
570			beta-muricholate	2.0703	1.3391	1.9693	0.8068	0.2491	0.0275	0.2224			
571			alpha-muricholate	2.4162	0.8285	3.2329	0.8968	0.4414	0.0003	0.4102			
572			tauro-beta-muricholate	4.4409	2.4703	3.7729	0.8096	0.2829	0.1300	0.1254			
573		Secondary Bile Acid Metabolism	deoxycholate	3.9509	0.8350	7.1719	0.8875	0.0584	0.0000	0.4785			
574			taurodeoxycholate	4.6415	1.0048	7.7647	0.9302	0.2653	0.0000	0.1079			
575			6-beta-hydroxylithocholate	5.4927	0.9807	6.3152	0.9143	0.7782	0.0000	0.6312			
576			taurolithocholate	4.6019	0.8267	4.1647	0.4696	0.1546	0.0000	0.1389			
577			ursodeoxycholate	2.5573	0.8972	2.5127	0.7859	0.9750	0.0002	0.3863			
578			tauroursodeoxycholate	4.5764	1.8216	3.4998	0.8336	0.3167	0.0518	0.1522			
579			6-oxolithocholate	3.3158	0.2052	4.1161	0.1996	0.6557	0.0000	0.6194			
580			hyodeoxycholate	10.3182	0.8409	10.7790	1.0473	0.3422	0.0000	0.9618			
581			taurohyodeoxycholic acid	11.0026	1.5554	6.6985	0.6180	0.2871	0.0176	0.4466			
582			3-dehydrocholate	1.8649	0.9428	4.0509	0.7336	0.2410	0.0003	0.1462			
583			7-ketodeoxycholate	8.0595	0.8369	9.1963	0.8945	0.5667	0.0000	0.6890			
584			ursocholate	12.4682	0.3947	11.5878	0.6302	0.1590	0.0000	0.1448			
585		Purine Metabolism, (Hypo)Xanthine/Inosine containing	inosine	1.4787	0.5018	0.8401	1.0252	0.3448	0.0003	0.0000			
586			hypoxanthine	1.2683	0.7422	1.6000	0.9911	0.0001	0.0000	0.7012			
587			xanthine	1.0628	0.9850	1.2964	1.0200	0.0002	0.0000	0.0038			
588			xanthosine	1.4316	0.9949	1.6522	1.1594	0.3523	0.0076	0.8181			
589			2'-deoxyinosine	1.2374	1.5616	2.1756	1.0280	0.3899	0.0386	0.0027			
590			urate	1.2188	0.9731	1.7944	0.9463	0.1500	0.0134	0.6726			
591			uric acid ribonucleoside*	0.3748	1.4953	1.6306	2.0843	0.0003	0.0004	0.0031			
592			allantoin	1.0201	1.1190	1.2286	0.9938	0.3398	0.1831	0.0010			
593			allantoic acid	1.8104	0.7337	1.9320	1.1613	0.0254	0.0000	0.1203			

594	Nucleotide	Purine Metabolism, Adenine containing	adenosine 5'-diphosphate (ADP)	1.1825	0.7027	1.5470	0.9970	0.0228	0.0022	0.7165
595			adenosine 5'-monophosphate (AMP)	1.1445	0.7559	1.8009	0.9664	0.0002	0.0000	0.2449
596			adenosine 3'-monophosphate (3'-AMP)	1.2896	1.4380	2.9490	1.4880	0.0174	0.0855	0.0265
597			adenosine 2'-monophosphate (2'-AMP)	1.2902	1.0681	1.0814	1.2431	0.7098	0.9219	0.0497
598			adenosine 3',5'-diphosphate	1.3620	0.5668	0.9880	1.3420	0.0595	0.3249	0.0132
599			adenylosuccinate	2.1848	2.7819	2.6260	2.3193	0.3339	0.5717	0.6558
600			adenosine	1.7497	0.7612	1.1716	1.0594	0.7357	0.0000	0.0007
601			adenine	0.6798	1.2691	0.7849	1.1432	0.7481	0.0000	0.1695
602			N1-methyladenosine	1.0651	0.9312	2.0178	1.0551	0.0122	0.0036	0.0487
603			N6-carbamoylthreonyladenosine	1.4853	0.8648	2.5401	1.1451	0.0026	0.0000	0.3032
604			2'-deoxyadenosine 5'-monophosphate	2.5402	0.7155	5.9631	0.9990	0.0029	0.0000	0.1622
605			2'-deoxyadenosine 3'-monophosphate	0.9695	4.1171	3.2105	1.6374	0.2964	0.0326	0.0000
606			N6-succinyladenosine	2.0294	1.3319	1.4385	1.0147	0.5407	0.4444	0.3440
607		Purine Metabolism, Guanine containing	guanosine 5'-diphosphate (GDP)	1.4051	0.5153	1.8252	0.7814	0.0569	0.0001	0.9863
608			guanosine 5'-monophosphate (5'-GMP)	1.1552	0.4525	1.0324	0.5099	0.8020	0.0010	0.8295
609			guanosine	1.1238	0.7738	1.0627	1.0479	0.1491	0.0435	0.0753
610			guanine	1.2631	1.5499	1.9058	0.9618	0.9220	0.0158	0.0002
611			7-methylguanine	1.5442	0.8255	2.2854	1.2269	0.0013	0.0000	0.9981
612			N2,N2-dimethylguanosine	0.9166	0.3508	2.2933	0.5897	0.0028	0.0000	0.2960
613			2'-deoxyguanosine	1.0147	1.5161	1.9945	1.1516	0.0470	0.3724	0.0002
614			guanosine 2'-monophosphate (2'-GMP)*	1.2090	1.4047	1.5963	1.2838	0.9413	0.7893	0.2239
615		Pyrimidine Metabolism, Orotate containing	orotate	3.4110	0.8123	3.9239	0.8181	0.9176	0.0000	0.6409
616			orotidine	1.0552	1.0673	1.2318	1.0786	0.3064	0.3508	0.3348
617		Pyrimidine Metabolism, Uracil containing	uridine 5'-monophosphate (UMP)	1.0621	0.8622	1.8926	0.8281	0.1948	0.0056	0.1220
618			uridine 3'-monophosphate (3'-UMP)	1.3975	1.4164	1.9758	1.1638	0.7344	0.0270	0.0185
619			uridine-2',3'-cyclic monophosphate	1.6577	0.8908	1.3059	1.3814	0.5572	0.0574	0.0821
620			uridine	1.4788	0.6861	1.4532	0.8214	0.4036	0.0000	0.2193
621			uracil	0.8096	1.1386	1.7258	1.0100	0.0109	0.4628	0.0005
622			pseudouridine	1.3085	1.0482	2.7075	0.9582	0.0140	0.0000	0.0017
623			5,6-dihydrouridine	1.3321	1.0934	2.0049	0.9513	0.3197	0.0003	0.0234
624			2'-O-methyluridine	0.9958	0.6188	1.0689	1.0080	0.0218	0.0298	0.0610
625			5-methyluridine (ribothymidine)	1.8545	0.6012	2.6405	0.8203	0.0010	0.0000	0.8297
626			2'-deoxyuridine	1.3580	0.9298	2.0594	0.9258	0.1737	0.0001	0.1796
627			3-ureidopropionate	0.0120	4.2368	0.0115	0.7167	0.0012	0.0000	0.0008
628			beta-alanine	0.8266	1.2279	1.9551	1.4090	0.0006	0.8609	0.0038

629		Pyrimidine Metabolism, Cytidine containing	cytidine diphosphate	1.5603	0.4901	1.3440	1.0702	0.0947	0.0053	0.0684			
630			cytidine 5'-monophosphate (5'-CMP)	1.6207	0.7124	1.5333	0.8727	0.3014	0.0000	0.1213			
631			cytidine	1.6270	0.7388	1.8070	1.0088	0.0783	0.0000	0.4721			
632			cytosine	0.4513	0.9703	0.5611	0.5014	0.1318	0.0416	0.0113			
633			3-methylcytidine	1.6761	1.0304	1.5048	1.0442	0.6207	0.0006	0.6789			
634			5-methylcytidine	1.1875	0.3265	1.4267	0.4126	0.4502	0.0000	0.8837			
635			2'-deoxycytidine 5'-monophosphate	1.2072	1.0954	2.6916	0.6496	0.3942	0.0000	0.0005			
636			2'-deoxycytidine	1.7919	1.0779	3.1933	0.9722	0.1444	0.0000	0.0383			
637			2'-O-methylcytidine	1.2171	0.4570	1.4692	0.8439	0.0503	0.0008	0.3529			
638			5-methyl-2'-deoxycytidine	1.2599	0.5610	2.6198	0.3173	0.6449	0.0000	0.0246			
639		Pyrimidine Metabolism, Thymine containing	thymidine	1.4904	1.4802	2.6905	0.9149	0.5408	0.0002	0.0004			
640			thymine	1.2914	1.2194	2.3002	1.0435	0.1145	0.0018	0.0080			
641			3-aminoisobutyrate	1.5971	0.8261	2.0090	0.9722	0.1044	0.0000	0.6612			
642		Purine and Pyrimidine Metabolism	methylphosphate	1.6853	0.9378	1.6541	0.9912	0.7014	0.0000	0.5700			
643		Dinucleotide	(3'-5')-adenylylcytidine	2.2696	0.5506	1.8073	0.8832	0.6610	0.0000	0.0703			
644			(3'-5')-adenylyluridine	1.8977	0.3385	2.2586	0.8675	0.0589	0.0000	0.0867			
645			(3'-5')-adenylyladenosine*	2.2548	0.4872	1.5917	0.9908	0.3916	0.0000	0.0223			
646			(3'-5')-cytidylyluridine*	1.4607	1.1698	1.8920	1.1908	0.4224	0.0747	0.2465			
647			(3'-5')-guanylylcytidine	2.2207	0.5889	1.7664	0.9094	0.4787	0.0000	0.1898			
648			(3'-5')-uridylylcytidine*	1.8406	0.7251	1.4982	1.0504	0.3597	0.0000	0.0110			
649			(3'-5')-uridylyluridine	1.9816	0.3975	1.3716	1.1392	0.0258	0.0000	0.0001			
650		Nicotinate and Nicotinamide Metabolism	quinolinate	0.4163	0.8725	0.3912	0.8456	0.8127	0.0015	0.9396			
651			nicotinate	0.9137	0.7553	0.9623	0.9929	0.0511	0.2994	0.1208			
652			nicotinate ribonucleoside	0.6130	1.3754	1.2254	1.2211	0.5351	0.0859	0.2473			
653			nicotinamide	1.0411	0.8773	1.0016	1.0110	0.2796	0.0666	0.0408			
654			nicotinamide adenine dinucleotide (NAD+)	1.4018	0.7216	1.6732	1.0202	0.0005	0.0000	0.2321			
655			1-methylnicotinamide	0.2899	0.8762	0.1218	1.6649	0.5640	0.0000	0.0007			
656			trigonelline (N'-methylnicotinate)	19.1572	0.7818	23.3319	1.0372	0.2569	0.0000	0.6777			
657			N1-Methyl-2-pyridone-5-carboxamide	0.6298	0.8897	0.8477	1.3586	0.0002	0.0000	0.5329			
658		Riboflavin Metabolism	riboflavin (Vitamin B2)	1.3592	0.9542	2.3937	1.0676	0.0147	0.0000	0.0532			
659			flavin adenine dinucleotide (FAD)	1.3896	0.7688	1.8127	1.1143	0.0001	0.0000	0.6031			
660			flavin mononucleotide (FMN)	1.5203	0.8874	1.9910	1.1057	0.0242	0.0000	0.5558			
661		Pantothen	pantothenate	1.5325	0.8902	1.6604	1.0035	0.1746	0.0000	0.9113			
662			pantetheine	1.0319	0.7536	0.7309	1.0546	0.9402	0.7922	0.0295			

663		ate and CoA Metabolism	phosphopantetheine	1.4454	0.6058	0.7033	2.1647	0.6818	0.4344	0.0662			
664			3'-dephosphocoenzyme A	0.7212	0.9007	0.2287	1.4371	0.2631	0.0002	0.0219			
665			coenzyme A	0.8716	0.4638	0.4479	1.7006	0.3404	0.1295	0.0216			
666		Ascorbate and Aldarate Metabolism	ascorbate (Vitamin C)	0.5948	1.1818	0.5898	1.4026	0.9093	0.0000	0.4763			
667			dehydroascorbate	1.1490	1.0177	1.1065	1.0838	0.9897	0.3614	0.7126			
668	Cofactors and Vitamins		threonate	1.0153	0.8374	1.2397	1.2701	0.0551	0.6285	0.4757			
669			oxalate (ethanedioate)	1.3895	1.0002	1.3556	0.8732	0.3543	0.0002	0.4876			
670			gulonate*	1.7430	0.7497	1.7525	0.8637	0.7208	0.0000	0.7155			
671		Tocopherol Metabolism	alpha-tocopherol	0.5184	1.3270	0.6104	1.1449	0.9078	0.0000	0.0078			
672			gamma-tocopherol/beta-tocopherol	0.2015	1.2640	0.3222	0.8648	0.6632	0.0000	0.0000			
673		Folate Metabolism	5-methyltetrahydrofolate (5MeTHF)	0.5608	0.8474	1.1143	1.6197	0.0016	0.0834	0.8173			
674		Tetrahydrobiopterin Metabolism	biopterin	1.2143	0.8541	1.7260	0.9637	0.0139	0.0000	0.2525			
675			dihydrobiopterin	1.4289	0.8470	1.6114	1.1629	0.0548	0.0003	0.1836			
676		Pterin Metabolism	pterin	1.5813	0.8626	2.3353	1.0574	0.0193	0.0000	0.3298			
677			xanthopterin	2.1411	0.9870	2.2011	0.8283	0.3254	0.0000	0.1829			
678		Hemoglobin and Porphyrin Metabolism	heme	0.6923	1.3613	1.7512	0.7983	0.5162	0.5467	0.0966			
679			bilirubin (Z,Z)	0.4792	1.6222	0.8336	0.9606	0.4592	0.0007	0.0056			
680			biliverdin	0.7441	1.1716	1.4305	0.9614	0.0788	0.5094	0.0037			
681		Thiamine Metabolism	thiamin (Vitamin B1)	1.3307	0.8700	1.5774	1.0821	0.0002	0.0000	0.5687			
682			thiamin monophosphate	1.6176	0.8566	2.4558	1.1571	0.0006	0.0000	0.6370			
683		Vitamin A Metabolism	retinol (Vitamin A)	2.4230	0.8724	4.6266	1.4988	0.0073	0.0048	0.3628			
684			retinal	4.6912	0.7343	6.2573	1.4958	0.0032	0.0000	0.3075			
685		Vitamin B6 Metabolism	pyridoxamine	1.4760	1.1281	2.5637	1.0724	0.0224	0.0000	0.0068			
686			pyridoxamine phosphate	1.4586	0.8347	1.6452	1.0990	0.0404	0.0000	0.4036			
687			pyridoxal phosphate	1.4326	0.4897	2.4467	0.5651	0.0478	0.0000	0.1720			
688			pyridoxal	1.3872	0.9844	1.2677	0.9515	0.5122	0.0011	0.8034			
689			pyridoxate	1.5217	1.1091	1.7707	0.9318	0.9730	0.0001	0.1176			
690		Benzoate Metabolism	hippurate	4.5036	1.7077	6.1604	1.0165	0.7988	0.0000	0.0400			
691			4-hydroxyhippurate	1.0462	0.8983	1.0874	0.7084	0.9670	0.0829	0.5359			
692			catechol sulfate	0.7806	0.2538	1.0985	0.2538	0.0858	0.0000	0.0858			
693			p-cresol sulfate	0.5640	0.8949	2.2679	2.0423	0.0000	0.3105	0.0644			
694			phenylpropionylglycine	1.1321	0.0295	1.2825	0.0295	0.3775	0.0000	0.3775			
695		Food Component/Plant	2,3-dihydroxyisovalerate	2.3793	0.7544	2.8313	0.9256	0.1604	0.0000	0.9945			
696			2,8-quinolinediol	0.8998	0.4183	0.8826	0.4183	0.7244	0.0000	0.7244			
697			gluconate	1.5906	0.7479	0.7077	1.2429	0.6713	0.8928	0.0007			
698			beta-guanidinopropanoate	2.6594	1.0467	2.7383	1.0454	0.9455	0.0000	0.6922			
699			cinnamoylglycine	0.7188	0.2628	1.1825	0.2628	0.0244	0.0000	0.0244			
700			enterolactone	3.9272	0.2281	1.0208	0.2281	0.0111	0.0000	0.0111			
701			ergothioneine	48.4861	0.7012	58.2021	1.0424	0.0014	0.0000	0.1756			

702	Xenobiotics		erythritol	0.8986	0.9980	1.0104	1.1571	0.2596	0.1935	0.6397			
703			kojibiose	1.5301	1.0300	0.3906	1.0215	0.0004	0.0152	0.0004			
704			stachydrine	5.5206	0.7374	16.9947	0.6776	0.0107	0.0000	0.0045			
705			tartarate	0.0497	0.8246	0.0434	2.3004	0.2779	0.0000	0.1885			
706			methyl glucopyranoside (alpha + beta)	1.9493	0.8953	0.7890	1.1113	0.0002	0.0334	0.0000			
707			tartronate (hydroxymalonate)	1.5289	0.8775	1.5211	0.8720	0.8512	0.0000	0.9543			
708		Drug - Analgesics, Anesthetic	lidocaine	0.2573	0.9189	0.2692	0.2573	0.1415	0.1415	0.1022			
709			N-ethylglycinexylidide	1.0000	1.0000	1.0000	1.0000	1.0000	1.0000	1.0000			
710		Chemical	sulfate*	1.2923	0.8107	1.3369	0.9872	0.1560	0.0000	0.3258			
711			S-(3-hydroxypropyl)mercapturic acid (HPMA)	1.1774	0.2665	2.5440	0.4513	0.0009	0.0000	0.1209			
712			perfluorooctanesulfonate (PFOS)	8.1501	0.4964	7.0638	0.7945	0.0534	0.0000	0.0009			
713			3-hydroxypyridine sulfate	1.3336	0.2168	1.8147	0.2168	0.0163	0.0000	0.0163			
714			thioproline	1.7371	0.9300	1.6227	1.6748	0.7239	0.4417	0.3255			

0.0000 means <0.0001

Summary

Two-Way ANOVA	Ucn2 Effect	Diet Effect	Interaction
**Total biochemicals $p \leq 0.05$**	292	501	162
**Total biochemicals $0.05 < p < 0.10$**	66	32	60
