# Supplementary material for: Significant alteration of liver metabolites by AAV8.Urocortin 2 gene transfer in mice with insulin resistance
Source: PLoS One. 2019 Dec 2;14(12):e0224428. doi: 10.1371/journal.pone.0224428 (PMC6886859; doi:10.1371/journal.pone.0224428)
Supplement: S5 Table — (PDF) [file pone.0224428.s006.pdf]

Supplementary Table 5. AAV8.Empt altered metabolites in HFD mice liver				
Number	Super Pathway	Sub Pathway	Biochemical Name	AAV8.Empt vs Saline
1	Amino Acid	Glycine, Serine and Threonine Metabolism	N-acetylglycine	0.72
2			sarcosine	0.55
3			dimethylglycine	0.71
4			betaine	0.66
5			serine	0.84
6			threonine	0.81
7		Alanine and Aspartate Metabolism	N-acetylalanine	0.78
8			aspartate	0.83
9			asparagine	0.83
10		Glutamate Metabolism	S-1-pyrroline-5-carboxylate	1.96
11		Histidine Metabolism	histidine	0.90
12			3-methylhistidine	0.57
13			imidazole propionate	2.48
14			formiminoglutamate	0.32
15			imidazole lactate	1.42
16			1-ribosyl-imidazoleacetate*	2.83
17		Lysine Metabolism	lysine	0.84
18			N2-acetyllysine	0.65
19			N6-acetyllysine	0.62
20			N6,N6,N6-trimethyllysine	0.78
21			5-(galactosylhydroxy)-L-lysine	0.80
22			5-aminovalerate	1.89
23			N-trimethyl 5-aminovalerate	0.58
24		Phenylalanine Metabolism	phenylalanine	0.81
25			phenylpyruvate	0.53
26		Tyrosine Metabolism	tyrosine	0.89
27			3-(4-hydroxyphenyl)lactate	0.78
28		Tryptophan Metabolism	tryptophan	0.85
29			kynurenine	0.73
30		Leucine, Isoleucine and Valine Metabolism	leucine	0.79
31			4-methyl-2-oxopentanoate	0.58
32			beta-hydroxyisovalerate	0.75
33			isoleucine	0.80
34			valine	0.75
35			3-methyl-2-oxobutyrate	0.62
36		Methionine, Cysteine, SAM and Taurine Metabolism	methionine	0.80
37			methionine sulfoxide	0.67
38			N-acetylmethionine sulfoxide	0.51
39			S-adenosylhomocysteine (SAH)	1.46
40			S-methylcysteine	0.72
41			cystine	0.33
42			arginine	0.73
43			urea	0.73

44		Urea cycle; Arginine and Proline Metabolism	ornithine	0.81
45			homocitrulline	2.62
46			proline	0.82
47			dimethylarginine (SDMA + ADMA)	0.79
48			N-alpha-acetylornithine	0.51
49		Polyamine Metabolism	putrescine	0.51
50			spermidine	0.76
51			(N(1) + N(8))-acetylspermidine	0.74
52			glutathione, reduced (GSH)	6.74
53			glutathione, oxidized (GSSG)	1.24
54			cysteine-glutathione disulfide	0.63
55			4-hydroxy-nonenal-glutathione	2.33
56	Peptide	Gamma-glutamyl Amino Acid	gamma-glutamylleucine	1.23
57			gamma-glutamyl-epsilon-lysine	0.76
58		Dipeptide	glycylleucine	0.67
59			glycylvaline	0.62
60			tyrosylglycine	0.58
61	Carbohydrate	Glycolysis, Gluconeogenesis, and Pyruvate Metabolism	glucose	1.17
62			glucose 6-phosphate	1.38
63			3-phosphoglycerate	1.27
64			phosphoenolpyruvate (PEP)	1.31
65			lactate	0.91
66		Pentose Phosphate Pathway	6-phosphogluconate	1.45
67			sedoheptulose-7-phosphate	1.70
68		Pentose Metabolism	ribitol	1.42
69			sedoheptulose	1.83
70		Glycogen Metabolism	maltopentaose	4.35
71			maltotetraose	2.92
72			maltotriose	2.04
73		Fructose, Mannose and Galactose Metabolism	fructose	1.67
74			mannitol/sorbitol	1.94
75			mannose	1.42
76		Nucleotide Sugar	UDP-N-acetylglucosamine/galactosamine	1.47
77		Aminosugar Metabolism	N-acetylglucosamine/N-acetylgalactosamine	0.55
78		Long Chain Fatty Acid	myristate (14:0)	0.40
79			myristoleate (14:1n5)	0.59
80			pentadecanoate (15:0)	0.64
81			palmitate (16:0)	0.64
82			palmitoleate (16:1n7)	0.45
83			margarate (17:0)	0.39
84			10-heptadecenoate (17:1n7)	0.37
85			stearate (18:0)	0.55
86			oleate/vaccenate (18:1)	0.58
87			nonadecanoate (19:0)	0.44
88			10-nonadecenoate (19:1n9)	0.36
89			arachidate (20:0)	0.49
90			eicosenoate (20:1)	0.39

91			erucate (22:1n9)	0.41
92		Polyunsaturated Fatty Acid (n3 and n6)	heneicosapentaenoate (21:5n3)	0.44
93			hexadecadienoate (16:2n6)	0.52
94			stearidonate (18:4n3)	0.52
95			eicosapentaenoate (EPA; 20:5n3)	0.38
96			docosapentaenoate (n3 DPA; 22:5n3)	0.35
97			docosahexaenoate (DHA; 22:6n3)	0.51
98			docosatrienoate (22:3n3)	0.48
99			nisinate (24:6n3)	0.44
100			linoleate (18:2n6)	0.56
101			linolenate [alpha or gamma; (18:3n3 or 6)]	0.40
102			dihomo-linolenate (20:3n3 or n6)	0.50
103			arachidonate (20:4n6)	0.64
104			adrenate (22:4n6)	0.42
105			docosapentaenoate (n6 DPA; 22:5n6)	0.53
106			docosadienoate (22:2n6)	0.36
107			dihomo-linoleate (20:2n6)	0.34
108			mead acid (20:3n9)	0.51
109			docosatrienoate (22:3n6)*	0.42
110		Fatty Acid, Branched	15-methylpalmitate (i17:0)	0.45
111			17-methylstearate (i19:0)	0.36
112		Fatty Acid Metabolism (also BCAA Metabolism)	methylmalonate (MMA)	0.75
113		Ketone Bodies	3-hydroxybutyrate (BHBA)	0.76
114		Fatty Acid, Monohydroxy	3-hydroxyoleate*	0.57
115		Endocannabinoid	oleoyl ethanolamide	0.75
116			N-arachidonoyltaurine	0.46
117			N-oleoyltaurine	0.28
118			N-stearoyltaurine	0.32
119			N-palmitoyltaurine	0.19
120			N-palmitoleoyltaurine*	0.42
121			N-linoleoyltaurine*	0.41
122			linoleoyl ethanolamide	0.39
123		Phospholipid Metabolism	glycerophosphorylcholine (GPC)	0.84
124	Lipid		phosphoethanolamine	0.75
125			glycerophosphoethanolamine	0.77
126		Phosphatidylcholine (PC)	1,2-dipalmitoyl-GPC (16:0/16:0)	0.91
127		Phosphatidylethanolamine (PE)	1,2-dipalmitoyl-GPE (16:0/16:0)*	0.80
128			1-palmitoyl-2-stearoyl-GPE (16:0/18:0)*	0.77
129			1-palmitoyl-2-oleoyl-GPE (16:0/18:1)	0.82
130			1-oleoyl-2-arachidonoyl-GPE (18:1/20:4)*	0.90
131			1-oleoyl-2-docosahexaenoyl-GPE (18:1/22:6)*	0.85
132		Phosphatidylglycerol (PG)	1-palmitoyl-2-oleoyl-GPG (16:0/18:1)	1.09
133		Phosphatidylinositol (PI)	1-oleoyl-2-arachidonoyl-GPI (18:1/20:4) *	0.85
134			2-palmitoyl-GPC (16:0)*	0.50
135			1-stearoyl-GPC (18:0)	1.11
136			1-oleoyl-GPC (18:1)	0.85

137		Lysophospholipid	1-linoleoyl-GPC (18:2)	0.45
138			1-arachidonoyl-GPC (20:4n6)*	0.39
139			2-stearoyl-GPE (18:0)*	0.50
140			1-arachidonoyl-GPE (20:4n6)*	0.46
141			1-palmitoyl-GPS (16:0)*	0.58
142			1-oleoyl-GPS (18:1)	0.36
143			1-palmitoyl-GPG (16:0)*	0.46
144			1-stearoyl-GPG (18:0)	0.59
145			1-oleoyl-GPG (18:1)*	0.17
146			1-linoleoyl-GPG (18:2)*	0.43
147			1-palmitoyl-GPI (16:0)	0.45
148			1-stearoyl-GPI (18:0)	0.52
149			1-oleoyl-GPI (18:1)*	0.29
150			1-arachidonoyl-GPI (20:4)*	0.48
151		Plasmalogen	1-(1-enyl-palmitoyl)-2-oleoyl-GPE (P-16:0/18:1)*	0.78
152			1-(1-enyl-palmitoyl)-2-arachidonoyl-GPE (P-16:0/20:4)*	0.86
153		Glycerolipid Metabolism	glycerol	0.78
154		Monoacylglycerol	1-myristoylglycerol (14:0)	0.44
155			1-palmitoylglycerol (16:0)	0.65
156			1-palmitoleoylglycerol (16:1)*	0.43
157			1-margaroylglycerol (17:0)	0.33
158			1-stearoylglycerol (18:0)	0.68
159			2-palmitoylglycerol (16:0)	0.42
160		Diacylglycerol	diacylglycerol (16:1/18:2 [2], 16:0/18:3 [1])*	0.85
161			palmitoleoyl-palmitoleoyl-glycerol (16:1/16:1) [2]*	0.83
162			palmitoleoyl-linoleoyl-glycerol (16:1/18:2) [1]*	0.30
163			oleoyl-oleoyl-glycerol (18:1/18:1) [1]*	0.73
164			oleoyl-oleoyl-glycerol (18:1/18:1) [2]*	0.83
165		Sphingolipid Synthesis	sphinganine	0.84
166			sphingadienine	0.76
167		Hexosylceramides (HCER)	glycosyl ceramide (d18:1/20:0, d16:1/22:0)*	1.15
168		Sphingomyelins	tricosanoyl sphingomyelin (d18:1/23:0)*	0.85
169		Sphingosines	sphingosine	0.78
170			hexadecasphingosine (d16:1)*	0.81
171			heptadecasphingosine (d17:1)	0.73
172		Purine Metabolism, (Hypo)Xanthine/Inosine containing	inosine	1.75
173			hypoxanthine	1.21
174			xanthosine	0.60
175			2'-deoxyinosine	0.59
176			uric acid ribonucleoside*	0.52
177			allantoin	0.81
178		Purine Metabolism, Adenine containing	adenosine 3'-monophosphate (3'-AMP)	0.68
179			adenosine 3',5'-diphosphate	1.54
180			2'-deoxyadenosine 3'-monophosphate	0.30
181			N6-succinyladenosine	0.69
182		Purine Metabolism, Guanine containing	guanosine	1.65
183			guanine	0.63

184	Nucleotide	Purine Metabolism, Guanine containing	7-methylguanine	1.19
185			guanosine 2'-monophosphate (2'-GMP)*	0.60
186		Pyrimidine Metabolism, Uracil containing	uridine 3'-monophosphate (3'-UMP)	0.62
187			uridine	1.33
188			uracil	0.65
189			pseudouridine	0.62
190			5,6-dihydrouridine	0.66
191			5-methyluridine (ribothymidine)	1.27
192			beta-alanine	0.71
193		Pyrimidine Metabolism, Cytidine containing	cytidine 5'-monophosphate (5'-CMP)	1.27
194			2'-deoxycytidine 5'-monophosphate	0.48
195			2'-deoxycytidine	0.64
196			5-methyl-2'-deoxycytidine	0.39
197		Pyrimidine Metabolism, Thymine containing	thymidine	0.55
198			thymine	0.65
199		Dinucleotide	(3'-5')-uridylyluridine	2.31
200			(3'-5')-cytidylyluridine*	0.66
201			(3'-5')-uridylylcytidine*	1.28
202	Cofactors and Vitamins	Nicotinate and Nicotinamide Metabolism	1-methylnicotinamide	1.46
203		Riboflavin Metabolism	riboflavin (Vitamin B2)	0.72
204			flavin adenine dinucleotide (FAD)	1.13
205		Pantothenate and CoA Metabolism	phosphopantetheine	4.33
206			3'-dephosphocoenzyme A	2.08
207			coenzyme A	3.63
208			pantetheine	1.57
209	Xenobiotics	Benzoate Metabolism	hippurate	0.40

Green: indicates significant difference ($p \leq 0.05$) between the groups shown, metabolite ratio of < 1.00

Red: indicates significant difference ($p \leq 0.05$) between the groups shown; metabolite ratio of ≥ 1.00
