# Supplementary material for: Significant alteration of liver metabolites by AAV8.Urocortin 2 gene transfer in mice with insulin resistance
Source: PLoS One. 2019 Dec 2;14(12):e0224428. doi: 10.1371/journal.pone.0224428 (PMC6886859; doi:10.1371/journal.pone.0224428)
Supplement: S6 Table — (PDF) [file pone.0224428.s007.pdf]

Supplementary Table 6. AAV8.*Ucn2* altered metabolites in HFD mice liver

Nubmer	Super Pathway	Sub Pathway	Biochemical Name	AAV8.*Ucn2* vs Saline
1	Amino Acid	Glutamate Metabolism	glutamate	1.26
2			gamma-carboxyglutamate	1.25
3			glutamate, gamma-methyl ester	1.50
4			S-1-pyrroline-5-carboxylate	2.43
5		Histidine Metabolism	N-acetylhistidine	1.46
6			N-acetyl-1-methylhistidine*	2.04
7			1-methylhistamine	2.56
8			1-ribosyl-imidazoleacetate*	1.78
9			glutarylcarnitine (C5-DC)	1.71
10		Tyrosine Metabolism	O-methyltyrosine	1.75
11			p-cresol glucuronide*	3.03
12		Tryptophan Metabolism	C-glycosyltryptophan	1.38
13			5-hydroxyindoleacetate	1.38
14		Leucine, Isoleucine and Valine Metabolism	4-methyl-2-oxopentanoate	0.52
15			alpha-hydroxyisovalerate	1.29
16			3-methyl-2-oxobutyrate	0.53
17			isobutyrylcarnitine (C4)	1.76
18		Methionine, Cysteine, SAM and Taurine Metabolism	S-adenosylhomocysteine (SAH)	1.91
19			cystine	0.59
20		Urea cycle; Arginine and Proline Metabolism	homocitrulline	3.91
21			N-alpha-acetylornithine	0.48
22		Polyamine Metabolism	putrescine	0.47
23			(N(1) + N(8))-acetylspermidine	0.71
24		Guanidino and Acetamido Metabolism	guanidinosuccinate	2.46
25		Glutathione Metabolism	glutathione, oxidized (GSSG)	1.18
26			4-hydroxy-nonenal-glutathione	1.66
27	Peptide	Gamma-glutamyl Amino Acid	gamma-glutamylglutamate	1.43
28			gamma-glutamylisoleucine*	1.30
29			gamma-glutamylleucine	1.43
30			gamma-glutamyl-epsilon-lysine	0.76
31			gamma-glutamylthreonine	1.94
32		Dipeptide	glycylisoleucine	1.57
33			isoleucylglycine	1.78
34			leucylglycine	1.99
35			phenylalanylalanine	1.66
36			threonylphenylalanine	1.74
37			valylglutamine	2.07
38			valylglycine	1.88
39			valylleucine	2.54
40		Acetylated Peptides	phenylacetylglycine	1.94
41		Glycolysis, Gluconeogenesis, and Pyruvate Metabolism	glucose 6-phosphate	1.42
42			3-phosphoglycerate	1.23
43			phosphoenolpyruvate (PEP)	1.32

44	Carbohydrate	Pentose Phosphate Pathway	6-phosphogluconate	1.76
45			sedoheptulose-7-phosphate	1.57
46		Pentose Metabolism	ribitol	2.01
47			arabonate/xylonate	0.76
48			sedoheptulose	1.69
49		Fructose, Mannose and Galactose Metabolism	mannitol/sorbitol	2.43
50			galactonate	1.75
51			N-acetylglucosaminylasparagine	1.96
52	Energy	Oxidative Phosphorylation	phosphate	1.11
53		Long Chain Fatty Acid	myristate (14:0)	0.32
54			myristoleate (14:1n5)	0.50
55			pentadecanoate (15:0)	0.62
56			palmitate (16:0)	0.58
57			palmitoleate (16:1n7)	0.35
58			margarate (17:0)	0.47
59			10-heptadecenoate (17:1n7)	0.35
60			stearate (18:0)	0.56
61			oleate/vaccenate (18:1)	0.52
62			10-nonadecenoate (19:1n9)	0.35
63			eicosenoate (20:1)	0.39
64			erucate (22:1n9)	0.46
65		Polyunsaturated Fatty Acid (n3 and n6)	heneicosapentaenoate (21:5n3)	0.45
66			stearidonate (18:4n3)	0.56
67			eicosapentaenoate (EPA; 20:5n3)	0.44
68			docosapentaenoate (n3 DPA; 22:5n3)	0.41
69			docosahexaenoate (DHA; 22:6n3)	0.48
70			docosatrienoate (22:3n3)	0.53
71			nisinate (24:6n3)	0.47
72			linoleate (18:2n6)	0.59
73			linolenate [alpha or gamma; (18:3n3 or 6)]	0.44
74			dihomo-linolenate (20:3n3 or n6)	0.54
75			arachidonate (20:4n6)	0.57
76			adrenate (22:4n6)	0.41
77			docosapentaenoate (n6 DPA; 22:5n6)	0.42
78			docosadienoate (22:2n6)	0.39
79			dihomo-linoleate (20:2n6)	0.35
80			mead acid (20:3n9)	0.35
81			docosatrienoate (22:3n6)*	0.33
82		Fatty Acid, Branched	15-methylpalmitate (i17:0)	0.43
83			17-methylstearate (i19:0)	0.45
84		Fatty Acid, Dicarboxylate	3-methylglutarate/2-methylglutarate	1.57
85		Fatty Acid Metabolism(Acyl Glycine)	3,4-methylene heptanoylglycine	1.41
86		Fatty Acid Metabolism(Acyl Carnitine)	linoleoylcarnitine (C18:2)*	1.70
87			pimeloylcarnitine/3-methyladipoylcarnitine (C7-DC)	2.43
88			arachidonoylcarnitine (C20:4)	1.22
89			erucoylcarnitine (C22:1)*	0.61
90			palmitoylcholine	1.56

91		Fatty Acid Metabolism (Acyl Choline)	oleoylcholine	1.85
92			palmitoloelycholine	2.14
93			linoleoylcholine*	2.80
94			stearoylcholine*	2.08
95			arachidonoylcholine	1.65
96		Fatty Acid, Monohydroxy	2-hydroxypalmitate	0.70
97			3-hydroxyoleate*	0.48
98			12-HETE	1.59
99		Endocannabinoid	N-arachidonoyltaurine	0.57
100			N-oleoyltaurine	0.28
101			N-stearoyltaurine	0.47
102			N-palmitoyltaurine	0.19
103			N-palmitoleoyltaurine*	0.36
104			linoleoyl ethanolamide	0.45
105		Phospholipid Metabolism	glycerophosphorylcholine (GPC)	0.65
106			glycerophosphoethanolamine	0.73
107		Phosphatidylcholine (PC)	1-palmitoyl-2-palmitoleoyl-GPC (16:0/16:1)*	0.72
108			1-palmitoyl-2-oleoyl-GPC (16:0/18:1)	0.88
109			1-palmitoyl-2-linoleoyl-GPC (16:0/18:2)	1.18
110			1-palmitoyl-2-docosahexaenoyl-GPC (16:0/22:6)	1.14
111			1-stearoyl-2-oleoyl-GPC (18:0/18:1)	0.88
112			1-stearoyl-2-linoleoyl-GPC (18:0/18:2)*	1.19
113			1-stearoyl-2-docosahexaenoyl-GPC (18:0/22:6)	1.18
114			1,2-dilinoleoyl-GPC (18:2/18:2)	1.66
115			1-linoleoyl-2-linolenoyl-GPC (18:2/18:3)*	2.43
116			1-linoleoyl-2-arachidonoyl-GPC (18:2/20:4n6)*	1.42
117		Phosphatidylethanolamine (PE)	1,2-dipalmitoyl-GPE (16:0/16:0)*	0.74
118			1-palmitoyl-2-stearoyl-GPE (16:0/18:0)*	0.77
119			1-stearoyl-2-oleoyl-GPE (18:0/18:1)	0.87
120			1-stearoyl-2-linoleoyl-GPE (18:0/18:2)*	1.33
121			1-stearoyl-2-arachidonoyl-GPE (18:0/20:4)	1.11
122			1,2-dilinoleoyl-GPE (18:2/18:2)*	1.83
123	Lipid		1-linoleoyl-2-arachidonoyl-GPE (18:2/20:4)*	1.46
124		Phosphatidylserine (PS)	1-stearoyl-2-arachidonoyl-GPS (18:0/20:4)	1.26
125		Phosphatidylinositol (PI)	1-stearoyl-2-linoleoyl-GPI (18:0/18:2)	1.41
126			1-oleoyl-2-linoleoyl-GPI (18:1/18:2)*	1.66
127			1-stearoyl-2-arachidonoyl-GPI (18:0/20:4)	1.16
128		Lysophospholipid	2-palmitoyl-GPC (16:0)*	0.47
129			1-oleoyl-GPC (18:1)	0.84
130			1-linoleoyl-GPC (18:2)	0.53
131			1-arachidonoyl-GPC (20:4n6)*	0.42
132			2-stearoyl-GPE (18:0)*	0.50
133			1-arachidonoyl-GPE (20:4n6)*	0.47
134			1-palmitoyl-GPS (16:0)*	0.51
135			1-stearoyl-GPS (18:0)*	1.25
136			1-oleoyl-GPS (18:1)	0.33
137			1-palmitoyl-GPG (16:0)*	0.33

138		1-stearoyl-GPG (18:0)	0.46
139		1-oleoyl-GPG (18:1)*	0.14
140		1-linoleoyl-GPG (18:2)*	0.43
141		1-palmitoyl-GPI (16:0)	0.38
142		1-stearoyl-GPI (18:0)	0.47
143		1-oleoyl-GPI (18:1)*	0.31
144		1-arachidonoyl-GPI (20:4)*	0.49
145	Plasmalogen	1-(1-enyl-stearoyl)-2-arachidonoyl-GPE (P-18:0/20:4)*	1.16
146	Lysoplasmalogen	1-(1-enyl-palmitoyl)-GPE (P-16:0)*	0.67
147		1-(1-enyl-stearoyl)-GPE (P-18:0)*	0.78
148	Glycerolipid Metabolism	glycerol	0.78
149		glycerol 3-phosphate	2.56
150	Monoacylglycerol	1-palmitoylglycerol (16:0)	0.63
151		1-palmitoleoylglycerol (16:1)*	0.53
152		1-margaroylglycerol (17:0)	0.42
153		1-stearoylglycerol (18:0)	0.65
154		1-arachidonylglycerol (20:4)	2.04
155		1-docosahexaenoylglycerol (22:6)	1.95
156		2-linoleoylglycerol (18:2)	2.64
157	Diacylglycerol	diacylglycerol (12:0/18:1, 14:0/16:1, 16:0/14:1) [1]*	0.32
158		diacylglycerol (12:0/18:1, 14:0/16:1, 16:0/14:1) [2]*	0.58
159		diacylglycerol (14:0/18:1, 16:0/16:1) [1]*	0.58
160		diacylglycerol (14:0/18:1, 16:0/16:1) [2]*	0.65
161		palmitoyl-myristoyl-glycerol (16:0/14:0) [1]*	0.65
162		palmitoyl-myristoyl-glycerol (16:0/14:0) [2]	0.73
163		palmitoyl-palmitoyl-glycerol (16:0/16:0) [1]*	0.71
164		palmitoyl-palmitoyl-glycerol (16:0/16:0) [2]*	0.80
165		palmitoleoyl-palmitoleoyl-glycerol (16:1/16:1) [2]*	0.57
166		palmitoyl-oleoyl-glycerol (16:0/18:1) [1]*	0.72
167		palmitoyl-oleoyl-glycerol (16:0/18:1) [2]*	0.88
168		palmitoyl-linoleoyl-glycerol (16:0/18:2) [1]*	0.78
169		palmitoleoyl-oleoyl-glycerol (16:1/18:1) [2]*	0.64
170		oleoyl-oleoyl-glycerol (18:1/18:1) [1]*	0.65
171		oleoyl-oleoyl-glycerol (18:1/18:1) [2]*	0.82
172		linoleoyl-linoleoyl-glycerol (18:2/18:2) [1]*	1.51
173		stearoyl-arachidonoyl-glycerol (18:0/20:4) [1]*	0.64
174		oleoyl-arachidonoyl-glycerol (18:1/20:4) [1]*	0.75
175		linoleoyl-docosahexaenoyl-glycerol (18:2/22:6) [2]*	1.50
176	Dihydroceramides	N-stearoyl-sphinganine (d18:0/18:0)*	0.68
177	Ceramides	N-palmitoyl-sphingosine (d18:1/16:0)	0.82
178		N-(2-hydroxypalmitoyl)-sphingosine (d18:1/16:0(2OH))	0.62
179		N-behenoyl-sphingadienine (d18:2/22:0)*	1.34
180	Hexosylceramides (HCER)	glycosyl-N-behenoyl-sphingadienine (d18:2/22:0)*	1.81
181		glycosyl ceramide (d18:1/20:0, d16:1/22:0)*	1.23
182		lactosyl-N-nervonoyl-sphingosine (d18:1/24:1)*	0.67
183	Dihydrosphingomyelins	sphingomyelin (d18:0/18:0, d19:0/17:0)*	1.33
184		sphingomyelin (d18:1/17:0, d17:1/18:0, d19:1/16:0)	1.35

185		Sphingomyelins	sphingomyelin (d18:1/19:0, d19:1/18:0)*	1.55
186			sphingomyelin (d18:1/20:0, d16:1/22:0)*	1.46
187			sphingomyelin (d18:1/21:0, d17:1/22:0, d16:1/23:0)*	1.70
188		Sphingosines	sphingosine	0.79
189		Primary Bile Acid Metabolism	taurochenodeoxycholate	0.36
190			beta-muricholate	0.60
191			tauro-beta-muricholate	0.33
192		Secondary Bile Acid Metabolism	taurolithocholate	0.57
193			tauroursodeoxycholate	0.46
194	Nucleotide	Purine Metabolism, (Hypo)Xanthine/Inosine containing	inosine	2.04
195			hypoxanthine	1.34
196			allantoin	0.89
197			allantoic acid	1.58
198		Purine Metabolism, Adenine containing	adenosine 5'-monophosphate (AMP)	1.28
199			adenosine 3',5'-diphosphate	2.37
200			adenosine	1.39
201			2'-deoxyadenosine 3'-monophosphate	0.40
202		Purine Metabolism, Guanine containing	guanosine	1.35
203			guanine	0.62
204			7-methylguanine	1.49
205			N2,N2-dimethylguanosine	1.68
206		Pyrimidine Metabolism, Uracil containing	2'-O-methyluridine	1.63
207			5-methyluridine (ribothymidine)	1.36
208			3-ureidopropionate	0.17
209		Pyrimidine Metabolism, Cytidine containing	cytidine diphosphate	2.18
210			cytidine 5'-monophosphate (5'-CMP)	1.23
211			cytosine	0.52
212			2'-deoxycytidine 5'-monophosphate	0.59
213			2'-O-methylcytidine	1.85
214		Pyrimidine Metabolism, Thymine containing	thymidine	0.62
215		Dinucleotide	(3'-5')-adenylyluridine	2.56
216			(3'-5')-uridylyluridine	2.87
217			(3'-5')-adenylyladenosine*	2.03
218			(3'-5')-uridylylcytidine*	1.45
219	Cofactors and Vitamins	Nicotinate and Nicotinamide Metabolism	nicotinate	1.31
220			nicotinamide	1.15
221			nicotinamide adenine dinucleotide (NAD+)	1.41
222			1-methylnicotinamide	1.90
223			N1-Methyl-2-pyridone-5-carboxamide	1.53
224		Riboflavin Metabolism	flavin adenine dinucleotide (FAD)	1.45
225		Pantothenate and CoA Metabolism	coenzyme A	3.67
226		Ascorbate and Aldarate Metabolism	threonate	1.52
227		Tocopherol Metabolism	gamma-tocopherol/beta-tocopherol	0.68
228		Folate Metabolism	5-methyltetrahydrofolate (5MeTHF)	1.91
229		Tetrahydrobiopterin Metabolism	dihydrobiopterin	1.37
230		Hemoglobin and Porphyrin Metabolism	bilirubin (Z,Z)	0.59

231		Thiamine Metabolism	thiamin (Vitamin B1)	**1.24**
232			thiamin monophosphate	**1.35**
233		Vitamin A Metabolism	retinol (Vitamin A)	**1.72**
234			retinal	**2.04**
235	Xenobiotics	Benzoate Metabolism	p-cresol sulfate	**2.28**
236		Food Component/Plant	gluconate	**1.66**
237			ergothioneine	**1.49**
238			methyl glucopyranoside (alpha + beta)	**1.24**
239		Chemical	perfluorooctanesulfonate (PFOS)	**1.60**

Green: indicates significant difference ($p \leq 0.05$) between the groups shown, metabolite ratio of < 1.00

Red: indicates significant difference ($p \leq 0.05$) between the groups shown; metabolite ratio of ≥ 1.00
