# Supplementary material for: Significant alteration of liver metabolites by AAV8.Urocortin 2 gene transfer in mice with insulin resistance
Source: PLoS One. 2019 Dec 2;14(12):e0224428. doi: 10.1371/journal.pone.0224428 (PMC6886859; doi:10.1371/journal.pone.0224428)
Supplement: S7 Table — (PDF) [file pone.0224428.s008.pdf]

Supplemetary Table 7. Metabolites altered differently by AAV8.*Ucn2* vs AAV8.Empt in HFD-fed mice liver

S7-1. Amino Acids

Sub Pathway	Biochemical Name	AAV8.*Ucn2* vs Saline
Glutamate Metabolism	glutamate	1.26
	gamma-carboxyglutamate	1.25
	glutamate, gamma-methyl ester	1.50
	S-1-pyrroline-5-carboxylate	2.43
Histidine Metabolism	N-acetylhistidine	1.46
	N-acetyl-1-methylhistidine*	2.04
	1-methylhistamine	2.56
	1-ribosyl-imidazoleacetate*	1.78
	glutarylcarnitine (C5-DC)	1.71
Tyrosine Metabolism	O-methyltyrosine	1.75
	p-cresol glucuronide*	3.03
Tryptophan Metabolism	C-glycosyltryptophan	1.38
	5-hydroxyindoleacetate	1.38
Leucine, Isoleucine and Valine Metabolism	4-methyl-2-oxopentanoate	0.52
	alpha-hydroxyisovalerate	1.29
	3-methyl-2-oxobutyrate	0.53
	isobutyrylcarnitine (C4)	1.76
Methionine, Cysteine, SAM and Taurine	S-adenosylhomocysteine (SAH)	1.91
	cystine	0.59
Urea cycle; Arginine and Proline	homocitrulline	3.91
	N-alpha-acetylornithine	0.48
Polyamine Metabolism	putrescine	0.47
	(N(1) + N(8))-acetylspermidine	0.71
Guanidino and Acetamido Metabolism	guanidinosuccinate	2.46
Glutathione Metabolism	glutathione, oxidized (GSSG)	1.18
	4-hydroxy-nonenal-glutathione	1.66

Sub Pathway	Biochemical Name	AAV8.Empt vs Saline
Glycine, Serine and Threonine Metabolism	N-acetylglycine	0.72
	sarcosine	0.55
	dimethylglycine	0.71
	betaine	0.66
	serine	0.84
	threonine	0.81
Alanine and Aspartate Metabolism	N-acetylalanine	0.78
	aspartate	0.83
	asparagine	0.83
Glutamate Metabolism	S-1-pyrroline-5-carboxylate	1.96
Histidine Metabolism	histidine	0.90
	3-methylhistidine	0.57
	imidazole propionate	2.48
	formiminoglutamate	0.32
	imidazole lactate	1.42
	1-ribosyl-imidazoleacetate*	2.83
Lysine Metabolism	lysine	0.84
	N2-acetyllysine	0.65
	N6-acetyllysine	0.62
	N6,N6,N6-trimethyllysine	0.78
	5-(galactosylhydroxy)-L-lysine	0.80
	5-aminovalerate	1.89
	N-trimethyl 5-aminovalerate	0.58
Phenylalanine Metabolism	phenylalanine	0.81
	phenylpyruvate	0.53
Tyrosine Metabolism	tyrosine	0.89
	3-(4-hydroxyphenyl)lactate	0.78
Tryptophan Metabolism	tryptophan	0.85
	kynurenine	0.73
Leucine, Isoleucine and Valine Metabolism	leucine	0.79
	4-methyl-2-oxopentanoate	0.58
	beta-hydroxyisovalerate	0.75
	isoleucine	0.80
	valine	0.75
	3-methyl-2-oxobutyrate	0.62
Methionine, Cysteine, SAM and Taurine Metabolism	methionine	0.80
	methionine sulfoxide	0.67
	N-acetylmethionine sulfoxide	0.51
	S-adenosylhomocysteine (SAH)	1.46
	S-methylcysteine	0.72
	cystine	0.33

			Sub Pathway	Biochemical Name	AAV8.Empt vs Saline
			Urea cycle; Arginine and Proline Metabolism	arginine	0.73
				urea	0.73
				ornithine	0.81
				homocitrulline	2.62
				proline	0.82
				dimethylarginine (SDMA + ADMA)	0.79
				N-alpha-acetylornithine	0.51
			Polyamine Metabolism	putrescine	0.51
				spermidine	0.76
				(N(1) + N(8))-acetylspermidine	0.74
				glutathione, reduced (GSH)	6.74
				glutathione, oxidized (GSSG)	1.24
				cysteine-glutathione disulfide	0.63
				4-hydroxy-nonenal-glutathione	2.33

S7-2. Peptides

Sub Pathway	Biochemical Name	AAV8.*Ucn2* vs Saline	Sub Pathway	Biochemical Name	AAV8.Empt vs Saline
Gamma-glutamyl Amino Acid	gamma-glutamylglutamate	1.43	Gamma-glutamyl Amino Acid	gamma-glutamylleucine	1.23
	gamma-glutamylisoleucine	1.30		gamma-glutamyl-epsilon-lysine	0.76
	gamma-glutamylleucine	1.43	Dipeptide	glycylleucine	0.67
	gamma-glutamyl-epsilon-lysine	0.76		glycylvaline	0.62
	gamma-glutamylthreonine	1.94		tyrosylglycine	0.58
Dipeptide	glycylisoleucine	1.57			
	isoleucylglycine	1.78			
	leucylglycine	1.99			
	phenylalanylalanine	1.66			
	threonylphenylalanine	1.74			
	valylglutamine	2.07			
	valylglycine	1.88			
	valylleucine	2.54			
Acetylated Peptides	phenylacetylglycine	1.94			

S7-3. Nucleotides

Sub Pathway	Biochemical Name	AAV8.*Ucn2* vs Saline	Sub Pathway	Biochemical Name	AAV8.Empt vs Saline
Purine Metabolism, (Hypo)Xanthine /Inosine containing	inosine	2.04	Purine Metabolism, (Hypo)Xanthine /Inosine containing	inosine	1.75
	hypoxanthine	1.34		hypoxanthine	1.21
	allantoin	0.89		xanthosine	0.60
	allantoic acid	1.58		2'-deoxyinosine	0.59
Purine Metabolism, Adenine containing	adenosine 5'-monophosphate (AMP)	1.28		uric acid ribonucleoside	0.52
	adenosine 3',5'-diphosphate	2.37		allantoin	0.81
	adenosine	1.39	Purine Metabolism, Adenine containing	adenosine 3'-monophosphate (3'-AMP)	0.68
	2'-deoxyadenosine 3'-monophosphate	0.40		adenosine 3',5'-diphosphate	1.54
Purine Metabolism,	guanosine	1.35		2'-deoxyadenosine 3'-monophosphate	0.30
	guanine	0.62		N6-succinyladenosine	0.69

Guanine containing	7-methylguanine	1.49	Purine Metabolism, Guanine containing	guanosine	1.65
	N2,N2-dimethylguanosine	1.68		guanine	0.63
Pyrimidine Metabolism, Uracil containing	2'-O-methyluridine	1.63		7-methylguanine	1.19
	5-methyluridine (ribothymidine)	1.36		guanosine 2'-monophosphate (2'-GMP)	0.60
	3-ureidopropionate	0.17	Pyrimidine Metabolism, Uracil containing	uridine 3'-monophosphate (3'-UMP)	0.62
Pyrimidine Metabolism, Cytidine containing	cytidine diphosphate	2.18		uridine	1.33
	cytidine 5'-monophosphate (5'-CMP)	1.23		uracil	0.65
	cytosine	0.52		pseudouridine	0.62
	2'-deoxycytidine 5'-monophosphate	0.59		5,6-dihydrouridine	0.66
	2'-O-methylcytidine	1.85		5-methyluridine (ribothymidine)	1.27
Pyrimidine Metabolism, Thymine containing	thymidine	0.62		beta-alanine	0.71
Dinucleotide	(3'-5')-adenylyluridine	2.56	Pyrimidine Metabolism, Cytidine containing	cytidine 5'-monophosphate (5'-CMP)	1.27
	(3'-5')-uridylyluridine	2.87		2'-deoxycytidine 5'-monophosphate	0.48
	(3'-5')-adenylyladenosine	2.03		2'-deoxycytidine	0.64
	(3'-5')-uridylylcytidine	1.45		5-methyl-2'-deoxycytidine	0.39
			Pyrimidine Metabolism, Thymine	thymidine	0.55
				thymine	0.65
			Dinucleotide	(3'-5')-uridylyluridine	2.31
				(3'-5')-cytidylyluridine	0.66
				(3'-5')-uridylylcytidine	1.28

S7-4. Cofactors & Vitamins

Sub Pathway	Biochemical Name	AAV8.*Ucn2* vs Saline	Sub Pathway	Biochemical Name	AAV8.Empt vs Saline
Nicotinate and Nicotinamide Metabolism	nicotinate	1.31	Nicotinate and Nicotinamide Metabolism	1-methylnicotinamide	1.46
	nicotinamide	1.15	Riboflavin Metabolism	riboflavin (Vitamin B2)	0.72
	nicotinamide adenine dinucleotide (NAD+)	1.41		flavin adenine dinucleotide (FAD)	1.13
	1-methylnicotinamide	1.90	Pantothenate and CoA Metabolism	phosphopantetheine	4.33
	N1-Methyl-2-pyridone-5-carboxamide	1.53		3'-dephosphocoenzyme A	2.08
Riboflavin Metabolism	flavin adenine dinucleotide (FAD)	1.45		coenzyme A	3.63
Pantothenate and CoA Metabolism	coenzyme A	3.67		pantetheine	1.57
Ascorbate and Aldarate Metabolism	threonate	1.52			
Tocopherol Metabolism	gamma-tocopherol/beta-tocopherol	0.68			
Folate Metabolism	5-methyltetrahydrofolate (5MeTHF)	1.91			
Tetrahydrobiopterin Metabolism	dihydrobiopterin	1.37			
Hemoglobin and Porphyrin Metabolism	bilirubin (Z,Z)	0.59			
Thiamine	thiamin (Vitamin B1)	1.24			

Metabolism	thiamin monophosphate	1.35
Vitamin A Metabolism	retinol (Vitamin A)	1.72
	retinal	2.04

S7-5. Fatty Acids

Sub Pathway	Biochemical Name	AAV8.*Uch* 2 vs Saline	ochemical Nar	AAV8.Empt vs Saline
Fatty Acid, Dicarboxylate	3-methylglutarate/2-methylglutarate	1.57	3-methylglutarate/2-methylglutarate	0.90
Fatty Acid Metabolism(Acyl Glycine)	3,4-methylene heptanoylglycine	1.41	3,4-methylene heptanoylglycine	1.10
Fatty Acid Metabolism(Acyl Carnitine)	linoleoylcarnitine (C18:2)*	1.70	linoleoylcarnitine (C18:2)*	1.10
	pimeloylcarnitine/3-methyladipoylcarnitine (C7-DC)	2.43	pimeloylcarnitine/3-methyladipoylcarnitine (C7-DC)	1.35
	arachidonoylcarnitine (C20:4)	1.22	arachidonoylcarnitine (C20:4)	1.15
	erucoylcarnitine (C22:1)*	0.61	erucoylcarnitine (C22:1)*	0.73
Fatty Acid Metabolism (Acyl Choline)	palmitoylcholine	1.56	palmitoylcholine	1.01
	oleoylcholine	1.85	oleoylcholine	1.19
	palmitoloelycholine	2.14	palmitoloelycholine	0.91
	linoleoylcholine*	2.80	linoleoylcholine*	1.24
	stearoylcholine*	2.08	stearoylcholine*	1.04
	arachidonoylcholine	1.65	arachidonoylcholine	0.98

S7-6. Diacylglycerols

Sub Pathway	Biochemical Name	AAV8.*Uch* 2 vs Saline	Biochemical Name	AAV8.Empt vs Saline
	diacylglycerol (12:0/18:1, 14:0/16:1, 16:0/14:1) [1]*	0.32	diacylglycerol (16:1/18:2 [2], 16:0/18:3 [1])*	0.85
	diacylglycerol (12:0/18:1, 14:0/16:1, 16:0/14:1) [2]*	0.58	palmitoleoyl-palmitoleoyl-glycerol (16:1/16:1) [2]*	0.83
	diacylglycerol (14:0/18:1, 16:0/16:1) [1]*	0.58	palmitoleoyl-linoleoyl-glycerol (16:1/18:2) [1]*	0.30
	diacylglycerol (14:0/18:1, 16:0/16:1) [2]*	0.65	oleoyl-oleoyl-glycerol (18:1/18:1) [1]*	0.73
	palmitoyl-myristoyl-glycerol (16:0/14:0) [1]*	0.65	oleoyl-oleoyl-glycerol (18:1/18:1) [2]*	0.83
	palmitoyl-myristoyl-glycerol (16:0/14:0) [2]	0.73		

Diacylglycerol	palmitoyl-palmitoyl-glycerol (16:0/16:0) [1]*	0.71
	palmitoyl-palmitoyl-glycerol (16:0/16:0) [2]*	0.80
	palmitoleoyl-palmitoleoyl-glycerol (16:1/16:1) [2]*	0.57
	palmitoyl-oleoyl-glycerol (16:0/18:1) [1]*	0.72
	palmitoyl-oleoyl-glycerol (16:0/18:1) [2]*	0.88
	palmitoyl-linoleoyl-glycerol (16:0/18:2) [1]*	0.78
	palmitoleoyl-oleoyl-glycerol (16:1/18:1) [2]*	0.64
	oleoyl-oleoyl-glycerol (18:1/18:1) [1]*	0.65
	oleoyl-oleoyl-glycerol (18:1/18:1) [2]*	0.82
	linoleoyl-linoleoyl-glycerol (18:2/18:2) [1]*	1.51
	stearoyl-arachidonoyl-glycerol (18:0/20:4) [1]*	0.64
	oleoyl-arachidonoyl-glycerol (18:1/20:4) [1]*	0.75
	linoleoyl-docosahexaenoyl-glycerol (18:2/22:6) [2]*	1.50

S7-7. Phospholipids

Sub Pathway	Biochemical Name	AAV8.*Ucn2* vs Saline
Phosphatidyl choline (PC)	1-palmitoyl-2-palmitoleoyl-GPC (16:0/16:1)*	0.72
	1-palmitoyl-2-oleoyl-GPC (16:0/18:1)	0.88
	1-palmitoyl-2-linoleoyl-GPC (16:0/18:2)	1.18
	1-palmitoyl-2-docosahexaenoyl-GPC (16:0/22:6)	1.14
	1-stearoyl-2-oleoyl-GPC (18:0/18:1)	0.88
	1-stearoyl-2-linoleoyl-GPC (18:0/18:2)*	1.19
	1-stearoyl-2-docosahexaenoyl-GPC (18:0/22:6)	1.18
	1,2-dilinoleoyl-GPC (18:2/18:2)	1.66
	1-linoleoyl-2-linolenoyl-GPC (18:2/18:3)*	2.43
	1-linoleoyl-2-arachidonoyl-GPC (18:2/20:4n6)*	1.42
Phosphatidyl ethanolamine (PE)	1,2-dipalmitoyl-GPE (16:0/16:0)*	0.74
	1-palmitoyl-2-stearoyl-GPE (16:0/18:0)*	0.77
	1-stearoyl-2-oleoyl-GPE (18:0/18:1)	0.87
	1-stearoyl-2-linoleoyl-GPE (18:0/18:2)*	1.33

Sub Pathway	Biochemical Name	AAV8.Empt vs Saline
Phosphatidyl choline (PC)	1,2-dipalmitoyl-GPC (16:0/16:0)	0.91
Phosphatidyl ethanolamine (PE)	1,2-dipalmitoyl-GPE (16:0/16:0)*	0.80
	1-palmitoyl-2-stearoyl-GPE (16:0/18:0)*	0.77
	1-palmitoyl-2-oleoyl-GPE (16:0/18:1)	0.82
	1-oleoyl-2-arachidonoyl-GPE (18:1/20:4)*	0.90
	1-oleoyl-2-docosahexaenoyl-GPE (18:1/22:6)*	0.85
Phosphatidyl glycerol (PG)	1-palmitoyl-2-oleoyl-GPG (16:0/18:1)	1.09
Phosphatidylinositol (PI)	1-oleoyl-2-arachidonoyl-GPI (18:1/20:4) *	0.85

[illegible]	1-stearoyl-2-arachidonoyl-GPE (18:0/20:4)	**1.11**
	1,2-dilinoleoyl-GPE (18:2/18:2)*	**1.83**
	1-linoleoyl-2-arachidonoyl-GPE (18:2/20:4)*	**1.46**
Phosphatidyl serine (PS)	1-stearoyl-2-arachidonoyl-GPS (18:0/20:4)	**1.26**
Phosphatidylinositol (PI)	1-stearoyl-2-linoleoyl-GPI (18:0/18:2)	**1.41**
	1-oleoyl-2-linoleoyl-GPI (18:1/18:2)*	**1.66**
	1-stearoyl-2-arachidonoyl-GPI (18:0/20:4)	**1.16**

(green)	**Green:** indicates significant difference ($p \leq 0.05$) between the groups shown, metabolite ratio of < 1.00
(red)	**Red:** indicates significant difference ($p \leq 0.05$) between the groups shown; metabolite ratio of ≥ 1.00
